# A meta-analytical umbrella review assessing urban environment exposures and cognitive health outcomes

**DOI:** 10.1093/ageing/afag215

**Published:** 2026-07-24

**Authors:** Sophie Glover, Claire Cleland, Mike Trott, Selin Akaraci, Niamh O'Kane, Joanna Valson, Bernadette McGuinness, Ruth Hunter

**Affiliations:** Queen’s University Belfast School of Medicine Dentistry and Biomedical Sciences—Centre for Public Health, Belfast, UK; Queen’s University Belfast School of Medicine Dentistry and Biomedical Sciences—Centre for Public Health, Belfast, UK; Queen’s University Belfast School of Medicine Dentistry and Biomedical Sciences—Centre for Public Health, Belfast, UK; Queensland Centre for Mental Health Research, Faculty of Medicine, The University of Queensland, Brisbane, QLD, Australia; Queen’s University Belfast School of Medicine Dentistry and Biomedical Sciences—Centre for Public Health, Belfast, UK; Queen’s University Belfast School of Medicine Dentistry and Biomedical Sciences—Centre for Public Health, Belfast, UK; Queen’s University Belfast School of Medicine Dentistry and Biomedical Sciences—Centre for Public Health, Belfast, UK; Queen’s University Belfast School of Medicine Dentistry and Biomedical Sciences—Centre for Public Health, Belfast, UK; Queen’s University Belfast School of Medicine Dentistry and Biomedical Sciences—Centre for Public Health, Belfast, UK

**Keywords:** urban design, environmental exposures, umbrella review, cognitive decline, cognitive impairment, older people

## Abstract

**Introduction:**

Global population ageing is increasing the burden of cognitive decline and dementia. While systematic reviews have examined links between environmental exposures and cognitive outcomes, evidence remains fragmented. This umbrella review aimed to synthesise and meta-analyse systematic reviews on urban environmental exposures and cognitive health.

**Methods:**

A meta-analytic umbrella review (PROSPERO: CRD42023425117) was conducted using PubMed, Embase and PsycINFO. Systematic reviews examining urban environments and cognitive health were included. Methodological quality was assessed using the Joanna Briggs Institute tool. Meta-analytic evidence was graded (‘convincing’ to ‘not significant’) based on sample size, effect strength and bias. Narrative synthesis was also performed.

**Results:**

From 6391 records, 54 systematic reviews were included, comprising 66 meta-analysed outcomes and 270 exposure–outcome associations. Air pollution showed the strongest evidence: particulate matter 2.5 (PM2.5) (10/11 significant outcomes) and nitrogen dioxide/nitrogen oxide (5/10) demonstrated highly probable detrimental effects on cognitive health. Social relationships (5/8) showed probable beneficial effects. Green and blue spaces were associated with reduced dementia risk (3/6). Narrative findings supported these results and identified research gaps, particularly for travel behaviour (no outcomes identified).

**Conclusion:**

There is highly probable evidence linking air pollution to poorer cognitive health and probable evidence supporting social relationships. Further research is needed on urban design, noise, light exposure and underexplored pollutants (CO, SO₂). Policies should prioritise reducing air pollution and promoting social engagement to support cognitive health and dementia prevention.

## Graphical Abstract

**Graphical Abstract:**
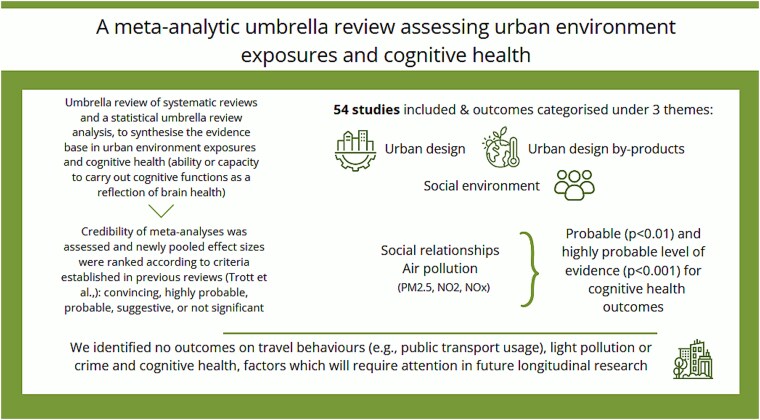

## Key points

- Air pollutants particulate matter 2.5, nitrogen dioxide and nitrogen oxide have highly probable evidence for their detrimental effect on cognitive health.
- Aspects of the social environment have probable evidence for their beneficial effect on cognitive health.
- More work is required on urban design features and travel behaviours.

## Introduction

By 2050 the global population of adults aged 60 years and older is projected to double to 2.1 billion [1]. A rapidly ageing population poses unique challenges, particularly regarding the potentially unprecedented burden on the healthcare system and its services. Consequently, it is vital that research responds to the challenges of ageing and focuses on determining factors which can promote healthy ageing to increase the proportion of time spent in good health [1]. Cognitive health is defined in this review as the ability or capacity to carry out cognitive functions such as attention and decision-making, as a reflection of brain health [2]. Cognitive health is a key consideration in public health; it is estimated that by 2050, 152 million of the world’s population aged 60 years and older will be living with dementia (~8%) [3]. Dementia is an umbrella term which indicates a decline in cognitive functions (e.g. difficulty thinking, remembering and making decisions), resulting in difficulties performing activities of daily living and communicating with others [4]. Dementia is a significant cause of disability and results in a substantial health, economic and social burden for the suffering individual, their loved ones, and wider society [3]. Wimo and colleagues (2023) reported that the global societal costs of dementia were US$1313 billion in 2019 [5]. Research is required to identify risk management strategies to alleviate the burden of dementia on ageing populations along with the healthcare system and the economy.

The Lancet Commission [6] has highlighted 14 potentially modifiable risk factors that would account for a 40% reduction in dementia prevalence if they were eliminated. This list includes factors related to or encompassing the urban environment such as air pollution, physical (in)activity and social contact [6, 7]. Urban environments provide areas to undergo physical activity [8] with an important dependency on quality of spaces. Urban environments also have important connections to the social environment, providing infrastructure which supports social cohesion [9]. There is a growing necessity to suggest sustainable interventions to better brain health in the ageing population. Considering the capacity for the urban environment to interact with modifiable risk of dementia, this prompts us to consider the evidence regarding the relationship between the urban environment and cognitive health which may reveal the capacity for environmental regenerations to contribute to brain health as we age.

Recent work by Soloveva et al. reviewed literature on the neighbourhood built environment and different cognitive outcomes in adults and older adults including the mediative or moderator effect of different environmental factors within relationships [10]. Several theoretical frameworks have also described how environmental exposures impact cognition. Cognability is a theory that emphasises that built environments can either support or hinder cognitive functions, influencing cognitive ageing [11]. Attention restoration theory suggests that natural environments help replenish cognitive resources [12], while the ecological theory of ageing underscores how environmental demands interact with an individual’s functional capacity to influence cognitive ageing [13]. Additionally, the conceptual model from Cerin et al. explains the importance of considering the effect of both the built and natural environment with regards to cognitive health [14].

While individual factors such as genetics and health behaviours play a critical role in shaping cognitive health [15], focusing exclusively on these factors may not fully address its complexity and it is important to consider the feasibility of altering health behaviours in the ageing population The urban environment has the capacity to modify exposure to environmental risk factors of dementia (i.e. air pollution), along with health behaviours. For instance, access to green space may facilitate physical activity [16, 17], while neighbourhood safety and social cohesion can influence stress and mental well-being [18]. Urban environment factors are modifiable and amenable to policy interventions, making them a strategic target for public health efforts that aim to promote cognitive health at the population level. Urban environments therefore play a significant role in shaping cognitive health, and leveraging these environments may enable us to develop interventions or policies to promote cognitive health and lower risk of dementia globally.

The definition of the urban environment varies across the literature, reflecting differences in how researchers define and measure its components. Some studies focus primarily on physical aspects, such as urban design, air quality or access to green space, while others emphasise social dimensions, including neighbourhood cohesion and safety. Urban environments have potential impacts on the social environment [19] and whether that be positive through enhancing an individual’s social support, or negative through socio-economic disadvantage, these consequences can impact on cognitive health [20, 21]. The effects of the social environment on cognitive health have also been the focus of recent systematic reviews [22, 23]. Therefore, it is important to include social environments when investigating the role of urban environment factors in cognitive health. In this umbrella review, we define urban environments as comprising both physical and natural aspects (e.g. air quality, green space, built infrastructure) and social dimensions (e.g. social support, social isolation). While individual-level cognitive risks such as genetic markers and health behaviours are widely researched [24], the impact of urban environments remains underexplored. Addressing this gap can inform urban planning and policy interventions aimed at promoting cognitive health and reducing dementia risk across populations.

To date, several systematic review studies have investigated the relationship between urban environment exposures and cognitive health. Besser et al. explored the relationship between social and built environment factors and cognition in older adults [25]. The systematic review (*n* = 25 studies) included built environment measures within several domains including density, diversity and distance to transit, and the social environment including socio-economic status, demographics, social disorder and social tie-related measures. The findings indicated a strong association between neighbourhood socio-economic status and cognition, with lower socio-economic status associated with worse cognitive outcomes. Additionally, most built environment factors showed robust associations with cognition outcomes. For instance, neighbourhoods with diverse land uses were associated with lower risk of dementia, and the presence of a neighbourhood transit stop was associated with slower cognitive decline. On the other hand, neighbourhoods in poor condition with deteriorating public spaces were associated with accelerated cognitive decline. In addition, a recent systematic review and meta-analysis by Abolhasani et al. explored the epidemiological evidence of the effects of air pollution on dementia, cognitive function and cognitive decline in the adult population [26]. Findings demonstrated a significant association between exposure to particulate matter 2.5 (PM_2.5_) and the incidence of dementia, while associations between dementia and nitrogen oxide (NO_x_), nitrogen dioxide (NO_2_) and O_3_ exposure were not statistically significant. Similarly, a critical review by Delgado-Saborit et al. highlighted the adverse effects of air pollution on cognitive function and its causal association with cognitive impairment and the risk of dementia [27]. The importance of the surrounding urban environment on cognition throughout the life-course has also been highlighted [6] as systematic reviews have also focused on nature exposure and children’s cognitive function such as Nguyen et al. systematic review and meta-analysis on children and adolescents cognitive function associations with nature [28]. The collective evidence from these systematic reviews underscores the influence of urban environment exposures on cognitive health throughout the life-course.

Considering the number of existing reviews investigating urban environment exposures and cognitive health, and the need to efficiently harness evidence to inform future policy and practice, we conducted an umbrella review and meta-analysis to synthesise the existing evidence base. Building on the recent work in Soloveva et al. [10] and the established frameworks on the urban environment and cognitive health, we wanted to perform an umbrella review with meta-analysis and narrative summaries to synthesise statistical evidence and consider findings from all environmental exposure categories and populations. The aim of this umbrella review and meta-analysis was to: (i) synthesise the evidence investigating the association between urban environment exposures and the impact on cognitive health; (ii) assess associations across age groups/categories to investigate how environmental exposures are associated with cognitive health over the life-course.

## Methods

This umbrella review and meta-analysis of systematic reviews was conducted in accordance with the Joanna Briggs Institute (JBI) guidelines for umbrella reviews [29], and the Preferred Reporting Items for Systematic Reviews and Meta-analyses (PRISMA) guidelines [30]. The protocol for this review was published in the international prospective register of systematic reviews (PROSPERO registration number CRD42023425117). There were no changes to the pre-registered protocol.

### Exposure and outcome framework

Building on the established conceptual model by Cerin and colleagues (2019) which focused on the effects of the urban environment on cognitive health [14], the research team co-developed a causal loop diagram (CLD) to illustrate the mechanistic pathways (hypothesised and evidenced) detailing the inter-relationships between the urban environment, health behaviours, health (physical and mental) and psychological factors that influence cognitive decline in ageing adults (https://kumu.io/space-cld%20/space-cld, Supplementary Data Appendix 1) [31]. The CLD informed the boundary and search terms for this umbrella review. Environmental exposures were categorised as urban design, urban design by-products (i.e. environmental pollutants, traffic levels and heat), social environment or travel behaviours [31]. See Supplementary Data Appendix 2 for detailed information on each category. The CLD also informed the outcome framework to include all types of dementia (e.g. Parkinson’s-related and frontotemporal dementia) and cognitive impairment.

### Search strategy

PubMed, Embase and PsycINFO databases were searched on 14 January 2025 (with no date limits) to identify systematic reviews focused on examining associations between the urban environment and cognitive health. Details of the full search strategy can be found in Supplementary Data Appendix 3. In brief, the search included a variety of urban environment exposures based on the included categories and nodes of the CLD [urban design, urban design by-products, social environment, travel behaviours (Supplementary Data Appendix 2)].

After removal of duplicates, each study was independently screened at both the title/abstract and full text stage by two members of the research team. In both the title/abstract and full-text screening stages, the following inclusion criteria were used:

**Population**: Any population, including children, adults and older adults.

**Independent variables (exposure variables)**: Environmental variables were informed by a CLD and included: urban design, urban design by-products, social environment and travel behaviours detailed above.

**Comparators**: Any systematic reviews that included both observational and intervention studies, therefore no specific comparators were defined.

**Outcomes**: Dependent variables, any variables that related to cognitive health outcomes, including testing cognitive function and specific conditions including mild cognitive impairment and any type of dementia (i.e. Alzheimer’s disease, frontotemporal dementia, vascular dementia and dementia with Lewy bodies, Parkinson’s disease dementia [32]).

**Study types**: Systematic reviews (with or without meta-analyses). Any other study types such as mini-reviews or scoping reviews were excluded and all types of f primary study, editorials, dissertations and opinions pieces were excluded.

Studies that were not published in English were excluded, and reference lists of all included studies were assessed to ascertain if any further reviews met the eligibility criteria.

### Data extraction

Data extraction was completed in Microsoft Excel, based on the recommendations from the JBI guidelines for umbrella reviews [29]. Two reviewers (M.T.; J.V. or S.G.) completed data extraction, and extracted information including environmental exposure type, age category as categorised by the authors [children (<18 years old), adult (18–65 years old), adults and older adults or older adult (>65 years old)] and number of studies included in the review reporting significant results. Further details of data extraction can be found in the Supplementary Data Appendix 4.

### Risk of bias assessment

Risk of bias was assessed by two authors (M.T.; J.V. or S.G.) using the JBI critical appraisal tool for systematic reviews [29]. This tool consists of 11 questions that aim to assess several aspects of a systematic review, all with an answer of ‘yes’, ‘no’, ‘unclear’ or ‘not applicable’. As the JBI tool does not recommend a systematic method of scoring the JBI checklist, the review team modified a scoring method based on methodology in line with previous umbrella reviews implemented in similar fields of research [33]. We scored the tool on a continuous scale with every ‘yes’ answer yielding one point, with a maximum score of 11 points being possible. Scores of ≤3 were categorised as ‘low quality’, 4–8 ‘moderate quality’ and ≥9 ‘high quality’ coinciding with a high, moderate or low risk of bias respectively.

### Data synthesis

A dual synthesis approach was adopted for this umbrella review. The meta-analytical synthesis allowed a re-calculation of statistical estimates for individual environmental exposures and cognitive outcomes which have been established in previous systematic reviews. These estimates were re-calculated and graded based on their credibility in line with previous umbrella reviews to provide graded evidence highlighting the relationship for certain environmental exposures and cognitive health, commonly risk of dementia.

This umbrella review aimed to summarise the broad literature base on the overall urban environment and cognitive health, with interest in a large number of individual environmental exposures. In previous systematic reviews on certain environmental exposures, meta-analysis had not been conducted. As opposed to excluding these reviews based on inability to re-calculate statistical estimates, we choose to include systematic reviews which had a narrative synthesis. We therefore also include a narrative synthesis in this umbrella review, enabling us to include a summary of evidence on environmental exposures which are not included in the meta-analytical portion of the review to broaden the evidence summary and enable us to identify prominent research gaps among environmental exposures.

#### Meta-analytical analysis

The meta-analytic umbrella review analysis was performed by M.T. To be included in the meta-analytic umbrella review, a meta-analysis needed to report primary study data for all included studies (not just the pooled effect size) and have more than one primary study per outcome. If two or more meta-analyses were found examining the same exposure and outcomes, the meta-analysis with the greatest number of studies were included. If the same number of studies were found across more than one review with the same exposures and outcomes, only the most recently published meta-analysis was included. If meta-analytic outcomes did not satisfy these criteria, they were still included and reported on within the narrative synthesis. Full details of the meta-analytical procedure can be found in Supplementary Data Appendix 5. Briefly, the DerSimonian Laird method [34] was used to re-calculate random effect pooled effect sizes in R. Heterogeneity was assessed with the *I*^2^ statistic [35], the presence of small study effect bias was tested [36] and excess significance bias was tested [36, 37].

##### Assessment of the credibility of the evidence

The credibility of meta-analyses was assessed according to stringent criteria established on previously published umbrella reviews [38–41]. Significant newly pooled effect sizes were ranked as ‘convincing’, ‘highly probable’, ‘probable’, ‘suggestive’ or ‘not significant’ based on the number of events (or participants), strength of association and the presence of several biases (see Table 1 for full criteria).

**Table 1:** Credibility assessment criteria and grading.

Grading of evidence^a^	Criteria (must fulfil all criteria)
Convincing	Statistical significance of *P* < 1^*^10^−6^, including more than 1000 cases (or more than 20 000 participants for continuous outcomes) Have the largest component study reporting a significant result (*P* < .05), have a 95% prediction interval that excluded the null Did not have large heterogeneity (*I*^2^ <50%) Showed no evidence of small study effects (*P* > .10) and excess significance bias (*P* > .10)
Highly probable	Significance of *P* < .001, including more than 1000 cases (or more than 20 000 participants for continuous outcomes) Have the largest component study reporting a statistically significant result (*P* < .05)
Probable	Significance of *P* < .01 with more than 1000 cases (or more than 20 000 participants for continuous outcomes)
Suggestive	Remaining significant associations with *P* < .05
Not significant	All outcomes with *P* > .05

^a^Outcomes with fewer than three studies were automatically downgraded by one category as Egger’s regression was not conducted.

#### Narrative synthesis of reviews

In the reporting of narrative results, we first categorise results under the following sections: urban design, urban design by-products and social environment. We did not identify any studies to be reported in the travel behaviour category. Within these sections results are further categorised into types of environmental exposure, and we discuss each study which included an outcome relating to the exposure type. We include descriptions of the potential quality of results from individual systematic reviews and meta-analyses based on our rankings of these reviews on the JBI critical appraisal tool by stating ‘high quality’, ‘moderate quality’ or ‘low quality’ in brackets when discussing each individual result. We additionally include a description of the age-category the results are associated with. For information on specific sample sizes, number of studies reported, number of studies reporting significance and the associated study designs of empirical studies of every systematic review and meta-analysis included in this review, see Supplementary Data Appendix 4.

## Results

A total of 6391 studies were identified from the database searches. Following the removal of duplicates, remaining studies were screened based on their titles and abstracts, with 287 studies being selected for full text review. The PRISMA flowchart outlining the selection process can be found in Supplementary Data Appendix 6. Following full text review, 54 reviews were included in the final umbrella review and we report 270 individual outcomes in the narrative synthesis (full list of excluded full text article with accompanying reasons can be found in Supplementary Data Appendix 7). From the 54 included reviews in this umbrella review, 10 meta-analyses had sufficient data to be included in the statistical umbrella review (66 unique outcomes). For the risk of bias assessment, the range of JBI appraisal scores was 1–11, with a median score of 6.46 (moderate quality). Full details of JBI scores can be found in Supplementary Data Appendix 8. We classified *n* = 5/54, *n* = 42/54 and *n* = 7/54 studies as low, moderate and high quality, respectively.

### Meta-analytical results

In the statistical meta-analytic umbrella review, *n* = 34/66 outcomes reached statistical significance. Regarding the credibility of evidence of the significant outcomes, *n* = 2 were classified as convincing, *n* = 23 were highly probable, *n* = 6 were probable and *n* = 3 were suggestive. Most outcomes (*n* = 40/66) had a high level of heterogeneity, *n* = 9/66 had moderate heterogeneity, while the remaining 17 had a low level of heterogeneity. Of the statistically significant outcomes, *n* = 19/34 had evidence of small study effects and *n* = 7/34 had evidence of excess significance bias. The largest effect sizes were seen for lightly polluted countries, which increased the odds ratio (OR) of dementia [OR: 2.201 95% confidence interval (CI) 1.109–4.755] with this evidence being classified as suggestive. Results which were classified as convincing included NO_2_ in urban design by-products [risk ratio (RR) for dementia: 1.049 95% CI 1.029–1.069] and low social contact in the social environment category (RR for incidence of dementia: 1.566 95% CI 1.323–1.853).

Full information on the meta-analytical results can be found in Table 2. Supplementary Data Appendices 9–11 showcase the forest plots generated from the meta-analytical aspect of the umbrella review. These plots highlight differences in effect sizes for different environmental exposures and cognitive domains. For example, in Supplementary Appendix 11, PM_2.5_ is seen to have a significant relationship with vascular dementia with a large confidence interval while in Supplementary Appendix 10. PM_2.5_ is seen to have a significant relationship with cognitive functioning with a more moderate confidence interval. In Supplementary Appendix 9, PM_2.5_ and cognitive impairment have a significant relationship with a smaller confidence interval from PM_2.5_ and cognitive functioning. Additionally, the figures highlight differences in results per study type, cohort/longitudinal or cross-sectional. PM_2.5_ for example has a larger effect size in cross-sectional studies than cohort/longitudinal studies however, the confidence interval is larger for cross-sectional studies (Supplementary Appendix 10). Similar patterns are observed for urban design features and the social environment. All meta-analytical results are additionally highlighted in a heat map in Supplementary Data Appendix 12.

#### Urban design

We reported six meta-analytical results for green and blue space and dementia, *n* = 3/6 of these were classified as highly probable while the remaining *n* = 3/6 were not significant. We also reported meta-analytical results for land use/cover. One result on land use and cognitive impairment was significant and classified as highly probable. The remaining results were on land use and dementia, *n* = 1/3 of these were classified as highly probable while *n* = 2/3 were not significant.

#### Urban design by-products

General air pollution [air pollution of an unspecified constitute or combined constitute (such as SO2, CO and PM2.5) which was stated as ‘air pollution’ in empirical reviews] was significantly associated with dementia (RR: 1.133 95% CI 1.081–1.188) (highly probable evidence) but not significantly associated with Alzheimer’s disease or vascular dementia. Heavily polluted countries were not significantly associated with Alzheimer’s disease while lightly polluted countries were significantly associated (suggestive evidence).

We reported ten individual meta-analytical results for NO_2_ and NO_x_, *n* = 5/10 of these were statistically significant. NO_2_ was associated with dementia (probable evidence). Additionally, NO_2_ when comparing Tertile 1 to Tertile 2, and comparing Tertile 1 to Tertile 3 was significantly associated with dementia (convincing and highly probable evidence respectively). Similarly, NOx when comparing Tertile 1 to Tertile 2, and comparing Tertile 1 to Tertile 3 was significantly associated with dementia (highly probable evidence).

Particulate matter 10 (PM_10_) was significantly associated with a cognitive outcome in *n* = 2/7 results (cognitive test scores or cognitive function, both results being highly probable). PM_2.5_ was significantly associated with a cognitive outcome in *n* = 10/11 results {*n* = 3 probable, *n* = 7 highly probable evidence for example there was highly probable evidence that PM_2.5_ was associated with dementia [hazard ratio (HR): 1.396 95% CI 1.225–1.592]}. We report one meta-analytical outcome for particulate matter, this being significantly associated with higher risk ratio of dementia (highly probable evidence).

Carbon monoxide (CO) was significantly associated with dementia when comparing Tertile 1 to Tertile 3 (highly probable evidence). However, CO was not significant when comparing Tertile 1 and Tertile 2. We included seven individual results on ozone (O_3_) however, only *n* = 1/7 of these were significant. O_3_ was associated with cognitive function (RR: 1.191 95% CI 1.036–1.37) (suggestive evidence). Finally with regards to air pollution, we report two results for sulphur dioxide (SO_2_), *n* = 1/2 of these were significant (highly probable evidence).

We additionally included meta-analytical results for noise exposure. One of three results for environmental noise exposure were significant, noise being associated with higher

OR for cognitive impairment (OR: 1.4 95% CI 1.194–1.641) (highly probable evidence).

**Table 2:** Meta-analytical results.

Exposure type													
Exposure type	Environmental exposure type	Cognitive outcome	Study type(s)	Total included studies	Total participants	Effect size type	(95% CI)	*P*	*I* ^**2**^	Small study effect	Excess significance bias	PI	Level of evidence
							**(95% CI)**						
UGBS													
UGBS	Greenness (NDVI)	Dementia	Cross-sectional	2	478 005	RR	(0.936–0.963)	<.000	0.01	Yes	Yes	0.925–0.974	Highly probable
							0.995						
			Cohort/longitudinal	5	65 171 430	OR	(0.943–1.051)	.865	87.27	NS	NS	0.833–1.190	NS
							0.939						
	Green space, blue space and parks		Cohort/longitudinal	4	62 666 248	RR	(0.913–0.965)	<.00	90.94	Yes	No	0.886–0.994	Highly probable
							0.946						
	NDVI, area of green space or green space accessibility		Cohort/longitudinal	9	65 399 147	OR	(0.930–0.961)	<.000	52.00	No	No	0.917–0.976	Highly probable
							0.928						
	Area of green space		Cohort/longitudinal	4	227 717	OR	(0.839–1.026)	.143	36.35	NS	NS	0.794–1.084	NS
							1.028						
	Accessibility of green space		Cohort/longitudinal	2	8341	OR	(0.829–1.275)	.800	0.00	NS	NS	0.829–1.275	NS
							1.467						
Land use	Land use/land cover	Cognitive impairment	Cross-sectional	2	9929	RR	(1.222–1.761)	<.00	0.00	No	No	1.222–1.761	Highly probable
							1.207						
		Dementia		3	34 731	RR	(0.853–1.710)	.289	66.84	NS	NS	0.025–58.323	NS
							1.008						
			Cohort/longitudinal	2	354 502	RR	(0.940–1.081)	.824	0.00	NS	NS	0.940–1.081	NS
							1.093						
	Distance to major roads			3	2 728 744	RR	(1.059–1.129)	<.000	71.56	Yes	No	1.034–1.156	Highly probable
							(1.059–1.129)						
Air pollution													
Air pollution	Air pollution exposure	Alzheimer’s disease	Cohort/longitudinal	14	12 614 523	OR	(1.086–1.606)	.005	99.20	No	No	0.601–2.900	NS
							1.133						
		Dementia		12	13 284 070	RR	(1.081–1.188)	<.000	88.75	Yes	No	0.978–1.313	Highly probable
							1.100						
		Vascular dementia		3	483 628	RR	(0.948–1.278)	.210	89.00	NS	NS	0.205–5.894	NS
							1.055						
	Heavily polluted countries	Alzheimer’s disease		11	12 409 645	OR	(0.963–1.157)	.250	92.62	NS	NS	0.783–1.422	NS
							2.201						
	Lightly polluted countries			3	204 878	OR	(1.109–4.755)	.045	99.55	No	No	0.000–46534.685	Suggestive
							1.691						
	CO T1 vs T2	Dementia		2	35 702	RR	(0.728–3.926)	.221	79.35	NS	NS	0.728–3.926	NS
							1.515						
	CO T1 vs T3			2	35 702	RR	(1.347–1.703)	<.000	0.00	No	No	1.347–1.703	Highly probable
							1.003						
	NO_2_	Alzheimer’s disease		4	505 866	OR	(0.893–1.127)	.954	86.92	NS	NS	0.596–1.689	NS
							1.009						
	NO_2_ (per 10ug/m3 increment)	Cognitive function	Cohort/longitudinal	6	61 420	RR	0.936–1.089	.808	47.51	NS	NS	0.884–1.153	NS
							1.037						
	NO_2_	Dementia		9	16 788 028	HR	(1.009–1.067)	.011	93.96	No	No	0.954–1.128	Probable
							1.049						
	NO_2_ T1 vs T2			6	3 262 163	RR	(1.029–1.069)	<.000	14.04	No	No	1.008–1.092	Convincing
							1.185						
	NO_2_ T1 vs T3			6	3 262 163	RR	(1.098–1.280)	<.000	84.01	No	No	0.938–1.497	Highly probable
							1.017						
	NO_2_/NO_x_ (per 5 ppb increment)	Cognitive impairment		7	307 300	RR	(0.989–1.045)	.232	90.07	NS	NS	0.936–1.105	NS
							−0.130						
	NO_2_ (per 10ug/m3 increment)	Cognitive test scores	Cohort/longitudinal	6	25 669	Beta	(0.000; 0.051)	.160	95.83	NS	NS	−0.580; 0.320	NS
							1.055						
	NO_x_	Dementia		5	544 335	HR	(0.999–1.114)	.055	85.45	NS	NS	0.886–1.255	NS
							1.094						
	NO_x_ T1 vs T2			3	355 577	RR	(1.042–1.149)	<.000	19.65	No	No	0.695–1.721	Highly probable
							1.264						
	NO_x_ T1 vs T3			3	355 577	RR	(1.133–1.410)	<.000	20.96	No	No	0.456–3.508	Highly probable
							1.007						
	O_3_			5	2 650 306	RR	(0.947–1.070)	.829	88.06	NS	NS	0.824–1.231	NS
							1.000						
	O_3_ (per 5 ppb increment)	Cognitive impairment		4	2 644 151	RR	(0.950–1.053)	.991	91.62	NS	NS	0.797–1.253	NS
							1.191						
	O_3_ (per 10ug/m3 increment)	Cognitive function	Cohort/longitudinal	5	67 674	RR	1.036–1.37	.014	92.18	Yes	No	0.885–1.604	Suggestive
							1.081						
	O_3_	Cognitive functioning	Cohort/longitudinal	4	19 613	OR	(0.870–1.343)	.484	75.35	NS	NS	0.732–1.595	NS
							0.132						
	O_3_ (per 10ug/m3 increment)	Cognitive test scores	Cohort/longitudinal	3	20 172	Beta	(0.000; 0.747)	.675	97.88	NS	NS	−1.064; 1.327	NS
							0.983						
	O_3_ T1 vs T2	Dementia		5	2 650 306	RR	(0.922–1.048)	.602	81.54	NS	NS	0.794–1.271	NS
							0.977						
	O_3_ T1 vs T3			5	2 650 306	RR	(0.873–1.095)	.695	76.08	NS	NS	0.676–1.413	NS
							1.573						
	PM_10_			2	350 844	RR	(0.545–4.540)	.402	82.44	NS	NS	NA	NS
							1.053						
	PM_10_ T1 vs T2			2	356 999	RR	(0.857–1.294)	.624	8.29	NS	NS	0.857–1.294	NS
							1.617						
	PM_10_ T1 vs T3			2	356 999	RR	(0.601–4.353)	.341	80.02	NS	NS	0.601–4.353	NS
							1.338						
	PM_10_	Cognitive functioning	Cohort/longitudinal	5	471 301	OR	(1.064–1.682)	<.000	75.24	Yes	No	0.839–2.132	Highly probable
							1.338						
	PM_10_	Cognitive functioning	Cross-sectional	4	11 214	OR	0.925–1.298	.289	79.00	NS	NS	0.810–1.482	NS
							1.066						
	PM_10_ (per 10ug/m3 increment)	Cognitive impairment	Cohort/longitudinal	4	30 604	RR	(0.958–1.185)	.240	78.99	NS	NS	0.872–1.303	NS
							−0.093						
	PM_10_ (per 10ug/m3 increment)	Cognitive test scores	Cohort/longitudinal	6	29 073	Beta	(0.000; −0.030)	.004	82.42	Yes	Yes	−0.222; 0.037	Highly probable
							2.006						
	PM_2.5_	Vascular dementia		8	14 050 098	HR	(1.298–3.102)	.002	96.76	No	No	0.505–7.973	Probable
							1.396						
	PM_2.5_	Dementia		17	40 218 410	HR	(1.225–1.592)	<.000	98.93	Yes	No	0.872–2.235	Highly probable
							1.469						
	PM_2.5_	Alzheimer’s disease		12	1 656 291	HR	(1.217–1.774)	.000	98.61	No	No	0.779–2.770	Highly probable
							0.984						
	PM_2.5_	Mild cognitive impairment	Not reported	9		OR	(0.952–1.018)	.354	70.45	NS	NS	0.903–1.073	NS
							1.116						
	PM_2.5_	Cognitive functioning	Cohort/longitudinal	10	548 968	OR	(1.043–1.194)	.001	74.58	Yes	Yes	0.971–1.283	Probable
							1.671						
	PM_2.5_	Cognitive functioning	Cross-sectional	7	26 962	OR	(1.259–2.216)	<.000	80.32	Yes	No	0.899–3.106	Highly probable
							−0.299						
	PM_2.5_ (per 10ug/m3 increment)	Cognitive test scores	Cohort/longitudinal	8	42 350	Beta	(0.000; −0.160)	<.000	94.84	Yes	Yes	(−0.647; 0.049)	Highly probable
							1.152						
	PM_2.5_ (per 10ug/m3 increment)	Cognitive impairment	Cohort/longitudinal	7	89 644	RR	(1.089–1.220)	<.000	92.02	Yes	Yes	1.004–1.323	Highly probable
							1.077						
	PM_2.5_ (per 5ug/m3 increment)	Cognitive impairment	Cohort/longitudinal	11	12 656 337	RR	(1.027–1.130)	.002	82.16	No	No	0.945–1.228	Probable
							1.064						
	PM_2.5_ T1 vs T2	Dementia		11	13 155 894	RR	(1.029–1.101)	<.000	81.40	Yes	No	0.966–1.173	Highly probable
							1.134						
	PM_2.5_ T1 vs T3			11	13 155 894	RR	(1.074–1.197)	<.000	85.35	Yes	No	0.971–1.323	Highly probable
							1.088						
	PM_x_			12	81 647 185	RR	(1.059–1.117)	<.000	97.30	Yes	No	1.004–1.179	Highly probable
							1.388						
	SO_2_	Cognitive functioning	Cohort/longitudinal	3	36 223	OR	1.272–1.515	<.000	0.00	Yes	No	1.272–1.515	Highly probable
							−1.018						
	SO_2_ (per 10ug/m3 increment)	Cognitive test scores	Cohort/longitudinal	3	20 172	Beta	(−3.000; 0.757)	.261	99.76	NS	NS	−4.555; 2.519	NS
Noise exposure							1.400						
Noise exposure	Environmental noise	Cognitive impairment	Cross-sectional	3	9565	OR	(1.194–1.641)	<.000	0.00	Yes	No	1.194–1.641	Highly probable
							−0.116						
		Reading and language abilities		4	13 802	Beta	(−0.334–0.103)	<.299	37.34	NS	NS	−0.888-0.657	NS
							1.092						
		Dementia	Cohort/longitudinal	2	2 616 994	RR	(0.937–1.271)	.260	98.65	NS	NS	0.839–1.42	NS
							(0.937–1.271)						
Social relationships													
Social relationships	Combination of structural and functional aspects of social relationships	Cognitive decline	Cohort/longitudinal	7	14 027	OR	(1.023–1.414)	.025	81.66	No	Yes	0.762–1.898	Suggestive
							1.144						
	Functional aspects of social relationships	Cognitive decline		8	5367	OR	(0.997–1.313)	.056	66.00	NS	NS	0.786–1.666	NS
							1.579						
	Loneliness	Incidence dementia		3	3252	RR	(1.192–2.092)	.001	0.00	No	No	1.192–2.092	Probable
							1.246						
	Satisfaction with social network			4	6207	RR	(0.956–1.624)	.103	50.30	NS	NS	0.462–3.363	NS
							1.566						
	Social contact			8	15 762	RR	(1.323–1.853)	<.000	0.00	No	No	1.323–1.853	Convincing
							1.167						
	Social network size			5	7750	RR	(0.923–1.477)	.197	63.96	NS	NS	0.559–2.439	NS
							1.406						
	Social participation			6	7714	RR	(1.132–1.745)	.002	31.23	Yes	No	0.845–2.338	Probable
							1.082						
	Structural aspects of social relationships	Cognitive decline		21	26 430	OR	(1.050–1.114)	<.000	72.63	Yes	Yes	1.005–1.164	Highly probable
							(1.050–1.114)						

ES = effect size; *I*^2^ = measure of study heterogeneity; NDVI = normalised difference vegetation index; NS = not significant ; O_3_ = ozone; P = level of significance; PI = prediction interval; PM_10_ = particulate matter less than 10 μm in diameter; PM_2.5_ = particulate matter less than 2.5 μm in diameter; ppb = parts per billion; SCD = subjective cognitive decline; SMD = standardised mean difference; T1 = 1^st^ tertile; T2 = 2^nd^ tertile; T3 = 3^rd^ tertile; ug = microgram; UGBS = urban green and blue space.

#### Social environment

We report eight results for social relationships (including social support, loneliness, social participation etc.,), *n* = 5/8 were significant (*n* = 1/5 suggestive, *n* = 2/5 probable, *n* = 1/5 highly probable, *n* = 1/5 convincing evidence). Structural and functional aspects of social relationships were significantly associated with cognitive decline (suggestive evidence) and loneliness was associated with higher incidence of dementia (RR: 1.579 95% CI 1.192–2.092) (probable evidence). Low social contact was associated with incidence of dementia (convincing evidence). Finally, less social participation was associated with incidence of dementia (probable evidence) and structural aspects of social relationships were associated with cognitive decline (highly probable evidence). Aspects of social relationships which were not significantly associated with cognitive outcomes in our meta-analysis include functional aspects of social relationships, satisfaction with social network and social network size.

### Narrative synthesis of results

A total of 270 individual outcomes were extracted from 54 reviews, some reviews including exposures from multiple categories of urban design, urban design by-products or social environment. No results from included reviews were seen as appropriate for a travel behaviour category based on the categories of the CLD which the search strategy for this umbrella review is based upon (see Supplementary Data Appendix 2). The travel behaviour category criteria specifically include active travel, public transport usage, private vehicle usage and private car ownership. Despite some included reviews reporting on public transport for example, they report on public transport accessibility as opposed to public transport usage which is classified under urban design. Urban design by-products had the most outcomes (*n* = 196) followed by urban design (*n* = 44) and the social environment (*n* = 30). The largest environmental exposure categories in terms of individual outcomes were PM_2.5_ (*n* = 48) and NO_2_ and NO_x_ (*n* = 29) while traffic safety (*n* = 1) and social isolation and loneliness (*n* = 2) had some of the smallest number of outcomes. We did not identify any systematic review with outcomes relating to crime, light pollution, car usage or active transport that met our eligibility criteria.

**Figure 1:**
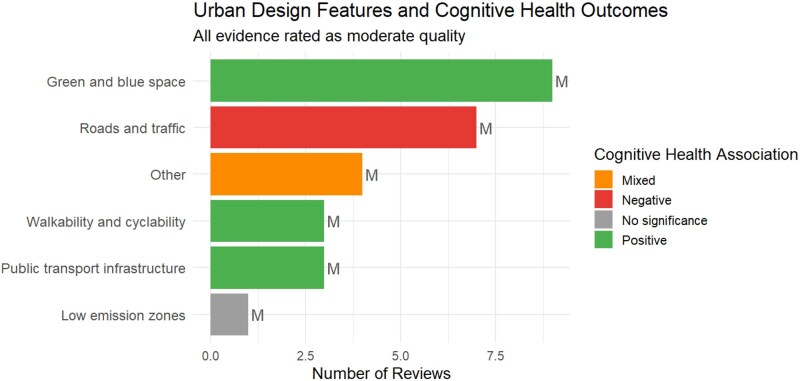
Visual summary of the narrative results for urban design. The figure represents a summary level of the findings. ‘M’ is representative of the summary of the quality rating, all quality summaries were moderate reflecting the majority of included reviews being of moderate quality.

**Figure 2:**
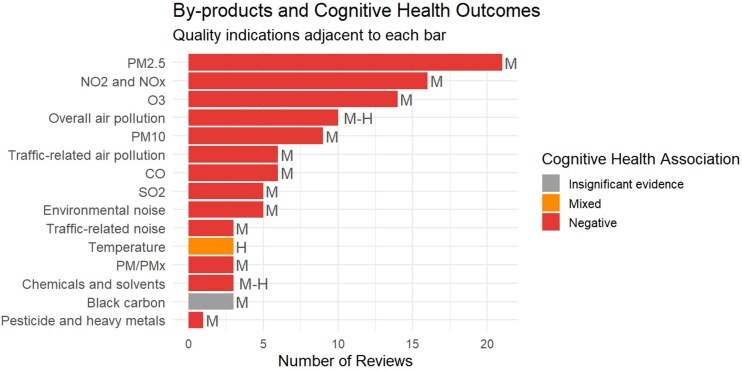
Visual summary of the narrative results for environmental by-products. The figure represents a summary level of the findings. The summary level of the quality ratings here is ‘M’ for moderate, ‘M-H’ for moderate-high and ‘H’ for high.

**Figure 3:**
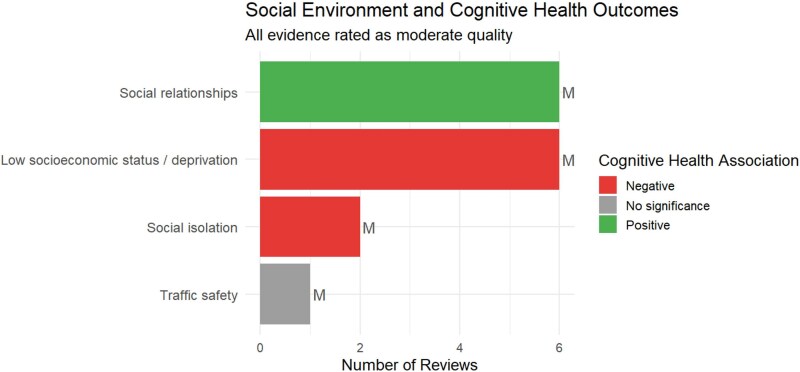
Visual summary of the narrative results for social environment. The figure represents a summary level of findings. The summary level of the quality ratings here is all ‘M’ for moderate.

A visual summary of the narrative synthesis is provided in Figures 1–3. The figures include the number of reviews which provided evidence of an individual environmental exposure, a summary of their direction of effect on cognitive health and their quality rating. These figures represent summary levels of evidence from the narrative review where in if the majority of studies were of moderate quality, the representative quality for that environmental exposure is described as moderate for example, or if the majority of studies found that an exposure was detrimental to cognitive health outcomes, the representative directionality is ‘bad’. For results relating to specific cognitive health outcomes, please refer to Supplementary Data Appendix 4. A comprehensive discussion of each included reviews findings can be found in Supplementary Data Appendix 13.

### Risk of bias assessment

The majority of individual narrative results were from reviews classified as moderate quality (*n* = 211/270) while *n* = 12/270 results were from low quality reviews and *n* = 47/270 were from high quality reviews. With regards to environmental exposure categories, *n* = 3/44 results from urban design were from high quality reviews and the remaining *n* = 41/44 were from moderate quality reviews. For urban design by-products, *n* = 11/196 results were from low quality reviews, *n* = 141/196 were from moderate quality reviews and *n* = 44/196 were from high quality reviews. Notably, the results on temperature and chemicals and solvents were majorly from reviews of high quality (*n* = 3/5 and *n* = 6/9 respectively). Finally, regarding the social environment, *n* = 1/30 results were from low quality studies and *n* = 29/30 were from moderate quality studies. For further details on the risk of bias assessment, see Supplementary Data Appendix 8 and Appendix  14.

## Discussion

This umbrella review quantitatively and narratively synthesised evidence on the relationship between urban environment exposures and cognitive health. In our meta-analysis, we notably report that PM_2.5_, NO_2_ and NO_x_, have highly probable levels of evidence for their association with cognitive health outcomes and social relationships have probable levels of evidence for their association with cognitive health (representable summary levels of evidence). We additionally report highly probable levels of evidence for half (*n* = 3/6) of our meta-analytic outcomes for urban green and blue space. Our narrative findings indicate that urban design by-products (*n* = 196 individual results) received the most attention in current research while urban design (*n* = 44 results) features and aspects of the social environment (*n* = 30 results) could be considered under researched. Additionally, despite the large amounts of evidence suggesting by-products such as aspects of air pollution, have adverse impacts on cognitive health, we did not identify any current reviews on light pollution, crime perception or public transport usage for example.

### Evidence synthesis

#### Urban design

The meta-analytical findings of this review suggest that urban green and blue may be associated with lower risk of dementia with *n* = 3/6 significant results of highly probable evidence. This is in line with recent work on these spaces which has seen significance between green and blue space exposure and cognitive health such as Liu et al. study which found positive effects of urban green space exposure on children’s cortical indicators [42]. However, other studies have found no statistical significance between green space exposure and cognitive health. In Nguyen et al. meta-analysis on children and adolescents’ cognitive function, significance was only found for experimental studies (those which examined cognitive function before and after a green intervention) and not for correlation studies (those which assessed the correlation between observational green space exposure and cognitive function) [28]. Upon interpreting results, it is important to consider that measurements of green space may not be accurately representing accessible space for populations; accessibility being an important factor for usage [43]. For example, participants may live close to green space according to their localised normalised difference vegetation index but may not be able to access this space. In addition, the majority of studies in this area examine the impact of exposure to greenness (e.g. can an individual see an area of greenness from their home investigated in correlational studies) as opposed to actual time spent in spaces (experimental or interventional studies). Exploring the potential difference in cognitive effects of accessibility of green space (potentially implying higher likelihood of usage) and having green space near residence (visual effects, pollutant mitigation effects) is important in future work on the subject. Literature has identified protective associations of green and blue spaces on cognition throughout the life-course, some of which have been summarised in a recent systematic review [44]. However, it is important that future work consolidates these findings and explores capacity of spaces to act within mechanistic pathways.

Narrative findings indicated that proximity to roads (often used as a proxy for exposure to traffic related air pollution and/or noise exposure) had a negative impact on cognitive health and operated as a risk factor for dementia. These findings are in line with those of Lancet Commission of Dementia prevention, intervention and care which identified air pollution as one of the modifiable risk factors for dementia [6, 7]. Recent work in Cerin et al. also highlights the complexity between relationships of the built and natural environment, traffic-related air pollution and socio-economic status [45]. Additionally for urban design, the neighbourhood environment and walkability had positive effects on cognitive health in later life. Chen et al. reported that street connectivity was associated with better cognition [46], in line with the work of Tani et al. which highlighted that the presence of pavements was a significant protective factor for dementia (fully adjusted HR: 0.71) [47], indicating that although proximity to roads is a risk factor for dementia, having a walkable neighbourhood could help negate this risk. Considering the neighbourhood environment and walkability were not included in a large number of included reviews, further high quality longitudinal work is required. Strengthening this evidence base could contribute to neighbourhood infrastructure developments to better cognitive health in the ageing population.

Interestingly, a recent large cohort study found that whilst objective walkability was not associated with walking in a cohort of older adults, perceived walkability was associated with walking and had a significant protective relationship with cognitive ageing [48]. This indicates that it may be the perception of walkability that has more of an effect than objective walkability. In addition to a walkable neighbourhood, neighbourhood aesthetics were associated with better cognition in our review. It is possible that the perception of living in a ‘pleasant’ environment or an environment being perceived as being walkable could encourage people to participate in more physical activity outdoors. Conversely, the perception of having neighbourhood physical disorder may result in people staying at home due to safety concerns, therefore spending less time outdoors and being less physically active [49, 50]. Considering the importance of neighbourhood infrastructure and safety for physical activity, a modifiable risk factor of dementia [6, 7, 51], care should be taken to ensure neighbourhood developments consider the consequences on subjective feelis about the neighbourhood.

#### Urban design by-products

In our meta-analytical results, *n* = 48/66 results were in the urban design by-product category. Of the outcomes on PM_2.5_, *n* = 7/11 were highly probable and *n* = 3/11 were probable. For NO_2_ and NO_x_, *n* = 3/10 outcomes were highly probable, *n* = 1/10 was probable and *n* = 1/10 was convincing. These findings specifically highlight PM_2.5,_ NO_2_ and NO_x_ as risk factors for dementia, in agreement with the work of Parra et al. who examined the UK Biobank in relation to air pollution exposure and incident dementia [52]. Potential mechanisms of cognitive effect were identified in this paper including the effect of air pollution on inflammation, oxygen related and/or neuroendocrine stress responses, or activation of the hypothalamic–pituitary–adrenal axis [53–55]. Some meta-analytical results for other exposures such as PM_10_ and SO_2_ did find at least one significant outcome; however, the smaller number of outcomes here limit our ability to suggest these pollutants as strongly associated with cognitive health. A recent systematic review and meta-analysis and a UK based multi-cohort wise analysis did not find significance between PM_10_ exposure and incident dementia [56, 57] which prompts further research to consolidate findings and explore potential difference in health effects based on particulate size.

In our meta-analytical findings, we report a highly probable association of noise and specifically cognitive impairment which is in line with recent literature [58–60]. Additionally, in our narrative synthesis we included three reviews which included studies on the effect of road traffic noise on cognitive health. Two of these reviews found that higher noise exposure was associated with worse cognitive outcomes (higher likelihood of mild cognitive impairment in older adults, worse cognitive performance in adults) [61, 62] and one review had mixed findings [63]. A recent study exploring environmental exposures on dementia outcomes and brain volumes did not find any significant associations between environmental noise exposure and any dementia related outcome [64] which also suggests there is mixed or conflicting evidence in the field. Despite this, hearing loss has been highlighted as a risk factor of dementia in recent Lancet Commission of Dementia reports [6, 7]. When considering integrating research suggestions into urban policies, working to reduce car dependency in urban areas for example may enable a reduction in the adverse effects created by traffic-related pollution including noise, to promote cognitive health.

#### Social environment

In meta-analysis, social contact and the structural aspects of social relationships (e.g. social integration and reach of social network) were found to have a convincing level of evidence for incidence of dementia and highly probable evidence for cognitive decline respectively [65]. In addition, loneliness and low social participation had a probable level of evidence for incidence of dementia. These findings align with the Lancet Commission which identified social isolation as a modifiable risk factor for dementia [6, 7] social relationships potentially operating to enhance cognitive reserves through mechanisms such as increasing social-related physical activity. The social environment of the neighbourhood may be a crucial factor to maintaining cognitive health as we age. Ensuring urban design considers how features such as roads, pavements and green space developments impact neighbourhoods is therefore important.

Several reviews included within the narrative synthesis examined socio-economic status and commonly highlighted it as a risk factor for cognitive impairment and dementia. Deprived areas notably suffer from higher air pollution levels [66, 67], lower quality of green spaces [68] and more issues in terms of anti-social behaviour or crime [69], factors relating to by-products, urban design and social environments respectively. Ensuring deprivation status is considered in future decision making on urban design interventions and policy will be important to ensure all populations gain the potential benefits of interventions.

#### Travel behaviours

We did not identify any systematic review or meta-analyses in our search which could be included in a travel behaviour category as they did not specifically analyse active travel, public transport usage, private vehicle usage or private car ownership. There is literature which has described the effects of active travel levels for example on cognitive health [70–72]. Herfet et al. systematic review which was not included in our review, but included eleven studies on active travel levels and cognitive function in children, adolescents and older adults [72]. Herfet et al. findings were mixed with around half of studies reporting an association of active travel and a specific cognitive domain such as executive functioning. Martínez-Gómez et al. empirical study reported that active school travel among adolescent females was associated with higher verbal ability scores [73]. Additionally, the one included study on older adults in Herfet et al. reported mixed findings as higher cognitive performance was associated with engaging in recommended levels of physical activity by active travel and leisure-time physical activity but not associated with engaging in recommended levels of physical activity through active travel alone [74]. This suggests active travel may be able to support older adults to engage in physical activity to protect their cognition. We identified three outcomes within the urban design section which pertained to the presence or accessibility to public transport; however, no review reported on the usage of public transport. There is evidence provided in the urban design category on travel infrastructure however, considering the potential direct and indirect consequences of travel behaviours (e.g. generating traffic-related pollution, physical (in)activity), future research explicitly on travel behaviours and cognitive health is warranted.

### Implications for research, policy and practice

#### Urban design

Taking into consideration the potential benefits of green spaces for physical and mental health and well-being, it is imperative that this evidence base expands to include more high quality research on cognitive health. Future work should also aim to investigate evidence of hypothesised mechanistic pathways through the implementation of longitudinal studies [8, 44, 75].

No outcomes of urban design investigated streetlight density and very few reviews included outcomes relating to blue space, cycling infrastructure or public transport accessibility. Blue space exposure has been reported to have similar potential benefits to green space exposure in the literature for the brain [76] but despite this, there is a distinct lack of research on the topic. Promotion of blue space usage requires further research. There is also a need for future work on the potential effects of light pollution and public transport accessibility considering the associations between light pollution and cognitive impairment in recent literature [77], and the potential that poor public transport accessibility encourages private vehicle usage which has cascade effects on the promotion of by-products (such as traffic-related air and noise pollution). Additionally, the majority of included reviews of urban design investigated effects on adults and older adults’ cognitive health. It is important to note that research on children’s cognitive health is also of interest in terms of urban design, considering urban design can play an important role in the life of young people, namely school children through either providing places of play, or providing routes for travel to school or activities. All included studies providing urban design outcomes were of a moderate quality. To ensure future research can provide a strong evidence base which can be influential on urban development projects, conducting high quality research is important.

#### Urban design by-products

Findings from the current review support the Lancet Commission by highlighting exposure to air pollution (specifically PM_2.5_, NO_2_, NO_x_) has the capacity to increase the risk of dementia [6, 7]. However, more longitudinal research is required to investigate the impact of other air pollutants including CO, SO_2_ and O_3_. We included outcomes of noise pollution and temperature within this review however, further work is required to strengthen the evidence base. It is also of interest that further study includes work on the effects of urban water and soil on cognitive health, considering the small number of outcomes relating to these exposures we identified. Similar to urban design features, it is imperative that mechanistic pathways by which by-products can enable cognitive decline and dementia pathology is investigated. Finally, the effects of air pollution exposure for example were examined over the life-course within included reviews and meta-analyses. However, other exposures such temperature were solely investigated in adults and older adults highlighting the necessity for life-course longitudinal research to strengthen the evidence base.

#### Social environment

Overall, we report probable evidence on the relationship between the social environment and cognitive health. Further high quality and longitudinal research would help strengthen the current evidence base. Policies and interventions should be implemented to reduce health and social inequalities considering the potential adverse effects of social-economic status and deprivation on cognitive health. Additionally, we identified no reviews which met the eligibility criteria on crime safety perception and cognitive health; an area which may require attention in future research. Providing further evidence on the relationship between the social environment and cognitive health will ensure that urban design policies consider how decisions may have unintended impacts.

#### Travel behaviours

We did not identify any review which met the eligibility criteria which investigated travel behaviours such as public transport usage, active travel or private vehicle usage and cognitive health. Considering travel behaviours have relationships with modifiable risk factors of dementia such as air pollution and physical (in)activity [6, 7], future research should conduct high quality, longitudinal study to supplement the limited evidence base. This research can provide policymakers with information regarding the cognitive health impacts of potential public transport infrastructure developments.

### Strength and limitations

The main strength of this review is the combined meta-analytic and narrative approach that was implemented to ensure all relevant evidence was included. Statistical summarises were graded based on criteria utilised in previous umbrella reviews and the narrative synthesis includes comprehensive data extraction, narration and visual summaries. We took a life-course approach to investigate the association between urban environment factors and cognitive health by including a range of age groups highlighted in the narrative synthesis. All included studies underwent a risk of bias assessment with the JBI tool for systematic reviews, the quality of studies (high, moderate, low) was synthesised and included in the narrative synthesis. For the meta-analytical synthesis, the JBI assessment was not included in Table 2 as we performed an independent credibility rating of each meta-analytical outcome in line with previous umbrella reviews [38–41].

The credibility of the evidence synthesised in this review must be considered in the context of the quality of included reviews and the heterogeneity observed across meta-analytic outcomes. Regarding risk of bias, the distribution of quality was uneven across exposure categories, with air pollution evidence having several high-quality reviews, whereas the social environment and urban design categories were exclusively supported by moderate-quality reviews, which limits the confidence with which classifications can be interpreted in those domains. Common limitations such as insufficient reporting of search strategy components and incomplete assessment of publication bias in primary meta-analyses may contribute to overestimation of effect sizes, particularly where small-study effects were identified. Heterogeneity was also high across meta-analytic findings. This likely reflects genuine variation in effect magnitude across geographic contexts, exposure measurement methods, outcome definitions and population characteristics. Nonetheless, the wide prediction intervals observed for several outcomes indicate that even where pooled effects are convincingly non-null, the true effect in a new population or context may not be significant. Quantitative estimates from this review should therefore be treated as indicative of direction and consistency rather than precise magnitude. We additionally recognise the potential for publication bias across the wider literature.

Finally, we did not report information on environmental mediation which was included in some reviews nor did we include information about the country income status of empirical studies of reviews as this was beyond the scope of this umbrella review. Future umbrella reviews should investigate the effects of environmental mediation factors on outcomes and discuss differential outcomes based on country income status.

## Conclusion

This umbrella review and meta-analysis provides a synthesis of the evidence on the association between urban environment exposures and cognitive health. We found highly probable evidence for the association of air pollution (specifically PM_2.5_, NO_2_, NO_x_) on cognitive health and probable evidence for the association of social environment factors on cognitive health. Further work on urban design features, specifically urban green and blue spaces, would help clarify the existing evidence base. This review indicates the need for policy and practice to focus on reducing levels of air pollution and designing environments which promote neighbourhood quality and walkability to reduce traffic-related exposures. More longitudinal research is required to investigate the impact of urban design features, air pollution (including CO and SO_2_), noise and light exposure, and travel behaviours in relation to cognitive health. Broadening this evidence base may further opportunities to integrate environment factors in clinical recommendations for dementia prevention.

## Supplementary Material

aa-26-0535-File003_afag215
