# Supplementary material for: A meta-analytical umbrella review assessing urban environment exposures and cognitive health outcomes
Source: Age Ageing. 2026 Jul 24;55(7):afag215. doi: 10.1093/ageing/afag215 (PMC13398387; doi:10.1093/ageing/afag215)
Supplement: aa-26-0535-File003_afag215 [file aa-26-0535-file003_afag215.docx]

**A meta-analytical umbrella review assessing urban environment exposures and cognitive health outcomes**

**Supplementary Data**

**Appendix 1 – SPACE Causal Loop Diagram**

**Appendix 2 – Environmental factors in search strategy underpinned by Appendix 1**

**Appendix 3 – Search strategy**

**Appendix 4 – Data extraction**

**Appendix 5 – Meta-analysis methodology**

**Appendix 6 – PRISMA**

**Appendix 7 – Table of exclusions at full text**

**Appendix 8 – JBI risk of bias assessment**

**Appendix 9 – Forest plot**

**Appendix 10 – Forest plot**

**Appendix 11 – Forest plot**

**Appendix 12 – Heat map**

**Appendix 13 – Narrative results**

**Appendix 14 – JBI per environmental exposure**


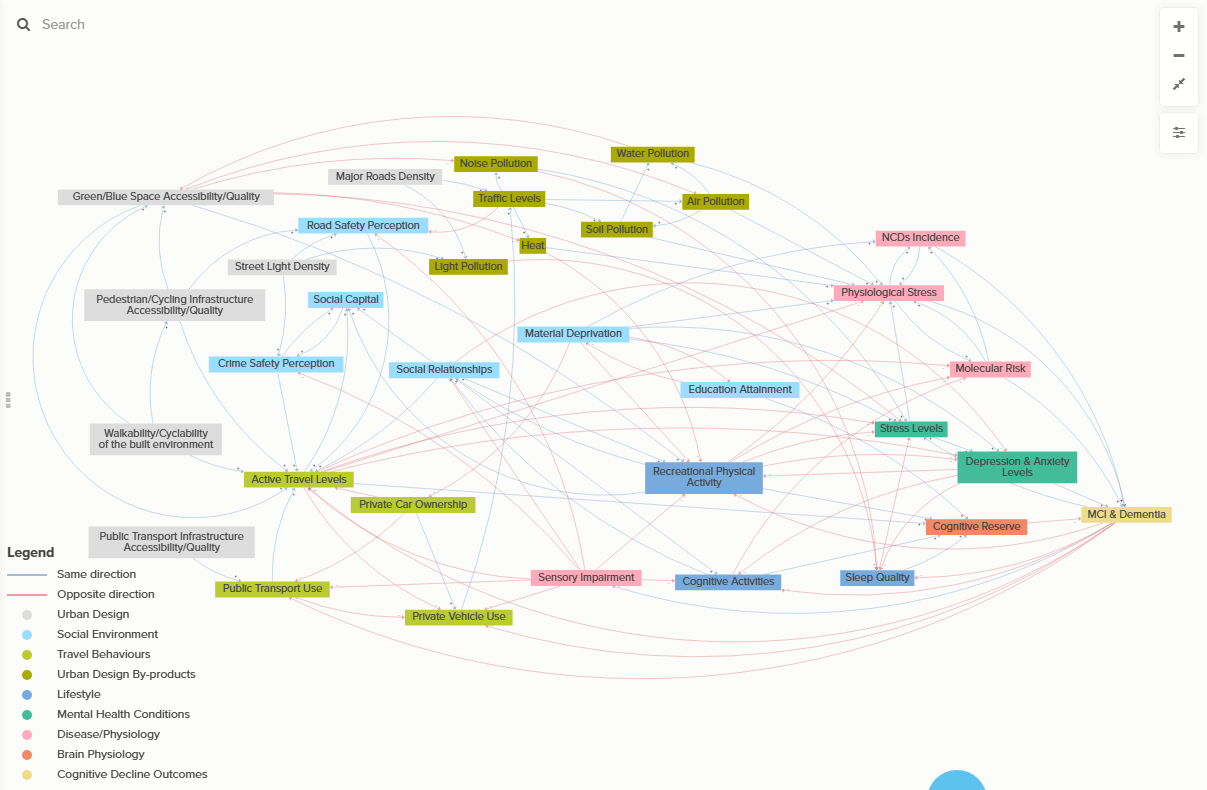


**Appendix 1** – Causal Loop Diagram developed by the SPACE research team (available on kumu) which depicts important directional relationships between the urban environment and dementia

| **Appendix 2 - Environmental factors (descriptors informed by the underpinning CLD framework)** | | | |
| --- | --- | --- | --- |
| **Urban design** | **Urban design by-products** | **Social environment** | **Travel behaviours** |
| Major roads density | Noise pollution | Road safety perception | Active travel levels |
| Green or blue space accessibility/quality | Traffic levels | Social capital | Private car ownership |
| Street light density | Heat | Crime safety perception | Public transport usage |
| Pedestrian/cycling infrastructure | Light pollution | Social relationships | Private vehicle use |
| Walkability/cyclability | Soil pollution | Material deprivation |  |
| Public transport infrastructure | Water pollution |  |  |
|  | Air pollution |  |  |

| **Appendix 3 – Search strategy** | |  |  | |
| --- | --- | --- | --- | --- |
|  | |  | **MOST RECENT SEARCH (January 14th 2025)** | |
| **Broad Term** | | **Search Term** | **Pubmed (filtered for review, systematic review, meta-analysis)*** | **PSYCinfo and Embase (AND review OR systematic review OR meta-analysis OR meta-analyses)**** |
| Dementia, MCI, cog health | 1 | “Mild Cognitive impairment” OR “dementia” OR “Alzheimer*” OR “MCI” OR “cognitive impairment” OR “cognitive health” OR “cognitive decline” | 81,314 | 143,485 |
| Urban design | 2 | “Green space*” OR “blue space” OR UGBS | 429 | 602 |
|  | 3 | walkability OR cyclability OR  “Pedestrian infrastructure” OR “cycling infrastructure” | 236 | 347 |
|  | 4 | “Road density” | 5 | 10 |
|  | 5 | "Street light density" OR "streetlight density" OR street*light | 50 | 12 |
|  |  | 1 AND 2 | 14 | 28 |
|  |  | 1 AND 3 | 3 | 7 |
|  |  | 1 AND 4 | 0 | 0 |
|  |  | 1 AND 5 | 0 | 0 |
| Social environment | 6 | “Social capital” OR “social relationship” | 624 | 1,910 |
|  | 7 | Crime | 16,861 | 13,609 |
|  | 8 | “Material deprivation” OR “deprivation” OR “socio economic status” OR “socioeconomic status” OR “Socio*economic status” OR “Socio*economically disadvantage*” | 16,986 | 35,506 |
|  | 9 | “Educational attainment” OR “educational status” OR “educational level” | 3,912 | 13,986 |
|  |  | 1 AND 6 | 8 | 25 |
|  |  | 1 AND 7 | 183 | 129 |
|  |  | 1 AND 8 | 536 | 1,264 |
|  |  | 1 AND 9 | 269 | 953 |
| Environmental by-products | 10 | “Noise pollution” OR “water pollution” OR “air pollution” OR “soil pollution” OR “light pollution” OR pollutant | 49,934 | 32,377 |
|  | 11 | “Heat stress” OR “ambient temperature” OR heatwaves | 14,589 | 17,968 |
|  |  | 1 AND 10 | 502 | 513 |
|  |  | 1 AND 11 | 57 | 131 |
| Transport behaviours | 12 | "Active travel" OR "active transport" OR walk* OR bicycling OR bike OR biking OR "ecological commut*" OR "ecological transport" OR non-auto* OR non-motori?e* OR "green travel" OR "green transport" | 320,017 | 37,253 |
|  | 13 | car use OR car usage OR car dependency OR car ownership | 8,366 | 89 |
|  | 14 | cycle lane OR bicycle lane OR bike lane OR cycle trail OR bicycle trail OR cycle path OR bicycle path OR bike path OR bike*way OR foot*path OR pavement OR sidewalk OR greenway OR walkability OR cyclability OR  “Pedestrian infrastructure” OR “cycling infrastructure” | 1,333 | 642 |
|  | 15 | “Public transport” OR “public transit” | 172 | 350 |
|  | 16 | traffic | 9226 | 2,2042 |
|  |  | 1 AND 12 | 715 | 1,418 |
|  |  | 1 AND 13 | 82 | 0 |
|  |  | 1 AND 14 | 17 | 9 |
|  |  | 1 AND 15 | 5 | 3 |
|  |  | 1 AND 16 | 232 | 510 |
| *Pubmed reviews were gathered by adding the ‘review,’ ‘systematic review,’ and ‘meta-analysis’ filters.  **Embase and PSYCinfo reviews were gathered by adding ‘AND (review or systematic review OR meta-analysis OR meta-analyses)’ at the end of every search string.  Cog health: cognitive health; MCI: Mild cognitive impairment; UGBS: Urban green and blue space. | | | | |

**Appendix 4 - Data extraction**

Data extracted included: environmental exposure type; outcome type; number of studies per outcome; number of participants per outcome; age category classified by authors as children (<18 years old), adult (18-65 years old), adults and older adults or older adult (>65 years old) based on if the outcome had the majority of studies reporting results of one of these age categories; number of studies reporting significant results; design of empirical studies (cross-sectional, cohort/longitudinal, case-control or mixed if reporting results from studies of different designs); and a summary sentence relating to the specific outcome of interest. Where possible, authors synthesised information manually when not specified by reviews such as sample size, number of studies, and number of studies reporting significant information. In systematic reviews which provided detailed information on empirical study outcomes, it was possible to categorise the number of studies reporting significant information. Authors categorised the number of ‘positive’, ‘negative’ and ‘mixed’ outcomes based on studies reporting a positive, negative or mixed effect on cognitive health. The mixed classification was used in the instance of conflicting study findings (e.g., an association between a cognitive outcome and air pollution within one buffer to residence (200m) but not another buffer (500m) or an association between one aspect/domain of a cognitive test such as psychomotor speed but not all aspects/domains). In the instance of missing data or being unable to generate data due to insufficient explanation and reporting, we were unable to report findings from studies which is stated as NR (not reported) in the following tables.

| \| **Appendix 4 Table 1. Data extraction - Urban design outcomes** \| \| \| \| \| \| \| \| \| \| \| --- \| --- \| --- \| --- \| --- \| --- \| --- \| --- \| --- \| --- \| \| **First author (year of publication)** \| **Environmental exposure** \| **Outcome(s)** \| **Study design(s)** \| **Total included studies** \| ***Studies reporting statistically significant results n (%)** \| **Total participants** \| **Age category** \| **JBI critical appraisal quality score** \| **Summary of results** \| \| **Roads, traffic density, or low emission zones** \| \| \| \| \| \| \| \| \| \| \| **Chamberlain (2023)**[1] \| Low emission zones \| Dementia \| NR \| 1 \| 0 (0.0) \| NR \| Older adults \| Moderate \| Low emission zones were not significantly associated with dementia diagnosis in hospital \| \| **Chen (2022)**[2] \| Living in proximity to major roads \| Cognitive performance \| Mixed \| 3 \| 3 (100.0) \| NR \| Older adults \| Moderate \| Three studies demonstrated the detrimental effects of living closer to major roads on cognitive performance in later life \| \| **Clifford (2016)**[3] \| In utero proximity to traffic density \| Cognitive function \| Cohort/Longitudinal \| 1 \| 1 mixed \| 1,109 \| Children \| Moderate \| Near residence traffic density at birth was associated with higher scores for non-verbal intelligence, design memory, and picture memory but not verbal intelligence or visual motor skills \| \| **Da (2024)**[4] \| Proximity to major roads \| Dementia \| Cohort/Longitudinal \| 3 \| 3 (100.0) \| 2,728,744 \| Older adults \| High \| In meta-analysis random effects models RR 1.09 95% CI 0.94-1.27 \| \| **Dimakakou (2018)**[5] \| Proximity to a very busy road \| Cognitive function \| Cohort/Longitudinal \| 1 \| 1 (100.0) \| 402 \| Older adults \| Moderate \| One study supported an association between shorter distance to a very busy road (10,000 cars per day) and poorer performance in a general assessment of cognition \| \| **Peters (2019)**[6] \| Distance to road \| Incidence of dementia \| Cohort/Longitudinal \| 2 \| 1 mixed \| 246,478 \| Older adults \| Moderate \| One study found less distance to major roadways was associated with increased incidence of dementia except for the 200-300m buffer \| \| **Song (2024)**[7] \| Distance from homes to major roads \| Cognitive function, cognitive impairment, or dementia \| Mixed \| NR \| NR \| NR \| Older adults \| Moderate \| Distance to major roads was negatively associated with cognition \| \| **Zhao (2021)**[8] \| Road proximity \| Cognitive impairment and dementia \| Mixed \| 5 \| NR \| 5,088,169 \| Adults and older adults \| Moderate \| Significant associations were found between proximity to roads and cognitive impairment and dementia in all analyses OR 1.08 95% CI 1.04-1.12 and OR 1.07 95% CI 1.03-1.11 \| \| **Green and/or blue space** \| \| \| \| \| \| \| \| \| \| \| **Chen (2022)**[2] \| Greenness (NDVI) \| Cognitive performance \| Mixed \| 6 \| 6 (100.0) \| NR \| Older adults \| Moderate \| Six studies consistently demonstrated the cognitive advantages of living in a neighbourhood with more greenspace measured by NDVI \| \|  \| Park availability \| Cognitive performance \| Mixed \| 2 \| 2 (100.0) \| NR \| Older adults \| Moderate \| Two life course studies indicated that early-life (childhood and adulthood) park availability was associated with better cognitive ageing in later life \| \|  \| Greenness \| Cognitive performance \| Mixed \| 4 \| 2 (50.0) \| NR \| Older adults \| Moderate \| Two studies reported that higher exposure to greenspace was related to higher odds of cognitive impairment or dementia, and another two found the relationships between green space/park exposure and cognition/dementia to be insignificant \| \| **Da (2024)**[4] \| Green and blue space \| Dementia \| NR \| 5 \| 5 (100.0) \| NR \| Older adults \| High \| In meta-analysis random effects model RR 0.94 95% CI 0.92-0.96 \| \| **de Keijzer (2016)**[9] \| Green space \| Cognition \| Mixed \| 6 \| NR \| 4,194 \| Children \| Moderate \| Studies suggest green space exposure is beneficial to children’s cognition \| \|  \| Green space \| Cognition \| Cross-sectional \| 3 \| NR \| 2,872 \| Adults \| Moderate \| Studies suggest green space exposure is beneficial to adults cognition \| \|  \| Green space \| Cognition \| Mixed \| 4 \| NR \| 7,062 \| Older adults \| Moderate \| Som studies suggest green space exposure is beneficial or protective to older adults cognition \| \| **Michael (2024)**[10] \| Green and blue space \| Cognitive function, cognitive decline, or dementia \| Cohort/Longitudinal \| 10 \| 3 + 5 mixed \| NR \| Older adults \| Moderate \| Half of the studies had mixed findings, some studies found that green or blue space was associated with cognition \| \| **Song (2024)**[7] \| Blue and green space \| Cognitive function, cognitive impairment, or dementia \| Mixed \| NR \| NR \| NR \| Older adults \| Moderate \| Green space was seen to have a positive association with cognition, blue space was less researched \| \| **Wang (2024)**[11] \| Percentage of blue space \| Cognitive function \| Cross-sectional \| 1 \| 1 (100.0) \| 4,141 \| Adults \| Moderate \| Blue space has positive indirect effects on cognitive function through waist circumference and glycated haemoglobin \| \|  \| Percentage of blue space \| Alzheimer’s disease and Alzheimer’s disease related dementia \| Cohort/Longitudinal \| 1 \| 0 (0.0) \| 123,335,839 \| Older adults \| Moderate \| No significant findings for Alzheimer’s disease and Alzheimer’s disease related dementia \| \| **Zagnoli (2022)**[12] \| Greenness (NDVI) \| Dementia \| Cross-sectional \| 2 \| 2 (1000) \| 478,005 \| Older adults \| Moderate \| No significant association between NDVI and dementia was found in pooled analysis \| \|  \| Greenness (NDVI) \| Dementia \| Cohort/Longitudinal \| 5 \| 3 (60.0) \| 2,632,768 \| Older adults \| Moderate \| No significant association between NDVI and dementia was found in pooled analysis \| \| **Zhang (2024)**[13] \| Green space (NDVI or area of green space) \| Dementia \| Cohort/Longitudinal \| 9 \| 6 (66.7) \| 65,399,147 \| Older adults \| High \| In random effects model, green space was seen as protective of dementia OR 0.95 (95% CI 0.93-0.96) \| \| **Zhao (2021)**[8] \| Greenness \| Cognitive impairment and dementia \| Mixed \| 8 \| NR \| 2,521,214 \| Adults and older adults \| Moderate \| Meta-analysis yielded a non-significant relationship between more greenness and cognitive impairment OR 0.97 95% CI 0.95-1.00 \| \|  \| Greenness \| Cognitive impairment and dementia \| Cohort/Longitudinal \| 4 \| NR \| 2,483,442 \| Adults and older adults \| Moderate \| Greenness was found to be a protective factor for cognitive impairment and dementia OR 0.96 9%C CI 0.95-0.98 \| \| **Public transport infrastructure** \| \| \| \| \| \| \| \| \| \| \| **Chen (2022)**[2] \| Public transport \| Cognitive performance \| Mixed \| 2 \| 2 (100.0) \| NR \| Older adults \| Moderate \| Two studies found that residing in neighbourhoods with accessible public transit was associated with slower cognitive decline over time \| \| **Michael (2024)**[10] \| Public transport \| Cognitive function, cognitive decline, and dementia \| Cohort/Longitudinal \| 2 \| 1 mixed \| NR \| Older adults \| Moderate \| One study had mixed findings regarding the association between public transport and cognition \| \| **Song (2024)**[7] \| Public transportation stops \| Cognitive function, cognitive impairment, or dementia \| Mixed \| NR \| NR \| NR \| Older adults \| Moderate \| Number of public transport stops was associated with positively associated with cognition \| \| **Walkability or cyclability related infrastructure** \| \| \| \| \| \| \| \| \| \| \| **Chen (2022)**[2] \| Walkability \| Cognitive performance \| Mixed \| 3 \| 3 (100.0) \| NR \| Older adults \| Moderate \| Three studies highlighted the positive effects of living in a walkable neighbourhood on cognitive functioning in later life \| \|  \| Street connectivity \| Cognitive performance \| Mixed \| 2 \| 2 (100.0) \| NR \| Older adults \| Moderate \| Two studies reported a positive association between objectively measured street connectivity and global cognition \| \|  \| Aesthetic and pleasant neighbourhoods \| Cognitive performance \| Mixed \| 2 \| 2 (100.0) \| NR \| Older adults \| Moderate \| Two studies reported positive effects of an aesthetic and pleasant neighbourhood on the cognitive performance at older age \| \|  \| Infrastructure for walking and cycling \| Cognitive performance \| Mixed \| 2 \| 0 (0.0) \| NR \| Older adults \| Moderate \| No association between infrastructure for walking or cycling and cognition/dementia \| \|  \| Road tidiness \| Cognitive performance \| NR \| 1 \| 0 (0.0) \| NR \| Older adults \| Moderate \| No association between road tidiness and cognition/dementia \| \|  \| Handicapped access \| Cognitive performance \| NR \| 1 \| 0 (0.0) \| NR \| Older adults \| Moderate \| No association between handicapped access and cognition/dementia \| \| **Michael (2024)**[10] \| Walkability \| Cognitive function, cognitive decline, or dementia \| Cohort/Longitudinal \| 2 \| 1 + 1 mixed \| NR \| Older adults \| Moderate \| One study found an association between walkability and cognition while the other study had mixed results \| \| **Song (2024)**[7] \| Neighbourhood built environment design (street connectivity, street integration, street layout, and walkability) \| Cognitive function, cognitive impairment, or dementia \| Mixed \| NR \| NR \| NR \| Older adults \| Moderate \| Most studies found a significant positive association between cognitive function and street connectivity or walkability but there is potentially a negative relationship between cognition and neighbourhood integration \| \| **Other urban design features** \| \| \| \| \| \| \| \| \| \| \| **Chen (2022)**[2] \| Neighbourhood resources \| Cognitive performance \| Mixed \| 8 \| NR \| NR \| Older adults \| Moderate \| The relationship between neighbourhood resource availability and cognition/dementia were mixed. \| \|  \| Neighbourhood physical disorder \| Cognitive performance \| Mixed \| 5 \| 3 (60.0) \| NR \| Older adults \| Moderate \| Three studies found perceived neighbourhood physical disorder was a significant predictor of worse cognitive performance in older age. However, two studies using objective composite measures of neighbourhood physical disorder did not find significant associations with cognition. These studies suggested that older adults’ perceptions of the neighbourhood physical disorder matter and may have more direct influence on cognitive performance outcomes. \| \| **Michael (2024)**[10] \| Neighbourhood quality/aesthetics/disorder \| Cognitive function, cognitive decline, or dementia \| Cohort/Longitudinal \| 5 \| 3 + 2 mixed \| NR \| Older adults \| Moderate \| Some studies found that neighbourhood quality/aesthetics/disorder was associated with cognition, other studies had conflicting findings \| \|  \| Infrastructure \| Cognitive function, cognitive decline, or dementia \| Cohort/Longitudinal \| 1 \| 1 mixed \| NR \| Older adults \| Moderate \| One study had mixed results regarding the association between infrastructure and cognition \| \| **Song (2024)**[7] \| Neighbourhood built environment diversity (land use mix and recreation centres etc) \| Cognitive function, cognitive impairment, or dementia \| Mixed \| NR \| NR \| NR \| Older adults \| Moderate \| Most studies suggest higher land use mix and range of neighbourhood resources may have a positive association with cognition \| \|  \| Neighbourhood built environment quality (street disrepair index, cleanliness, neighbourhood physical disorder) \| Cognitive function, cognitive impairment, or dementia \| Mixed \| NR \| NR \| NR \| Older adults \| Moderate \| Higher quality of the neighbourhood built environment may have a positive association with cognition \| \| **Zagnoli (2022)**[12] \| Land use/Land cover \| Cognitive impairment \| Cross-sectional \| 2 \| 2 (100.0) \| 9,929 \| Older adults \| Moderate \| No significant effect was found between NDVI and cognitive impairment in pooled analysis \| \|  \| Land use/Land cover \| Dementia \| Cross-sectional \| 3 \| 1 (33.3) \| 34,731 \| Older adults \| Moderate \| No significant association was found between land use/land cover and dementia in pooled analysis \| \|  \| Land use/Land cover \| Dementia \| Cohort/Longitudinal \| 2 \| 0 (0.0) \| 354,502 \| Older adults \| Moderate \| Statistically significant effect for the association between green space quantity and dementia prevalence (RR 1.47 95%CI 1.22-1.76) \| \| CI=Confidence Interval; CO=Carbon Monoxide; db=decibel; HR=Hazard Ratio; JBI= Joanna Briggs Institute; L_DEN_=Noise level over the day; L_NIGHT_=Noise level over the night ;m^3^=meters cubed; MCI=Mild Cognitive Impairment; NDVI= Normalised Difference Vegetation Index; NO_2_=Nitrogen Dioxide; NO_x_=Nitrogen Oxides; NR=Not Reported due to insufficient data for curation or missing data;O_3_=Ozone; OR= Odds Ratio; PM/PM_x_=Particulate Matter of non-descript size;PM_10_=Particulate Matter less than 10 micrometers in diameter;PM_2.5_= Particulate Matter less than 2.5 micrometers in diameter; ppb=parts per billion; RR=Risk Ratio;SO_2_=Sulfur Dioxide; Tn (e.g., T1)=Tertile 1;ug=microgram; UK= United Kingdom; USA= Unites States of America; vs=versus \| \| \| \| \| \| \| \| \| \| \| *percentage not reported for reviews which had any mixed results \| \| \| \| \| \| \| \| \| \|          \| **Appendix 4 Table 2. Data extraction - Urban design by-products outcomes** \| \| \| \| \| \| \| \| \| \| \| --- \| --- \| --- \| --- \| --- \| --- \| --- \| --- \| --- \| --- \| \| **First author (year of publication)** \| **Environmental exposure** \| **Outcome(s)** \| **Study design(s)** \| **Total included studies** \| ***Studies reporting statistically significant results n (%)** \| **Total participants** \| **Age category** \| **JBI critical appraisal quality score** \| **Summary of results** \| \| **Air pollution – Overall air pollution** \| \| \| \| \| \| \| \| \| \| \| **Bejot (2018)**[14] \| Air pollution \| Cognitive decline \| Mixed \| 9 \| 9 (100.0) \| NR \| Adults and older adults \| Moderate \| Strong evidence of the association of cognitive decline with PM_2.5_, PM_10_, O_3_, and NO_2_ exposure \| \|  \| Air pollution \| Alzheimer’s disease \| Mixed \| 5 \| 5 (100.0) \| NR \| Adults and older adults \| Moderate \| There is evidence of the association of Alzheimer’s disease occurrence with O_3_, PM_2.5_, PM_10_, NO_2_ and NO_X_ exposure \| \|  \| Air pollution \| Vascular dementia \| Mixed \| 4 \| 4(100.0) \| NR \| Adults and older adults \| Moderate \| There is evidence of association of vascular dementia with O_3_, NO_2_, and NO_X_ exposure \| \| **Chandra (2022)**[15] \| Prenatal exposure to multiple air pollutants \| Cognitive and developmental indicators \| Mixed \| 2 \| 2 (100.0) \| NR \| Children \| High \| Exposure to multiple air pollutants in the prenatal period and infancy (until 1 year of age) had an adverse impact on psychomotor development and cognitive performance, adaptive functioning, and behavioural indices \| \|  \| Air pollution \| Cognitive performance \| Mixed \| 3 \| 2 (66.7) \| NR \| Adults \| High \| Two studies from the USA and China reported impaired cognitive performance associated with exposure to air pollutants on cognitive performance in adults, which was contradicted by another study from the USA \| \| **Clifford (2016)**[3] \| Postnatal air pollution \| Cognitive function \| Cross-sectional \| NR \| NR \| NR \| Children \| Moderate \| Findings suggestive of a relationship between air pollution exposure and poorer cognitive performance \| \|  \| Air pollution \| Cognitive function \| Cross-sectional \| 1 \| Mixed \| NR \| Adults \| Moderate \| PM_10_ had little association with cognitive functions after adjustment while ozone was associated with significantly poorer visual-motor coordination and learning \| \|  \| Air pollution \| Cognitive decline \| Mixed \| 12 \| Mixed \| NR \| Older adults \| Moderate \| Studies of older adults showed varying and conflicting results \| \| **Da (2025)**[4] \| Multiple air pollutants \| Cognitive function or dementia \| Mixed \| 30 \| NR \| 81,559,340 \| Older adults \| High \| Strong associations were particularly found between long term PM_2.5_ exposure and dementia \| \| **Fu (2020)**[16] \| Air pollution (total effect size) \| Alzheimer’s disease \| Cohort/Longitudinal \| 14 \| 12 (85.7) \| 12,614,523 \| Older adults \| Moderate \| Overall air pollution levels significantly associated with Alzheimer’s disease as they reported an overall OR 1.32 95% CI 1.09-1.61 in random-effect meta-analysis \| \| **Lu (2020)**[17] \| Air pollution \| Cognitive functioning \| Mixed \| NR \| NR \| NR \| All age categories \| Low \| Air pollution harms cognitive functioning across all life stages, from prenatal development, childhood, and youth to young and old adults \| \| **Oliveira (2024)**[18] \| Various air pollutants \| Dementia \| NR \| 1 \| 1 \| NR \| Adults \| Moderate \| Significant associations between all air pollutants and dementia \| \| **Peters (2015)**[19] \| Air pollution \| Prevalence cognitive impairment \| Cohort/ Longitudinal \| 6 \| 3 + 3 mixed \| 31,129 \| Older adults \| Moderate \| Half of the studies had mixed results as air pollution was associated with one aspect of a cognitive test but not all \| \|  \| Air pollution \| Incidence of cognitive impairment \| Cross-sectional \| 2 \| 2 mixed \| 49,648 \| Older adults \| Moderate \| Both studies reported mixed findings as air pollution was associated with worse cognition in some analyses and not others \| \| **Tang (2023)**[20] \| Air pollution \| Vascular dementia \| Cohort/Longitudinal \| 3 \| 1 (33.3) \| 483,628 \| Adults \| Moderate \| No significant association between overall air pollution and vascular dementia \| \|  \| Air pollution \| Dementia \| Cohort/Longitudinal \| 12 \| 9 (75.0) \| 13,284,070 \| Adults \| Moderate \| Significant association between air pollutants and risk ratio of dementia \| \| **Tzivian (2015)**[21] \| Air pollution \| Cognitive performance \| Mixed \| 10 \| NR \| NR \| Older adults \| Moderate \| Several studies reported associations between cognitive performance and air pollution, however conclusions are very difficult to assess due to very large heterogeneity \| \|  \| Air pollution \| Alzheimer’s like pathology \| Mixed \| 2 \| 2 (100.0) \| 106 \| Adults \| Moderate \| Higher exposure of air pollution was associated with Alzheimer’s like pathology \| \| **Air pollution - PM_2.5_** \| \| \| \| \| \| \| \| \| \| \| **Abolhasani (2023)**[22] \| PM_2.5_ \| Dementia \| Mixed \| 8 \| 3 (37.5) \| 4,110,289 \| Older adults \| High \| Hazard Ratio of dementia per 1μg/m3 increase in PM_2.5_ levels was significant HR 1.02 95% CI 1.01-1.03 however, the effect size was very small, and the prediction interval was not significant \| \|  \| PM_2.5_ \| Alzheimer’s disease \| NR \| 1 \| 0 (0.0) \| 95,690 \| Older adults \| High \| Hazard Ratio of Alzheimer’s disease per 1μg/m^3^ increase in PM_2.5_ levels was not significant. HR 1.00 95% CI 1.00-1.01 \| \|  \| PM_2.5_ \| Non-Alzheimer’s disease dementia \| NR \| 1 \| 0 (0.0) \| 633,949 \| Older adults \| High \| Hazard Ratio of non-Alzheimer’s disease per 1μg/m^3^ increase in PM_2.5_ levels was not significant HR 1.01 95% CI 0.99-1.03 \| \| **Cheng (2022)**[23] \| PM_2.5_ \| Vascular dementia \| Cohort/Longitudinal \| 8 \| 4 (50.0) \| 14,050,098 \| Older adults \| Moderate \| Significant associations were found between PM_2.5_ exposure and vascular dementia HR 2.0 95% CI 1.3-3.08, dementia HR 1.40, 95% CI 1.23,1.60, and Alzheimer’s disease HR 1.47 95% CI 1.22-1.78 in random-effect models \| \|  \| PM_2.5_ \| Dementia \| Cohort/Longitudinal \| 17 \| 14 (82.4) \| 40,218,410 \| Older adults \| Moderate \| \|  \| PM_2.5_ \| Alzheimer’s disease \| Cohort/Longitudinal \| 12 \| 7 (58.3) \| 1,656,291 \| Older adults \| Moderate \| \| **Clifford (2016)**[3] \| In utero PM_2.5_ \| Cognitive function \| Cohort/Longitudinal \| 3 \| 1 (33.3) \| 11,029 \| Children \| Moderate \| One of three studies found that in utero PM_2.5_exposure was associated with reduced mental score \| \| **Cristaldi (2022)**[24] \| PM_2.5_ \| Alzheimer’s disease \| Mixed \| 8 \| NR \| NR \| Children, adults, and older adults \| Low \| Positive association was found even after short term exposure to PM_2.5_ and the onset of Alzheimer’s disease. Furthermore, the association between PM_2.5_ exposure and Alzheimer’s disease also seems to depend on genetic and physiological factors. Overall, the hazard ratios reported in the selected studies were not particularly high (from 1.008 to 1.13), but they showed an association between the PM_2.5_ exposure and Alzheimer’s disease onset \| \|  \| PM_2.5_ \| Vascular dementia \| Cohort/ Longitudinal \| 3 \| 2 (66.7) \| NR \| Older adults \| Low \| Authors report a positive association between exposure to PM_2.5_ and the onset of Vascular dementia. \| \| **Dhiman (2022)**[25] \| PM_2.5_ \| All-cause dementia \| Mixed \| 8 \| 4 (50.0) \| 15,902,735 \| Older adults \| Moderate \| The pooled hazard ratio for PM_2.5_ and all cause dementia was significant HR 1.03 95% CI 1.01-1.06 \| \|  \| PM_2.5_ \| Alzheimer’s disease \| Mixed \| 6 \| 3 (50.0) \| 1,915,318 \| Older adults \| Moderate \| The pooled hazard ratio for PM_2.5_ and Alzheimer’s disease was significant HR 1.08 95% CI 1.01-1.15. \| \|  \| PM_2.5_ \| Vascular dementia \| Mixed \| 4 \| 1 (25.0) \| 13,676,529 \| Older adults \| Moderate \| The pooled hazard ration for PM_2.5_ and vascular dementia was not significant HR 1.03 95% CI 1.00-1.06 \| \|  \| PM_2.5_ \| Cognitive decline \| Mixed \| 12 \| 12 (100.0) \| 134,441 \| Older adults \| Moderate \| All studies should a positive association of exposure to PM_2.5_ and cognitive decline \| \| **Fu (2019)**[26] \| PM_2.5_ \| Mild cognitive impairment \| Mixed \| 9 \| 3 (33.3) \| 75,957 \| Older adults \| Low \| Meta-analysis did not show significance OR 0.98 95%CI 0.96-1.01 \| \|  \| PM_2.5_ \| Dementia \| Cohort/ Longitudinal \| 3 \| 3 (100.0) \| 1,105,245 \| Older adults \| Low \| Meta-analysis showed a significant relationship between PM_2.5_ and dementia OR 1.16 95% CI 1.07-1.26 \| \|  \| PM_2.5_ \| Alzheimer’s disease \| Mixed \| 3 \| 3 (100.0) \| 686,486 \| Older adults \| Low \| Meta-analysis did not show significance OR 3.26 95% CI 0.84-12.74 \| \| **Gong (2023)**[27] \| PM_2.5_ \| All-cause dementia \| Mixed \| 17 \| 12 (70.6) \| 110,246,807 \| Adults and older adults \| High \| In meta-analysis, random effects model, the pooled effect for 10 μg/m^3^ growth in exposure to PM_2.5_OR 1.3 95% CI 1.14-1.47 \| \|  \| PM_2.5_ \| Alzheimer’s disease \| Mixed \| 13 \| 7 (53.9) \| 168,414,337 \| Adults and older adults \| High \| In meta-analysis random effects model, the pooled effect for 10 μg/m^3^ growth in exposure to PM_2.5_OR 1.65 95% CI 1.37-1.94 \| \|  \| PM_2.5_ \| Vascular dementia \| Mixed \| 8 \| 4 (50.0) \| 95,073,920 \| Adults and older adults \| High \| In meta-analysis random effects model, the pooled effect for 10 μg/m^3^ growth in exposure to PM_2.5_OR 1.45 95% CI 0.84-2.06 \| \|  \| PM_2.5_ \| Cognitive function (MMSE) \| Mixed \| 6 \| 2 (33.3) \| 20,866 \| Adults and older adults \| High \| In meta-analysis random effects model, the pooled effect for 10 μg/m^3^ growth in exposure to PM_2.5_OR -0.1 95% CI -0.18- -0.02 \| \|  \| PM_2.5_ \| Cognitive function (neuropsychological tests) \| Mixed \| 5 \| 3 (60.0) \| 75,036 \| Adults and older adults \| High \| In meta-analysis random effects model, the pooled effect for 10 μg/m^3^ growth in exposure to PM_2.5_OR -0.19 95% CI -0.33- -0.06 \| \|  \| PM_2.5_ \| Cognitive function (TICS) \| Mixed \| 3 \| 1 (33.3) \| 58,058 \| Adults and older adults \| High \| In meta-analysis random effects model, the pooled effect for 10 μg/m^3^ growth in exposure to PM_2.5_OR -0.00 95% CI -0.21-0.2 \| \| **Meo (2024)**[28] \| PM_2.5_ \| Cognitive impairment \| Cohort/ Longitudinal \| 10 \| 7 (70.0) \| 548,968 \| Older adults \| Moderate \| In a random effect model, PM_2.5_ was associated with a significant increased risk of cognitive impairment (OR1.35 95% CI 1.02–1.79) \| \|  \| PM_2.5_ \| Cognitive impairment \| Cross-sectional \| 7 \| 5 (71.4) \| 26,962 \| Older adults \| Moderate \| In a random effect models, PM_2.5_ was associated with a non-significant increased risk of cognitive impairment (OR 2.66, 95%  CI 0.79–8.90) \| \| **Mohammadzadeh (2024)**[29] \| PM_2.5_ \| Alzheimer’s dementia \| Mixed \| 24 \| NR \| 206,210,080 \| Older adults \| Moderate \| The majority of studies found PM_2.5_ exposure increased risk of Alzheimer’s dementia \| \|  \| PM_2.5_ \| Non-Alzheimer’s dementia \| Mixed \| 33 \| NR \| 148,596,435 \| Older adults \| Moderate \| The overwhelming majority of studies found that exposure to PM_2.5_ was associated with increased risk of non-Alzheimer’s dementia \| \| **Oliveira (2024)**[18] \| PM_2.5_ \| Dementia (including Alzheimer’s disease) \| Mixed \| 18 \| 13 (72.2) \| NR \| Adults \| Moderate \| Majority of studies found that higher air pollution exposure was significantly associated with risk of dementia \| \| **Peters (2019)**[6] \| PM_2.5_ \| Cognitive decline \| Cohort/Longitudinal \| 5 \| 3 mixed \| 82,482 \| Older adults \| Moderate \| Half of the studies showed a significant association between PM_2.5_and cognitive decline, with all outcomes reporting conflicting findings \| \|  \| PM_2.5_ \| Incidence of dementia \| Cohort/Longitudinal \| 4 \| 3 (75.0) \| 2,295,113 \| Older adults \| Moderate \| The majority of studies showed a significant association between PM_2.5_ exposure and incidence of dementia \| \| **Peters (2021)**[30] \| PM_2.5_ \| Cognitive decline \| Mixed \| 7 \| 3 (42.9) \| NR \| Older adults \| Moderate \| The majority of studies on cognitive decline were not significant \| \|  \| PM_2.5_ \| Incidence of dementia \| Mixed \| 7 \| 5 (71.4) \| NR \| Older adults \| Moderate \| Most of the studies on incidence of dementia demonstrated an increased risk with higher PM_2.5_ concentrations \| \| **Power (2016)**[31] \| PM_2.5_ \| Incidence of cognitive impairment \| Mixed \| 2 \| 0 (0.0) \| NR \| Adults and older adults \| Moderate \| Neither study of PM_2.5_ and incidence of cognitive impairment found support for an adverse association \| \|  \| PM_2.5_ \| Cognitive level or cognitive decline \| Mixed \| 6 \| Mixed \| NR \| Adults and older adults \| Moderate \| The results were mixed with the majority of studies finding an adverse association with one cognitive task but not others \| \| **Tang (2022)**[20] \| PM_2.5_ T1 vs T2 \| Dementia \| Cohort/Longitudinal \| 11 \| 6 (54.6) \| 13,155,894 \| Adults \| Moderate \| Significant dose response relationship between PM_2.5_ and dementia in both T1 vs T2 and T1 vs T3 \| \|  \| PM_2.5_ T1 vs T3 \| Dementia \| Cohort/Longitudinal \| 11 \| 9 (81.8) \| 13,155,894 \| Adults \| Moderate \| \| **Tsai (2019)**[32] \| PM_2.5_ \| Dementia \| Mixed \| 4 \| 4 (100.0) \| 12,119,853 \| Older adults \| Moderate \| Significant risk PM_2.5_ exposure and dementia \| \|  \| PM_2.5_ \| Alzheimer’s disease \| Mixed \| 3 \| 3 (100.0) \| 10,053,214 \| Older adults \| Moderate \| Significant risk PM_2.5_ exposure and Alzheimer’s disease \| \| **Weuve (2021)**[33] \| PM_2.5_ \| Cognitive performance \| Mixed \| NR \| NR \| NR \| Adults and older adults \| Moderate \| Half of the studies on PM_2.5_ and cognitive level suggested an adverse association \| \| **Xu (2024)**[34] \| PM_2.5_ \| Decreased cognitive function \| Cohort/ Longitudinal \| 7 \| 7 (100.0) \| 89,644 \| NR \| Moderate \| Combined effect of RR 1.17 (95% CI 1.09-1,26) in meta-analysis \| \|  \| PM_2.5_ \| Cognitive test scores \| Cohort/ Longitudinal \| 8 \| 6 (75.0) \| 42,350 \| NR \| Moderate \| Combined effect of PM_2.5_was associated with a 0.3 point decrease in scores (95% CI -0.43- -0.16) \| \| **Yang (2022)**[35] \| PM_2.5_ \| Alzheimer’s disease \| Cohort/Longitudinal \| 1 \| 0 (0.0) \| 95,690 \| Older adults \| Moderate \| No significant associations were found between PM_2.5_ exposure and Alzheimer’s in elderly people in Asia pacific cohort study \| \|  \| PM_2.5_ \| Cognitive impairment \| Cohort/Longitudinal \| 2 \| 2 (100.0) \| 13,684 \| Older adults \| Moderate \| Both studies show sig associations between PM_2.5_ exposure and cognitive impairment in Asia Pacific cohort studies \| \| **Yu (2020)**[36] \| PM_2.5_ (per 5ug/m3 increment) \| Cognitive impairment \| Cohort/Longitudinal \| 11 \| 5 (45.5) \| 12,656,337 \| Older adults \| High \| No significant association was found in combined analysis \| \| **Yuan (2024)**[37] \| PM_2.5_ \| Cognitive function \| Cohort/Longitudinal \| 2 \| 1 (50.0) \| 11,905 \| Children \| Moderate \| One study found a significant association between higher PM_2.5_ exposure and lower cognitive function, the other study did not find any significant associations. \| \|  \| PM_2.5_ \| Cognitive function \| Cohort/Longitudinal \| 3 \| 2 (66.7) \| 9,092 \| Adults and older adults \| Moderate \| The majority of studies found that air pollution significantly negatively impacted cognitive function \| \| **Zhao (2021)**[8] \| PM_2.5_ \| Alzheimer’s disease \| Mixed \| 6 \| NR \| 10,461,733 \| Adults and older adults \| Moderate \| No significant association was found between PM_2.5_ exposure and Alzheimer’s disease \| \|  \| PM_2.5_ \| Vascular dementia \| Cohort/ Longitudinal \| 4 \| NR \| 548,237 \| Adults and older adults \| Moderate \| A significant association was found between PM_2.5_ exposure and vascular dementia \| \| **Air pollution - PM_10_** \| \| \| \| \| \| \| \| \| \| \| **Clifford (2016)**[3] \| In utero PM_10_ \| Cognitive function \| Cohort/Longitudinal \| 1 \| 0 (0.0) \| 9,482 \| Children \| High \| No significant findings \| \| **Gong (2023)**[27] \| PM_10_ \| All-cause dementia \| Mixed \| 4 \| 2 (50.0) \| 1,116,856 \| Adults and older adults \| High \| In meta-analysis random effects model, the pooled effect for 10 μg/m^3^ growth in exposure to PM_10_OR 1.23 95% CI 0.93-1.52 \| \|  \| PM_10_ \| Alzheimer’s disease \| Mixed \| 5 \| 2 (40.0) \| 767,453 \| Adults and older adults \| High \| In meta-analysis random effects model, the pooled effect for 10 μg/m^3^ growth in exposure to PM_10_OR 1.21 95% CI 0.87-1.55 \| \|  \| PM_10_ \| Vascular dementia \| Mixed \| 4 \| 2 (50.0) \| 766,749 \| Adults and older adults \| High \| In meta-analysis fixed effects model, the pooled effect for 10 μg/m3 growth in exposure to PM_10_ OR 1.12 95% CI 1.04-1.21 \| \|  \| PM_10_ \| Cognitive function (MMSE) \| Mixed \| 2 \| 0 (0.0) \| 1,843 \| Adults and older adults \| High \| In meta-analysis fixed effects model, the pooled effect for 10 μg/m3 growth in exposure to PM_10_ OR -0.05 95%CI -0.14-0.03 \| \|  \| PM_10_ \| Cognitive function (Neuropsychological tests) \| Mixed \| 4 \| 2 (50.0) \| 12,480 \| Adults and older adults \| High \| In meta-analysis fixed effects model, the pooled effect for 10 μg/m3 growth in exposure to PM_10_ OR -0.09 95% CI -0.15- -0.03 \| \| **Meo (2024)**[28] \| PM_10_ \| Cognitive impairment \| Cohort/ Longitudinal \| 5 \| 3 (60.0) \| 471,301 \| Older adults \| Moderate \| In a random effect model, PM_10_ was associated with a non-significant increased risk of cognitive impairment (OR 1.37 95% CI 0.89–2.10) \| \|  \| PM_10_ \| Cognitive impairment \| Cross-sectional \| 4 \| 1 (25.0) \| 11,214 \| Older adults \| Moderate \| In a random effect model, PM_10_ was associated with a non-significant increased risk of cognitive impairment (OR 1.26 95% CI 0.68–2.33) \| \| **Mohammadzadeh (2024)**[29] \| PM_10_ \| Alzheimer’s dementia \| Mixed \| 8 \| NR \| 549,878 \| Older adults \| Moderate \| The majority of studies found PM_10_ exposure increased risk of Alzheimer’s dementia \| \|  \| PM_10_ \| Non-Alzheimer’s dementia \| Mixed \| 6 \| NR \| 558,629 \| Older adults \| Moderate \| PM_10_exposure may increase risk of non-Alzheimer’s dementia \| \| **Peters (2019)**[6] \| PM_10_ \| Memory \| Cohort/Longitudinal \| 1 \| 1 (100.0) \| 2,867 \| Older adults \| Moderate \| One study found an association between PM_10_ and memory decline \| \| **Power (2016)**[31] \| PM_10_ \| Cognitive level or decline \| Mixed \| 5 \| Mixed \| NR \| Adults or older adults \| Moderate \| The results were mixed with 2 studies finding no significant associations whilst other studies found an adverse effect of PM_10_ on at least one aspect of cognition \| \| **Tang (2022)**[20] \| PM_10_ T1 vs T2 \| Dementia \| Cohort/Longitudinal \| 2 \| 1 (50.0) \| 356,999 \| Adults \| Moderate \| No significant dose response relationship between PM_10_ and dementia in either analysis. \| \|  \| PM_10_ T1 vs T3 \| Dementia \| Cohort/Longitudinal \| 2 \| 2 (100.0) \| 356,999 \| Adults \| Moderate \| \|  \| PM_10_ \| Dementia \| Cohort/Longitudinal \| 2 \| 1 (50.0) \| 350,844 \| Adults \| Moderate \| No significant association between PM_10_ and dementia. \| \| **Xu (2024)**[34] \| PM_10_ \| Decreased cognitive function \| Cohort/Longitudinal \| 4 \| 2 (50.0) \| 30,604 \| NR \| Moderate \| Combined effect of RR 1.07 (95% CI 0.96-1.18) \| \|  \| PM_10_ \| Cognitive test scores \| Cohort/Longitudinal \| 6 \| 5 (83.3) \| 29,073 \| NR \| Moderate \| Combined effect of PM_10_ associated with a 0.15 point decrease in scores (95% CI -0.24 - -0.6) \| \| **Zhao (2021)**[8] \| PM_10_ \| Cognitive impairment and dementia \| Cohort/Longitudinal \| 8 \| NR \| 369,425 \| Adults and older adults \| Moderate \| No significant relationship was found between PM_10_ and any cognitive outcome \| \|  \| PM_10_ \| Cognitive impairment and dementia \| Mixed \| 5 \| NR \| 362,349 \| Adults and older adults \| Moderate \| \|  \| PM_10_ \| Vascular dementia \| Cohort/Longitudinal \| 4 \| NR \| 357,463 \| Adults and older adults \| Moderate \| \| **Air pollution – PMx/PM** \| \| \| \| \| \| \| \| \| \| \| **Chandra (2022)**[15] \| Prenatal exposure to PM \| Brain volume \| NR \| 1 \| 1 (100.0) \| NR \| Children \| High \| Prenatal PM exposure was found to have a deleterious impact on brain volumes, a dose-dependent thinning of cerebral cortex per 5μg/m3 increase in prenatal PM exposure in bilateral cerebral hemispheres was demonstrated in children \| \|  \| PM \| Cognitive impairment \| Mixed \| NR \| NR \| NR \| Adults and older adults \| High \| Particulate matter has been extensively investigated for a dose-response relationship with cognitive impairment in the elderly in several research projects in several high-income settings \| \| **Da (2025)**[4] \| PMx \| Dementia \| Mixed \| 14 \| 9 (64.3) \| NR \| Older adults \| High \| In meta-analysis random effects model RR 1.09 95% CI 1.06-1.12 \| \| **Killin (2016)**[38] \| PM \| Dementia \| Mixed \| 2 \| 1 (50.0) \| 96,534 \| Older adults \| Low \| This review found 'strong' evidence of positive association between particulate matter and dementia, but only across two studies \| \| **Air pollution - NO2 and NOx** \| \| \| \| \| \| \| \| \| \| \| **Abolhasani (2023)**[22] \| NO_x_ \| Dementia \| Mixed \| 4 \| 1 (25.0) \| 7,779 \| Older adults \| High \| Hazard Ratio of dementia per 10μg/m^3^ increase in NO_x_ was not significant HR 1.14 95% CI 0.99-1.31 \| \|  \| NO_2_ \| Dementia \| Mixed \| 5 \| 3 (60.0) \| 4,046,207 \| Older adults \| High \| Hazard Ratio of Dementia per 10μg/m^3^ increase in NO_2_ was significant HR 1.06 95% CI 1.01-1.11 \| \|  \| NO_2_ \| Non-Alzheimer’s disease dementia \| Mixed \| 1 \| 0 (0.0) \| 633,949 \| Older adults \| High \| Hazard Ratio of non-Alzheimer’s disease dementia per 10μg/m^3^ increase in NO_2_ was not significant HR 1.02 95% CI 0.99-1.06 \| \| **Clifford (2016)**[3] \| In utero NO_2_ \| Cognitive function \| Cohort/Longitudinal \| 3 \| 1 mixed \| 10,453 \| Children \| Moderate \| One of 3 studies found that in utero NO_2_ exposure was associated with reduced psychomotor speed \| \| **Da (2025)**[4] \| NOx \| Dementia \| NR \| 4 \| 3 (75.0) \| NR \| Older adults \| High \| In meta-analysis random effects model RR 1.1 95% CI 1.01-1.2 \| \| **Dhiman (2022)**[25] \| NO_2_ \| All-cause dementia \| Mixed \| 5 \| 1 (20.0) \| 2,588,101 \| Older adults \| Moderate \| The pooled hazard ratio for NO_2_ and all cause dementia is not significant HR 1.00 95% CI 1.00-1.01 \| \|  \| NO_x_ \| All-cause dementia \| Mixed \| 4 \| 0 (0.0) \| 357,059 \| Older adults \| Moderate \| The pooled hazard ratio for NO_x_ and all cause dementia is not significant HR 1.00 95% CI 1.00-1.01 \| \| **Fu (2020)**[16] \| NO_2_ \| Alzheimer’s disease \| Cohort/Longitudinal \|  \| 3 (75.0) \| 505,866 \| Older adults \| Moderate \| NO_2_ levels not associated with Alzheimer’s disease as they reported OR 1.00 95% 0.89-1.13 in random-effects meta-analysis \| \| **Killin (2016)**[38] \| NO_x_ \| Dementia \| Cohort/Longitudinal \| 2 \| 2 (100.0) \| 31,353 \| Older adults \| Low \| This review found 'strong' evidence of positive association between NO_x_ and dementia, but only across 2 studies \| \| **Mohammadzadeh (2024)**[29] \| NO_2_ and NO_x_ \| Alzheimer’s dementia \| Mixed \| 15 \| NR \| 34,084,659 \| Older adults \| Moderate \| The majority of studies found that NO_2_ and NO_x_ exposure increased risk of Alzheimer’s dementia \| \|  \| NO_2_ and NO_x_ \| Non-Alzheimer’s dementia \| Mixed \| 21 \| NR \| 13,635,952 \| Older adults \| Moderate \| The majority of studies found that NO_2_ and NO_x_ exposure increased risk of non-Alzheimer’s dementia \| \| **Oliveira (2024)**[18] \| NO_x_ \| Dementia (including Alzheimer’s disease) \| Mixed \| 12 \| 6 positive, 2 negative \| NR \| Adults \| Moderate \| Six studies found a significant association between NO_x_ and higher risk of dementia however, two studies found a significant association between NO_x_ and lower risk of dementia \| \| **Peters (2019)**[6] \| NOx or NO_2_ \| Incidence of dementia \| Cohort/Longitudinal \| 4 \| 3 + 1 mixed \| 2,228,633 \| Older adults \| Moderate \| The majority of studies found that there was an association between NO or NO_2_ and incidence of dementia \| \| **Tang (2022)**[20] \| NO_2_ T1 vs T2 \| Dementia \| Cohort/Longitudinal \| 6 \| 3 (50.0) \| 3,262,163 \| Adults \| Moderate \| Significant dose response relationship between NO_2_ and dementia in both T1 vs T2 and T1 vs T3 \| \|  \| NO_2_ T1 vs T3 \| Dementia \| Cohort/Longitudinal \| 6 \| 5 (83.0) \| 3,262,163 \| Adults \| Moderate \| \|  \| NO_x_ T1 vs T2 \| Dementia \| Cohort/Longitudinal \| 3 \| 3 (100.0) \| 355,577 \| Adults \| Moderate \| Significant dose response relationship between NO_x_ and dementia in both T1 vs T2 and T1 vs T3 \| \|  \| NO_x_ T1 vs T3 \| Dementia \| Cohort/Longitudinal \| 3 \| 3 (100.0) \| 355,577 \| Adults \| Moderate \| \| **Weuve (2021)**[33] \| NO_x_ \| Cognitive performance \| Mixed \| NR \| NR \| NR \| Adults and older adults \| Moderate \| Results on NO_2_ and NO_x_ were mixed. Some studies suggested adverse associations with cognitive levels, and others reported beneficial. \| \|  \| NO_x_ \| Incidence of cognitive impairment \| Mixed \| 3 \| NR \| NR \| Adults and older adults \| Moderate \| Associations with incidence of cognitive impairment (and NO_x_) were mixed. \| \| **Wilker (2023)**[39] \| NO_2_ \| Dementia \| Cohort/Longitudinal \| 9 \| 7 (77.8) \| 16,788,028 \| Older adults \| High \| Although there was a consistent association reported in each individual study, the pooled effect size did not reach significance. \| \|  \| NO_x_ \| Dementia \| Cohort/Longitudinal \| 5 \| 3 (60.0) \| 544,335 \| Older adults \| High \| \| **Xu (2024)**[34] \| NO_2_ \| Decreased cognitive function \| Cohort/Longitudinal \| 6 \| 0 (0.0) \| 61,420 \| NR \| Moderate \| Combined effect of RR 1.01 (95% CI 0.94-1.09) \| \|  \| NO_2_ \| Cognitive test scores \| Cohort/Longitudinal \| 6 \| 2 (33.3) \| 25,669 \| NR \| Moderate \| Combined effect was not significant (-0.01 95% CI -0.05-0.02) \| \| **Yu (2020)**[36] \| NO_2_/NO_x_ (per 5 ppb increment) \| Cognitive impairment \| Cohort/Longitudinal \| 7 \| 4 (57.1) \| 307,300 \| Older adults \| High \| No significant association was found in pooled analysis \| \| **Yuan (2024)**[37] \| NO_2_ \| Cognitive function \| Cohort/Longitudinal \| 1 \| 1 (100.0) \| 783 \| Children \| Moderate \| NO_2_ significantly associated with impaired inhibitory control \| \| **Zhao (2021)**[8] \| NO_2_ \| Dementia \| Mixed \| 8 \| NR \| 5,060,547 \| Adults and older adults \| Moderate \| A significant association was found between NO_2_ exposure and dementia. \| \|  \| NO_2_ \| Alzheimer’s disease \| Cohort/Longitudinal \| 6 \| NR \| 1,060,687 \| Adults and older adults \| Moderate \| No significant association found between NO_2_ exposure and Alzheimer’s disease. \| \|  \| NO_2_ \| Vascular dementia \| Mixed \| 5 \| NR \| 494,636 \| Adults and older adults \| Moderate \| A significant association was found between NO_2_ exposure and vascular dementia. \| \|  \| NO_x_ \| Dementia \| Mixed \| 4 \| NR \| 357,141 \| Adults and older adults \| Moderate \| No significant association was found between NO_x_ exposure and Alzheimer’s disease. \| \| **Air pollution - SO_2_** \| \| \| \| \| \| \| \| \| \| \| **Clifford (2016)**[3] \| In utero SO_2_ \| Cognitive function \| Cohort/Longitudinal \| 1 \| 1 (100.0) \| 533 \| Children \| Moderate \| In utero SO_2_ exposure was associated with reduced fine motor skills \| \| **Dhiman (2022)**[25] \| SO_2_ \| Vascular dementia \| Nested case-control \| 1 \| 0 (0.0) \| 4,155 \| Older adults \| Moderate \| The study did not find a significant association HR 0.95 95% CI 0.83-1.08 \| \| **Meo (2024)**[28] \| SO_2_ \| Cognitive impairment \| Cohort/Longitudinal \| 3 \| 2 (66.7) \| 36,223 \| Older adults \| Moderate \| In a fixed effect model, SO_2_ was associated with a significant increased risk of cognitive impairment (OR 1.39 95% CI 1.27-1.51) \| \| **Xu (2024)**[34] \| SO_2_ \| Cognitive test scores \| Cohort/Longitudinal \| 3 \| 2 (66.7) \| 20,172 \| NR \| Moderate \| Combined effect was not significant (-1.02 95% CI -2.79-0.76) \| \| **Zhao (2021)**[8] \| SO_2_ \| Cognitive impairment and dementia \| Mixed \| 3 \| NR \| 568,174 \| Adults and older adults \| Moderate \| Significant associations were found between SO_2_ exposure and cognitive impairment and dementia \| \| **Air pollution - Black carbon and elemental carbon** \| \| \| \| \| \| \| \| \| \| \| **Mohammadzadeh (2024)**[29] \| Black carbon \| Alzheimer’s dementia \| Mixed \| 2 \| NR \| 37,711,243 \| Older adults \| Moderate \| Insufficient information to comment \| \|  \| Black carbon \| Non-Alzheimer’s dementia \| Mixed \| 5 \| NR \| 4,668,029 \| Older adults \| Moderate \| Insufficient information to comment \| \| **Oliveria (2024)**[18] \| Black carbon \| Dementia (including Alzheimer’s disease) \| Mixed \| 3 \| 0 (0.0) \| NR \| Adults \| Moderate \| No association between black carbon and dementia \| \| **Yuan (2024)**[37] \| Elemental carbon \| Cognitive function \| Case-control \| 1 \| 1 (100.0) \| 263 \| Children \| Moderate \| Elemental carbon significantly associated with lower motor response speed \| \| **Air pollution - CO** \| \| \| \| \| \| \| \| \| \| \| **Clifford (2016)**[3] \| In utero CO \| Cognitive function \| Cohort/Longitudinal \| 1 \| 0 (0.0) \| 533 \| Children \| Moderate \| No significant findings \| \| **Dhiman (2022)**[25] \| CO \| All-cause dementia \| Cohort/Longitudinal \| 1 \| 0 (0.0) \| 547 \| Older adults \| Moderate \| The study did not find a significant association HR 1.07 95% CI 0.92-1.25 \| \|  \| CO \| Vascular dementia \| Nested case-control \| 1 \| 0 (0.0) \| 4,155 \| Older adults \| Moderate \| The study did not find a significant association OR 1.78 95% CI 0.56-5.61 \| \| **Killin (2016)**[38] \| CO \| Dementia \| Cohort/Longitudinal \| 1 \| 1 (100.0) \| 29,537 \| Older adults \| Low \| This review found 'moderate' evidence of positive association between CO and dementia, but only across 1 study. \| \| **Mohammadzadeh (2024)**[29] \| CO \| Alzheimer’s dementia \| Mixed \| 3 \| NR \| 18,877 \| Older adults \| Moderate \| CO exposure may be associated with risk of Alzheimer’s dementia \| \|  \| CO \| Non-Alzheimer’s dementia \| Mixed \| 2 \| NR \| 33,702 \| Older adults \| Moderate \| CO exposure may be associated with non-Alzheimer’s dementia \| \| **Tang (2022)**[20] \| CO T1 vs T2 \| Dementia \| Cohort/Longitudinal \| 2 \| 2 (100.0) \| 35,702 \| Adults \| Moderate \| Significant dose response relationship between CO and dementia in T1 vs T3 but not T1 vs T3 \| \|  \| CO T1 vs T3 \| Dementia \| Cohort/Longitudinal \| 2 \| 1 (50.0) \| 35,702 \| Adults \| Moderate \| \| **Zhao (2021)**[8] \| CO \| Cognitive impairment and dementia \| Cohort/Longitudinal \| 4 \| NR \| 41,167 \| Adults and older adults \| Moderate \| Significant associations were found between CO exposure and cognitive impairment and dementia \| \| **Air pollution - O_3_** \| \| \| \| \| \| \| \| \| \| \| **Abolhasani (2023)**[22] \| O_3_ \| Dementia \| Mixed \| 2 \| 0 (0.0) \| 2,197,617 \| Older adults \| High \| Hazard Ratio of dementia per 10μg/m^3^ increase in O_3_ was not significant HR 0.87 95% CI 0.7-1.09 \| \|  \| O_3_ \| Alzheimer’s disease \| NR \| 1 \| 1 (100.0) \| 95,690 \| Older adults \| High \| Hazard Ratio of Alzheimer’s disease per 10μg/m^3^increase in O_3_ was significant HR 1.13 95% CI 1.01-1.26 although this was only one study. \| \| **Clifford (2016)**[3] \| In utero O_3_ \| Cognitive function \| Cohort/Longitudinal \| 1 \| 0 (0.0) \| 533 \| Children \| Moderate \| No significant findings \| \| **Dhiman (2022)**[25] \| O_3_ \| Alzheimer’s disease \| Mixed \| 4 \| 2 (50.0) \| 578,383 \| Older adults \| Moderate \| The pooled hazard ratio for O_3_ and Alzheimer’s disease is not significant HR 1.02 95% CI 0.96-1.08 \| \| **Killin (2016)**[38] \| O_3_ \| Dementia \| Mixed \| 2 \| 1 (50.0) \| 96,561 \| Older adults \| Low \| This review found 'strong' evidence of positive association between O_3_ and dementia, but only across 2 studies \| \| **Meo (2024)**[28] \| O_3_ \| Cognitive impairment \| Cohort/Longitudinal \| 4 \| 2(50.0) \| 19,613 \| Older adults \| Moderate \| In a random effect model, O_3_ was associated with a non-significant increased risk of cognitive impairment (OR 1.00; 95% CI 0.54-1.87) \| \| **Mohammadzadeh (2024)**[29] \| O_3_ \| Alzheimer’s dementia \| Mixed \| 10 \| NR \| 33,963,332 \| Older adults \| Moderate \| O_3_ exposure may increase risk of Alzheimer’s dementia \| \|  \| O_3_ \| Non-Alzheimer’s dementia \| Mixed \| 9 \| NR \| 3,221,716 \| Older adults \| Moderate \| O_3_ exposure may be associated with non-Alzheimer’s dementia \| \| **Peters (2019)**[6] \| O_3_ \| Cognitive decline, dementia, or Alzheimer’s disease \| Cohort/Longitudinal \| 4 \| 2 + 1 mixed \| 2,298,423 \| Older adults \| Moderate \| Some studies found significant associations between O_3_   and cognition but the directionality was not consistent \| \| **Power (2016)**[31] \| O_3_ \| Dementia-related cognitive outcomes \| Mixed \| 3 \| 2 (66.7) \| NR \| Adults or older adults \| Moderate \| One study found an adverse association between O_3_   exposure and cognitive level and one study found higher O_3_   exposure associated with greater risk of dementia diagnosis whilst the final study found no association between ozone and cognitive level \| \| **Tang (2022)**[20] \| O_3_ T1 vs T2 \| Dementia \| Cohort/Longitudinal \| 5 \| 3 (60.0) \| 2,650,306 \| Adults \| Moderate \| No significant dose response relationship between O_3_ and dementia in any analysis \| \|  \| O_3_ T1 vs T3 \| Dementia \| Cohort/Longitudinal \| 5 \| 2 (40.0) \| 2,650,306 \| Adults \| Moderate \| \| **Weuve (2021)**[33] \| O_3_ \| Cognitive decline \| Mixed \| 5 \| NR \| NR \| Adults and older adults \| Moderate \| This review reported that none of the findings were consistent or pertained to cognitive decline \| \| **Xu (2024)**[34] \| O_3_ \| Decreased cognitive function \| Cohort/Longitudinal \| 5 \| 3 (60.0) \| 67,674 \| NR \| Moderate \| Combined effect of RR 1.19 (95% CI 1.04-1.37) \| \|  \| O_3_ \| Cognitive test scores \| Cohort/Longitudinal \| 3 \| 3 (different directions) \| 20,172 \| NR \| Moderate \| Combined effect was not significant (0.06 95%CI -0.55-0.66). \| \| **Yu (2020)**[36] \| O_3_ (pre 5 ppb increment) \| Cognitive impairment \| Cohort/Longitudinal \| 4 \| 3 (75.0) \| 2,644,151 \| Older adults \| High \| No association was found in pooled analysis \| \| **Zhao (2021)**[8] \| O_3_ \| Cognitive impairment and dementia \| Cohort/Longitudinal \| 8 \| NR \| 2,653,070 \| Adults and older adults \| Moderate \| All analyses between O_3_ exposure and cognitive impairment and dementia were significant \| \|  \| O_3_ \| Cognitive impairment and dementia \| Mixed \| 5 \| NR \| 2,648,044 \| Adults and older adults \| Moderate \| \| **Zhao (2018)**[40] \| O_3_ \| Cognitive impairment \| Cross-sectional \| 2 \| 2 (100.0) \| 3,260 \| Older adults \| Moderate \| Two cross-sectional studies found a correlation between O_3_ and impairment of cognitive functions. \| \|  \| O_3_ \| Dementia \| Mixed \| 5 \| 4 (80.0) \| 2,074,104 \| Older adults \| Moderate \| Four studies found correlations between O_3_ and dementia. \| \| **Air pollution – Traffic-related** \| \| \| \| \| \| \| \| \| \| \| **Chandra (2022)**[15] \| Prenatal exposure to traffic-related air pollution \| Intelligence quotient \| Mixed \| 2 \| 2 (100.0) \| NR \| Children \| High \| Prenatal exposure to traffic-related air pollution was found to have an adverse impact on intelligence quotient in 2 separate birth cohort studies in the USA and Italy \| \|  \| Postnatal exposure to traffic-related air pollution \| Cognitive and developmental indicators \| Mixed \| 4 \| 3 (75.0) \| NR \| Children \| High \| Postnatal traffic-related air pollution was found to be associated with impaired cognitive performance in 4 studies, though there was one contradictory study \| \| **Debelu (2024)**[41] \| Traffic-related PM_2.5_ and PM_10_ \| Cognitive function \| NR \| 1 \| 1 (100.0) \| 2,867 \| Adults \| Moderate \| Increased PM_2.5_ (1.1 μg/m^3^ (lag4)) associated with decline in standardized memory score (5-year decline) (OR = 0.03 95% CI 0.06-0.002) \| \|  \| Traffic-related NOx \| Dementia (Alzheimer’s disease or vascular dementia) \| NR \| 1 \| 0 (0.0) \| 1,806 \| All ages \| Moderate \| Participants in the group with the highest exposure were more likely to be diagnosed with dementia than those in the group with the lowest exposure but this was non-significant HR 1.43 95% CI 0.998-2.05 for the highest vs the lowest quartile). \| \|  \| Traffic-related elemental carbon and NO_2_ \| Cognitive development (working memory) \| NR \| 1 \| NR \| 2,715 \| Children \| Moderate \| Children attending schools with higher levels of elemental carbon and NO_2_, outdoors experienced substantially smaller growth in all the cognitive measurements, statistics NR \| \| **Dimakakou (2018)**[5] \| Traffic-related air pollution \| Dementia \| Mixed \| 9 \| NR \| NR \| Adults and older adults \| Moderate \| The findings supported an association between air pollution and dementia related outcomes traffic related air pollution \| \| **Killin (2016)**[38] \| Diesel motor exhaust \| Non-vascular dementia \| Cohort/Longitudinal \| 1 \| NR \| 1,552 \| Older adults \| Low \| This review found 'moderate' evidence of no association between diesel motor exhaust and non-vascular dementia, but only across one study \| \| **Power (2016)**[31] \| Traffic-related pollution \| Dementia-related cognitive outcomes \| Mixed \| 6 \| Mixed \| NR \| Adults or older adults \| Moderate \| Five of the six studies found an adverse effect of traffic-related pollution and at least one aspect of cognition whilst the final study found that traffic-related particulate matter was associated with lower cognitive level but not cognitive decline \| \|  \| Traffic-related NO_2_ or NO_x_ \| Cognitive impairment \| Mixed \| 2 \| 2 (100.0) \| NR \| Adults or older adults \| Moderate \| Both studies found an adverse association between traffic-related NO_x_ or NO_2_ and cognitive impairment \| \| **Yuan (2024)**[37] \| Traffic-related PM_10_and NO_2_ \| Vascular dementia \| Cohort/Longitudinal \| 1 \| 1 (100.0) \| 5,888 \| Adults and older adults \| Moderate \| Long-term exposure to PM_10_ and NO_2_ was significantly associated with increased risk of vascular dementia \| \| **Noise – Environmental** \| \| \| \| \| \| \| \| \| \| \| **Clark (2020)**[42] \| Environmental noise \| Vascular dementia \| Mixed \| 2 \| NR \| NR \| Adults and older adults \| Moderate \| Low quality evidence of no effect between noise exposure and vascular dementia incidence \| \|  \| Environmental noise \| Cognitive assessment of dementia symptoms \| NR \| 1 \| NR \| NR \| Adults and older adults \| Moderate \| Very low-quality evidence of a harmful effect of environmental noise exposure and cognitive assessment of dementia symptoms \| \| **Da (2024)**[4] \| Noise \| Dementia \| Cohort/Longitudinal \| 2 \| 1 (50.0) \| 2,616,994 \| Older adults \| High \| In meta-analysis random effect model RR 1.09 95%CI 0.94-1.27 \| \| **Killin (2016)**[38] \| Excessive noise \| Alzheimer’s disease \| Cohort/Longitudinal \| 1 \| 1 (100.0) \| 694 \| Older adults \| Low \| This review found 'moderate' evidence of negative association between excessive noise and Alzheimer’s disease, but only across one study \| \| **Thompson (2022)**[43] \| Environmental noise \| Cognitive impairment \| Cross-sectional \| 3 \| 1 (33.3) \| 9,565 \| Adults and older adults \| Moderate \| Odds of cognitive impairment were significantly higher for higher compared to lower noise exposure in one study \| \|  \| Environmental noise \| Reading and language abilities \| Cross-sectional \| 4 \| 0 (0.0) \| 13,802 \| Children \| Moderate \| No significant associations were found between environmental noise (impact if 1db increase from mixed transport sources) and reading and language abilities \| \| **Zhao (2021)**[8] \| Environmental noise \| Cognitive impairment and dementia \| Cohort/Longitudinal \| 6 \| NR \| 772,857 \| Adults and older adults \| Moderate \| Significant associations were found between noise exposure and cognitive impairment and dementia, but only in cohort studies \| \|  \| Environmental noise \| Dementia \| Mixed \| 4 \| NR \| 767,342 \| Adults and older adults \| Moderate \| \| **Noise – Traffic-related** \| \| \| \| \| \| \| \| \| \| \| **Dimakakou (2018)**[5] \| Road traffic noise \| Mild cognitive impairment \| NR \| 1 \| NR \| NR \| Adults and older adults \| Moderate \| One study supported a positive association of mild cognitive impairment and road traffic noise \| \| **Hegewald (2020)**[44] \| Road traffic noise \| Dementia \| Mixed \| 2 \| NR \| 136,299 \| Older adults \| Moderate \| Studies on dementia found no evidence for a road-traffic related dementia risk. \| \|  \| Road traffic noise \| Mild cognitive impairment \| Cross-sectional \| 1 \| 1 (100.0) \| 2,050 \| Older adults \| Moderate \| The study found for road traffic noise levels of 60 dB L_DEN_ or more (OR = 1.40; 95% CI 1.03–1.91) and L_NIGHT_ levels of 55 dB or more (OR = 1.80; 95% CI 1.07–3.04) \| \|  \| Road traffic noise \| Cognitive function \| Cohort/Longitudinal \| 1 \| NR \| 288 \| Older adults \| Moderate \| The study found road traffic noise had an influence on cognitive function L_DEN_, but not for L_NIGHT._No statistics reported. \| \| **Tzivian (2015)**[21] \| Road traffic noise \| Cognitive performance \| NR \| 1 \| 1 (100.0) \| 81 \| Adults \| Moderate \| One study in this review looked at noise and cognitive performance, which found that police officers from high traffic areas versus office workers had worse cognitive performance outcomes \| \| **Temperature** \| \| \| \| \| \| \| \| \| \| \| **Byun (2024)**[45] \| 1 degree Celsius higher ambient temperature \| Dementia \| Mixed \| 4 \| 4 (100.0) \| NR \| Older adults \| High \| Higher temperatures were significantly associated with hospital admissions or mortality due to dementia \| \|  \| Higher ambient temperature \| Dementia \| Mixed \| 2 \| 1 (50.0) \| 3,442,281 \| Older adults \| High \| One of two studies found that higher compared to lower temperature exposure was associated with hospital admissions or mortality due to dementia \| \|  \| Heat wave \| Dementia \| Mixed \| 4 \| 3 (75.0) \| NR \| Older adults \| High \| The majority of studies found that heat waves compared to no heat waves were associated with hospital admissions or mortality due to dementia \| \| **Oliveira (2024)**[18] \| Temperature \| Dementia (including Alzheimer’s disease) \| Mixed \| 2 \| 0 (0.0) \| NR \| Adults \| Moderate \| No associations between temperature and dementia \| \| **Zhao (2021)**[8] \| Higher temperature \| Cognitive impairment and dementia \| Cohort/Longitudinal \| 3 \| NR \| 3,089,076 \| Adults and older adults \| Moderate \| No significant association between higher temperature and cognitive impairment and dementia. \| \| **Chemicals and solvents** \| \| \| \| \| \| \| \| \| \| \| **Chandra (2022)**[15] \| Prenatal exposure to isophorone \| Mathematics performance \| NR \| 1 \| 1 (100.0) \| NR \| Children \| High \| High isophorone level (>0.49 ng/m3) was associated with low performance on mathematics tasks among children from urban and highly populated urban areas, with their scores being reduced by 1.19 points (95% CI:−1.94,−0.44). \| \|  \| Prenatal exposure to persistent organic pollutants \| Cognitive and developmental indicators \| NR \| 1 \| 1 (100.0) \| NR \| Children \| High \| There was a significant association between the compound polychlorinated biphenyls -183 and lower total intelligence, hexabromocyclododecane and lower performance intelligence, and polybrominated diphenyl ethers with lower verbal memory. Several hydroxy polychlorinated biphenyls were associated with more optimal sustained attention and balance. \| \|  \| Prenatal exposure to polycyclic aromatic hydrocarbons \| Cognitive and developmental indicators \| Mixed \| 3 \| 3 (100.0) \| NR \| Children \| High \| Prenatal exposure to polycyclic aromatic hydrocarbons is another set of pollutants implicated in lower intelligence quotient, neuroimaging markers, impaired cognition in children and behavioural conditions such as attention deficit hyperactivity disorder and Conduct Disorder in separate birth cohort studies and different ethnic populations. \| \|  \| Postnatal exposure to persistent organic pollutants \| Cognitive and developmental indicators \| NR \| 1 \| 1 (100.0) \| NR \| Children \| High \| Like prenatal exposure, postnatal exposure to different persistent organic pollutants has been found to have an adverse cognitive impact \| \|  \| Postnatal exposure to isophorene \| Mathematics performance \| NR \| 1 \| 1 (100.0) \| NR \| Children \| High \| Like prenatal exposure, postnatal isophorene has also been found to be associated with lower performance on mathematical tasks in children \| \|  \| Polyaromatic Hydrocarbons \| Cognitive performance \| NR \| 1 \| 1 (100.0) \| NR \| Older adults \| High \| A dose-dependent association between polyaromatic hydrocarbons exposure per 1% increase (assessed by urinary 1-hydroxypyrene) and a decline in performance \| \| **Clifford (2016)**[3] \| In utero polycyclic aromatic hydrocarbons \| Cognitive function \| Cohort/Longitudinal \| 2 \| 1+1 mixed \| 397 \| Children \| Moderate \| In utero exposure to polycyclic aromatic hydrocarbons was associated with reduced intelligence scores in children at 3 and 5 years of age but not 1 and 2 years of age \| \|  \| In utero Benzene \| Cognitive function \| Cohort/Longitudinal \| 1 \| 0 (0.0) \| 438 \| Children \| Moderate \| No significant findings \| \| **Mohammadzadeh (2024)**[29] \| Solvents (Benzene, Toluene and Phenols) \| Alzheimer’s dementia \| Case-control \| 1 \| 1 (100.0) \| 436 \| Older adults \| Moderate \| Prior exposure to solvents was associated with increased risk of Alzheimer’s dementia \| \| **Pesticides and heavy metals** \| \| \| \| \| \| \| \| \| \| \| **Olayinka (2019)**[46] \| Pesticide \| Alzheimer’s disease \| Mixed \| 6 \| 3 (50.0) \| 51,359 \| Older adults \| Moderate \| Three studies found statistically significant associations between pesticide exposure and Alzheimer’s disease \| \|  \| Lead and mercury \| Alzheimer’s disease \| Mixed \| 2 \| 0 (0.0) \| 600 \| Older adults \| Moderate \| No significant associations were found for occupational exposures to lead and mercury \| \| CI=Confidence Interval; CO=Carbon Monoxide; db=decibel; HR=Hazard Ratio; JBI= Joanna Briggs Institute; L_DEN_=Noise level over the day; L_NIGHT_=Noise level over the night ;m^3^=meters cubed; MCI=Mild Cognitive Impairment; NDVI= Normalised Difference Vegetation Index;NO_2_=Nitrogen Dioxide; NO_x_=Nitrogen Oxides; NR=Not Reported due to insufficient data for curation or missing data;O_3_=Ozone; OR= Odds Ratio; PM/PM_x_=Particulate Matter of non-descript size;PM_10_=Particulate Matter less than 10 micrometers in diameter;PM_2.5_= Particulate Matter less than 2.5 micrometers in diameter; ppb=parts per billion; RR=Risk Ratio;SO_2_=Sulfur Dioxide; Tn (e.g., T1)=Tertile 1;ug=microgram; UK= United Kingdom; USA= Unites States of America; vs=versus \| \| \| \| \| \| \| \| \| \| \| *percentage not reported for reviews which had any mixed results \| \| \| \| \| \| \| \| \| \|        \| **Appendix 4 Table 3. Data extraction - Social environment outcomes** \| \| \| \| \| \| \| \| \| \| \| --- \| --- \| --- \| --- \| --- \| --- \| --- \| --- \| --- \| --- \| \| **First author (year of publication)** \| **Environmental exposure** \| **Outcome(s)** \| **Study design(s)** \| **Total included studies** \| ***Studies reporting statistically significant results n (%)** \| **Total participants** \| **Age category** \| **JBI critical appraisal quality score** \| **Summary of results** \| \| **Socio-economic status and deprivation** \| \| \| \| \| \| \| \| \| \| \| **McGrattan (2021)**[47] \| Socio-economic status \| Mild cognitive impairment \| Mixed \| NR \| 4 \| NR \| Older adults \| Moderate \| Low socio-economic status was a significant risk factor for mild cognitive impairment in low-middle income countries. \| \| **Michael (2024)**[10] \| Neighbourhood socio-economic status \| Cognitive function, cognitive decline, or dementia \| Cohort/Longitudinal \| 9 \| 3 + 3 mixed \| NR \| Older adults \| Moderate \| Some studies found that neighbourhood socio-economic status was associated with cognition, other studies had conflicting findings \| \| **Rodrigues (2025)**[48] \| Social exclusion in economic domain (poverty, socio-economic status) \| Cognitive decline, cognitive impairment \| Cohort/Longitudinal \| 12 \| 10 (83.3) \| 59,588 \| Older adults \| Moderate \| Higher income status was associated with a lower risk of cognitive decline \| \| **Wang (2023)**[49] \| Socio-economic status \| Cognitive impairment \| Cohort/Longitudinal \| 14 \| NR \| 1,391,338 \| Older adults \| Moderate \| A significant relationship was found between socio-economic status and cognitive impairment. \| \|  \| Socio-economic status \| All cause dementia \| Cohort/Longitudinal \| 16 \| NR \| 89,808 \| Older adults \| Moderate \| A significant relationship was found between socio-economic status and all cause dementia. \| \|  \| Socio-economic status \| Alzheimer’s disease \| Cohort/Longitudinal \| 5 \| NR \| 7,276 \| Older adults \| Moderate \| A significant relationship was found between socio-economic status and Alzheimer’s disease. \| \| **Wu (2015)**[50] \| Area deprivation \| Cognitive functioning \| Mixed \| 11 \| 8 (72.8) \| NR \| Older adults \| Low \| Significant associations were found in eight studies with regards to local deprivation and poor cognitive function, decline or higher risk of cognitive impairment \| \| **Zhao (2021)**[8] \| Socio-economic status \| Alzheimer’s disease \| Cohort/Longitudinal \| 17 \| NR \| 911,010 \| Adults and older adults \| Moderate \| A significant association was found between socio-economic status and Alzheimer’s disease \| \|  \| Socio-economic status \| Cognitive impairment and dementia \| Mixed \| 8 \| NR \| 279,610 \| Adults and older adults \| Moderate \| A significant association was found between socio-economic status and cognitive impairment and dementia \| \| **Social contact, social relationships, and social support** \| \| \| \| \| \| \| \| \| \| \| **Kuiper (2016)**[51] \| Combination of structural and functional aspects of social relationships \| Cognitive decline \| Cohort/Longitudinal \| 7 \| 5 (71.4) \| 14,027 \| Older adults \| Moderate \| There was a significant relationship between combined structural and functional aspects of social relationships \| \|  \| Functional aspects of social relationships \| Cognitive decline \| Cohort/Longitudinal \| 8 \| 2 (25.0) \| 5,367 \| Older adults \| Moderate \| There was a significant relationship between functional aspects of social relationships and cognitive decline \| \|  \| Structural aspects of social relationships \| Cognitive decline \| Cohort/Longitudinal \| 21 \| 9 (42.9) \| 26,430 \| Older adults \| Moderate \| Poor structural social relationships were significantly associated with cognitive decline \| \| **Kuiper (2015)**[52] \| Satisfaction with social network \| Incidence of dementia \| Cohort/Longitudinal \| 4 \| 1 (25.0) \| 6,207 \| Older adults \| Moderate \| No significant relationship was found between satisfaction with social network and incidence of dementia \| \|  \| Social contact \| Incidence of dementia \| Cohort/Longitudinal \| 8 \| 4 (50.0) \| 15,762 \| Older adults \| Moderate \| A significant relationship was found between frequency of social contact and risk of dementia \| \|  \| Social network size \| Incidence of dementia \| Cohort/Longitudinal \| 5 \| 1 (20.0) \| 7,750 \| Older adults \| Moderate \| No significant relationship was found between social network size and dementia \| \|  \| Social participation \| Incidence of dementia \| Cohort/Longitudinal \| 6 \| 3 (60.0) \| 7,714 \| Older adults \| Moderate \| A significant relationship was found between social participation and risk of dementia \| \| **McGrattan (2021)**[47] \| Social contact \| Mild cognitive impairment \| Mixed \| NR \| 2 \| NR \| Older adults \| Moderate \| Maintaining social contact with others was stated as a protective risk factor \| \| **Plassman (2010)**[53] \| Social engagement \| Cognitive decline \| Cohort/Longitudinal \| 15 \| NR \| 42,950 \| Adults and older adults \| Moderate \| No consistent association for marital status, social network, or social support was found \| \| **Rodrigues (2025)**[48] \| Civic engagement \| Cognitive decline, cognitive impairment \| Cohort/Longitudinal \| 17 \| 9 (52.9) \| 176,969 \| Older adults \| Moderate \| All studies with >11 years follow up found that volunteering for example was associated with reduced cognitive decline \| \|  \| Social relations \| Cognitive decline, cognitive impairment \| Cohort/Longitudinal \| 34 \| NR \| 126,816 \| Older adults \| Moderate \| There were mixed findings in associations between social exclusion and cognition, studies with follow up >11 years consistently reported significant associations \| \|  \| Multidimensional social exclusion \| Cognitive impairment \| Cohort/Longitudinal \| 1 \| 1 (100.0) \| 10,932 \| Older adults \| Moderate \| Social exclusion increased the risk of cognitive impairment by 80% (OR 1.8 p<0.001) \| \| **Zhao (2021)**[8] \| Community engagement \| Cognitive impairment and dementia \| Cohort/Longitudinal \| 2 \| NR \| 10,137 \| Adults and older adults \| Moderate \| Both community engagement and community groups were protective factors for all analyses \| \|  \| Community group \| Dementia \| Cohort/Longitudinal \| 2 \| NR \| 23,534 \| Adults and older adults \| Moderate \| \|  \| Social contact \| Dementia \| Cohort/Longitudinal \| 10 \| NR \| 33,063 \| Adults and older adults \| Moderate \| All types of social contact variables were found to be protective factors for dementia, and Alzheimer’s disease. \| \|  \| Social contact \| Alzheimer’s disease \| Cohort/Longitudinal \| 2 \| NR \| 2,875 \| Adults and older adults \| Moderate \| \|  \| Social contact with friends \| Dementia \| Cohort/Longitudinal \| 3 \| NR \| 22,583 \| Adults and older adults \| Moderate \| \|  \| Social contact with relatives \| Dementia \| Cohort/Longitudinal \| 3 \| NR \| 22,730 \| Adults and older adults \| Moderate \| \| **Social isolation and loneliness** \| \| \| \| \| \| \| \| \| \| \| **Hamrah (2023)**[54] \| Social isolation \| Dementia \| Mixed \| 6 \| NR \| NR \| Adults and older adults \| Moderate \| Mixed results were reported for associations between social isolation and dementia in refugees, migrants, and asylum seekers in Australia \| \| **Kuiper (2015)**[52] \| Loneliness \| Incidence of dementia \| Cohort/Longitudinal \| 3 \| 1 (33.3) \| 3,252 \| Older adults \| Moderate \| Significant relationship between loneliness and risk of dementia \| \| **Traffic safety** \| \| \| \| \| \| \| \| \| \| \| **Chen (2022)**[2] \| Traffic safety \| Cognitive performance \| NR \| 1 \| 0 (0.0) \| NR \| Older adults \| Moderate \| Traffic safety was not found to be associated with cognition among older adults. \| \| CI=Confidence Interval; CO=Carbon Monoxide; db=decibel; HR=Hazard Ratio; JBI=Joanna Briggs Institute; L_DEN_=Noise level over the day; L_NIGHT_=Noise level over the night ;m^3^=meters cubed; MCI=Mild Cognitive Impairment; NDVI= Normalised Difference Vegetation Index;NO_2_=Nitrogen Dioxide; NO_x_=Nitrogen Oxides; NR=Not Reported due to insufficient data for curation or missing data;O_3_=Ozone; OR= Odds Ratio; PM/PM_x_=Particulate Matter of non-descript size; PM_10_=Particulate Matter less than 10 micrometers in diameter; PM_2.5_= Particulate Matter less than 2.5 micrometers in diameter; ppb=parts per billion; RR=Risk Ratio; SO_2_=Sulfur Dioxide; Tn (e.g., T1)=Tertile 1; ug=microgram; UK= United Kingdom; USA= Unites States of America; vs=versus \| \| \| \| \| \| \| \| \| \| \| *percentage not reported for reviews which had any mixed results \| \| \| \| \| \| \| \| \| \| |
| --- | --- | --- | --- | --- | --- | --- | --- | --- | --- | --- | --- | --- | --- | --- | --- | --- | --- | --- | --- | --- | --- | --- | --- | --- | --- | --- | --- | --- | --- | --- | --- | --- | --- | --- | --- | --- | --- | --- | --- | --- | --- | --- | --- | --- | --- | --- | --- | --- | --- | --- | --- | --- | --- | --- | --- | --- | --- | --- | --- | --- | --- | --- | --- | --- | --- | --- | --- | --- | --- | --- | --- | --- | --- | --- | --- | --- | --- | --- | --- | --- | --- | --- | --- | --- | --- | --- | --- | --- | --- | --- | --- | --- | --- | --- | --- | --- | --- | --- | --- | --- | --- | --- | --- | --- | --- | --- | --- | --- | --- | --- | --- | --- | --- | --- | --- | --- | --- | --- | --- | --- | --- | --- | --- | --- | --- | --- | --- | --- | --- | --- | --- | --- | --- | --- | --- | --- | --- | --- | --- | --- | --- | --- | --- | --- | --- | --- | --- | --- | --- | --- | --- | --- | --- | --- | --- | --- | --- | --- | --- | --- | --- | --- | --- | --- | --- | --- | --- | --- | --- | --- | --- | --- | --- | --- | --- | --- | --- | --- | --- | --- | --- | --- | --- | --- | --- | --- | --- | --- | --- | --- | --- | --- | --- | --- | --- | --- | --- | --- | --- | --- | --- | --- | --- | --- | --- | --- | --- | --- | --- | --- | --- | --- | --- | --- | --- | --- | --- | --- | --- | --- | --- | --- | --- | --- | --- | --- | --- | --- | --- | --- | --- | --- | --- | --- | --- | --- | --- | --- | --- | --- | --- | --- | --- | --- | --- | --- | --- | --- | --- | --- | --- | --- | --- | --- | --- | --- | --- | --- | --- | --- | --- | --- | --- | --- | --- | --- | --- | --- | --- | --- | --- | --- | --- | --- | --- | --- | --- | --- | --- | --- | --- | --- | --- | --- | --- | --- | --- | --- | --- | --- | --- | --- | --- | --- | --- | --- | --- | --- | --- | --- | --- | --- | --- | --- | --- | --- | --- | --- | --- | --- | --- | --- | --- | --- | --- | --- | --- | --- | --- | --- | --- | --- | --- | --- | --- | --- | --- | --- | --- | --- | --- | --- | --- | --- | --- | --- | --- | --- | --- | --- | --- | --- | --- | --- | --- | --- | --- | --- | --- | --- | --- | --- | --- | --- | --- | --- | --- | --- | --- | --- | --- | --- | --- | --- | --- | --- | --- | --- | --- | --- | --- | --- | --- | --- | --- | --- | --- | --- | --- | --- | --- | --- | --- | --- | --- | --- | --- | --- | --- | --- | --- | --- | --- | --- | --- | --- | --- | --- | --- | --- | --- | --- | --- | --- | --- | --- | --- | --- | --- | --- | --- | --- | --- | --- | --- | --- | --- | --- | --- | --- | --- | --- | --- | --- | --- | --- | --- | --- | --- | --- | --- | --- | --- | --- | --- | --- | --- | --- | --- | --- | --- | --- | --- | --- | --- | --- | --- | --- | --- | --- | --- | --- | --- | --- | --- | --- | --- | --- | --- | --- | --- | --- | --- | --- | --- | --- | --- | --- | --- | --- | --- | --- | --- | --- | --- | --- | --- | --- | --- | --- | --- | --- | --- | --- | --- | --- | --- | --- | --- | --- | --- | --- | --- | --- | --- | --- | --- | --- | --- | --- | --- | --- | --- | --- | --- | --- | --- | --- | --- | --- | --- | --- | --- | --- | --- | --- | --- | --- | --- | --- | --- | --- | --- | --- | --- | --- | --- | --- | --- | --- | --- | --- | --- | --- | --- | --- | --- | --- | --- | --- | --- | --- | --- | --- | --- | --- | --- | --- | --- | --- | --- | --- | --- | --- | --- | --- | --- | --- | --- | --- | --- | --- | --- | --- | --- | --- | --- | --- | --- | --- | --- | --- | --- | --- | --- | --- | --- | --- | --- | --- | --- | --- | --- | --- | --- | --- | --- | --- | --- | --- | --- | --- | --- | --- | --- | --- | --- | --- | --- | --- | --- | --- | --- | --- | --- | --- | --- | --- | --- | --- | --- | --- | --- | --- | --- | --- | --- | --- | --- | --- | --- | --- | --- | --- | --- | --- | --- | --- | --- | --- | --- | --- | --- | --- | --- | --- | --- | --- | --- | --- | --- | --- | --- | --- | --- | --- | --- | --- | --- | --- | --- | --- | --- | --- | --- | --- | --- | --- | --- | --- | --- | --- | --- | --- | --- | --- | --- | --- | --- | --- | --- | --- | --- | --- | --- | --- | --- | --- | --- | --- | --- | --- | --- | --- | --- | --- | --- | --- | --- | --- | --- | --- | --- | --- | --- | --- | --- | --- | --- | --- | --- | --- | --- | --- | --- | --- | --- | --- | --- | --- | --- | --- | --- | --- | --- | --- | --- | --- | --- | --- | --- | --- | --- | --- | --- | --- | --- | --- | --- | --- | --- | --- | --- | --- | --- | --- | --- | --- | --- | --- | --- | --- | --- | --- | --- | --- | --- | --- | --- | --- | --- | --- | --- | --- | --- | --- | --- | --- | --- | --- | --- | --- | --- | --- | --- | --- | --- | --- | --- | --- | --- | --- | --- | --- | --- | --- | --- | --- | --- | --- | --- | --- | --- | --- | --- | --- | --- | --- | --- | --- | --- | --- | --- | --- | --- | --- | --- | --- | --- | --- | --- | --- | --- | --- | --- | --- | --- | --- | --- | --- | --- | --- | --- | --- | --- | --- | --- | --- | --- | --- | --- | --- | --- | --- | --- | --- | --- | --- | --- | --- | --- | --- | --- | --- | --- | --- | --- | --- | --- | --- | --- | --- | --- | --- | --- | --- | --- | --- | --- | --- | --- | --- | --- | --- | --- | --- | --- | --- | --- | --- | --- | --- | --- | --- | --- | --- | --- | --- | --- | --- | --- | --- | --- | --- | --- | --- | --- | --- | --- | --- | --- | --- | --- | --- | --- | --- | --- | --- | --- | --- | --- | --- | --- | --- | --- | --- | --- | --- | --- | --- | --- | --- | --- | --- | --- | --- | --- | --- | --- | --- | --- | --- | --- | --- | --- | --- | --- | --- | --- | --- | --- | --- | --- | --- | --- | --- | --- | --- | --- | --- | --- | --- | --- | --- | --- | --- | --- | --- | --- | --- | --- | --- | --- | --- | --- | --- | --- | --- | --- | --- | --- | --- | --- | --- | --- | --- | --- | --- | --- | --- | --- | --- | --- | --- | --- | --- | --- | --- | --- | --- | --- | --- | --- | --- | --- | --- | --- | --- | --- | --- | --- | --- | --- | --- | --- | --- | --- | --- | --- | --- | --- | --- | --- | --- | --- | --- | --- | --- | --- | --- | --- | --- | --- | --- | --- | --- | --- | --- | --- | --- | --- | --- | --- | --- | --- | --- | --- | --- | --- | --- | --- | --- | --- | --- | --- | --- | --- | --- | --- | --- | --- | --- | --- | --- | --- | --- | --- | --- | --- | --- | --- | --- | --- | --- | --- | --- | --- | --- | --- | --- | --- | --- | --- | --- | --- | --- | --- | --- | --- | --- | --- | --- | --- | --- | --- | --- | --- | --- | --- | --- | --- | --- | --- | --- | --- | --- | --- | --- | --- | --- | --- | --- | --- | --- | --- | --- | --- | --- | --- | --- | --- | --- | --- | --- | --- | --- | --- | --- | --- | --- | --- | --- | --- | --- | --- | --- | --- | --- | --- | --- | --- | --- | --- | --- | --- | --- | --- | --- | --- | --- | --- | --- | --- | --- | --- | --- | --- | --- | --- | --- | --- | --- | --- | --- | --- | --- | --- | --- | --- | --- | --- | --- | --- | --- | --- | --- | --- | --- | --- | --- | --- | --- | --- | --- | --- | --- | --- | --- | --- | --- | --- | --- | --- | --- | --- | --- | --- | --- | --- | --- | --- | --- | --- | --- | --- | --- | --- | --- | --- | --- | --- | --- | --- | --- | --- | --- | --- | --- | --- | --- | --- | --- | --- | --- | --- | --- | --- | --- | --- | --- | --- | --- | --- | --- | --- | --- | --- | --- | --- | --- | --- | --- | --- | --- | --- | --- | --- | --- | --- | --- | --- | --- | --- | --- | --- | --- | --- | --- | --- | --- | --- | --- | --- | --- | --- | --- | --- | --- | --- | --- | --- | --- | --- | --- | --- | --- | --- | --- | --- | --- | --- | --- | --- | --- | --- | --- | --- | --- | --- | --- | --- | --- | --- | --- | --- | --- | --- | --- | --- | --- | --- | --- | --- | --- | --- | --- | --- | --- | --- | --- | --- | --- | --- | --- | --- | --- | --- | --- | --- | --- | --- | --- | --- | --- | --- | --- | --- | --- | --- | --- | --- | --- | --- | --- | --- | --- | --- | --- | --- | --- | --- | --- | --- | --- | --- | --- | --- | --- | --- | --- | --- | --- | --- | --- | --- | --- | --- | --- | --- | --- | --- | --- | --- | --- | --- | --- | --- | --- | --- | --- | --- | --- | --- | --- | --- | --- | --- | --- | --- | --- | --- | --- | --- | --- | --- | --- | --- | --- | --- | --- | --- | --- | --- | --- | --- | --- | --- | --- | --- | --- | --- | --- | --- | --- | --- | --- | --- | --- | --- | --- | --- | --- | --- | --- | --- | --- | --- | --- | --- | --- | --- | --- | --- | --- | --- | --- | --- | --- | --- | --- | --- | --- | --- | --- | --- | --- | --- | --- | --- | --- | --- | --- | --- | --- | --- | --- | --- | --- | --- | --- | --- | --- | --- | --- | --- | --- | --- | --- | --- | --- | --- | --- | --- | --- | --- | --- | --- | --- | --- | --- | --- | --- | --- | --- | --- | --- | --- | --- | --- | --- | --- | --- | --- | --- | --- | --- | --- | --- | --- | --- | --- | --- | --- | --- | --- | --- | --- | --- | --- | --- | --- | --- | --- | --- | --- | --- | --- | --- | --- | --- | --- | --- | --- | --- | --- | --- | --- | --- | --- | --- | --- | --- | --- | --- | --- | --- | --- | --- | --- | --- | --- | --- | --- | --- | --- | --- | --- | --- | --- | --- | --- | --- | --- | --- | --- | --- | --- | --- | --- | --- | --- | --- | --- | --- | --- | --- | --- | --- | --- | --- | --- | --- | --- | --- | --- | --- | --- | --- | --- | --- | --- | --- | --- | --- | --- | --- | --- | --- | --- | --- | --- | --- | --- | --- | --- | --- | --- | --- | --- | --- | --- | --- | --- | --- | --- | --- | --- | --- | --- | --- | --- | --- | --- | --- | --- | --- | --- | --- | --- | --- | --- | --- | --- | --- | --- | --- | --- | --- | --- | --- | --- | --- | --- | --- | --- | --- | --- | --- | --- | --- | --- | --- | --- | --- | --- | --- | --- | --- | --- | --- | --- | --- | --- | --- | --- | --- | --- | --- | --- | --- | --- | --- | --- | --- | --- | --- | --- | --- | --- | --- | --- | --- | --- | --- | --- | --- | --- | --- | --- | --- | --- | --- | --- | --- | --- | --- | --- | --- | --- | --- | --- | --- | --- | --- | --- | --- | --- | --- | --- | --- | --- | --- | --- | --- | --- | --- | --- | --- | --- | --- | --- | --- | --- | --- | --- | --- | --- | --- | --- | --- | --- | --- | --- | --- | --- | --- | --- | --- | --- | --- | --- | --- | --- | --- | --- | --- | --- | --- | --- | --- | --- | --- | --- | --- | --- | --- | --- | --- | --- | --- | --- | --- | --- | --- | --- | --- | --- | --- | --- | --- | --- | --- | --- | --- | --- | --- | --- | --- | --- | --- | --- | --- | --- | --- | --- | --- | --- | --- | --- | --- | --- | --- | --- | --- | --- | --- | --- | --- | --- | --- | --- | --- | --- | --- | --- | --- | --- | --- | --- | --- | --- | --- | --- | --- | --- | --- | --- | --- | --- | --- | --- | --- | --- | --- | --- | --- | --- | --- | --- | --- | --- | --- | --- | --- | --- | --- | --- | --- | --- | --- | --- | --- | --- | --- | --- | --- | --- | --- | --- | --- | --- | --- | --- | --- | --- | --- | --- | --- | --- | --- | --- | --- | --- | --- | --- | --- | --- | --- | --- | --- | --- | --- | --- | --- | --- | --- | --- | --- | --- | --- | --- | --- | --- | --- | --- | --- | --- | --- | --- | --- | --- | --- | --- | --- | --- | --- | --- | --- | --- | --- | --- | --- | --- | --- | --- | --- | --- | --- | --- | --- | --- | --- | --- | --- | --- | --- | --- | --- | --- | --- | --- | --- | --- | --- | --- | --- | --- | --- | --- | --- | --- | --- | --- | --- | --- | --- | --- | --- | --- | --- | --- | --- | --- | --- | --- | --- | --- | --- | --- | --- | --- | --- | --- | --- | --- | --- | --- | --- | --- | --- | --- | --- | --- | --- | --- | --- | --- | --- | --- | --- | --- | --- | --- | --- | --- | --- | --- | --- | --- | --- | --- | --- | --- | --- | --- | --- | --- | --- | --- | --- | --- | --- | --- | --- | --- | --- | --- | --- | --- | --- | --- | --- | --- | --- | --- | --- | --- | --- | --- | --- | --- | --- | --- | --- | --- | --- | --- | --- | --- | --- | --- | --- | --- | --- | --- | --- | --- | --- | --- | --- | --- | --- | --- | --- | --- | --- | --- | --- | --- | --- | --- | --- | --- | --- | --- | --- | --- | --- | --- | --- | --- | --- | --- | --- | --- | --- | --- | --- | --- | --- | --- | --- | --- | --- | --- | --- | --- | --- | --- | --- | --- | --- | --- | --- | --- | --- | --- | --- | --- | --- | --- | --- | --- | --- | --- | --- | --- | --- | --- | --- | --- | --- | --- | --- | --- | --- | --- | --- | --- | --- | --- | --- | --- | --- | --- | --- | --- | --- | --- | --- | --- | --- | --- | --- | --- | --- | --- | --- | --- | --- | --- | --- | --- | --- | --- | --- | --- | --- | --- | --- | --- | --- | --- | --- | --- | --- | --- | --- | --- | --- | --- | --- | --- | --- | --- | --- | --- | --- | --- | --- | --- | --- | --- | --- | --- | --- | --- | --- | --- | --- | --- | --- | --- | --- | --- | --- | --- | --- | --- | --- | --- | --- | --- | --- | --- | --- | --- | --- | --- | --- | --- | --- | --- | --- | --- | --- | --- | --- | --- | --- | --- | --- | --- | --- | --- | --- | --- | --- | --- | --- | --- | --- | --- | --- | --- | --- | --- | --- | --- | --- | --- | --- | --- | --- | --- | --- | --- | --- | --- | --- | --- | --- | --- | --- | --- | --- | --- | --- | --- | --- | --- | --- | --- | --- | --- | --- | --- | --- | --- | --- | --- | --- | --- | --- | --- | --- | --- | --- | --- | --- | --- | --- | --- | --- | --- | --- | --- | --- | --- | --- | --- | --- | --- | --- | --- | --- | --- | --- | --- | --- | --- | --- | --- | --- | --- | --- | --- | --- | --- | --- | --- | --- | --- | --- | --- | --- | --- | --- | --- | --- | --- | --- | --- | --- | --- | --- | --- | --- | --- | --- | --- | --- | --- | --- | --- | --- | --- | --- | --- | --- | --- | --- | --- | --- | --- | --- | --- | --- | --- | --- | --- | --- | --- | --- | --- | --- | --- | --- | --- | --- | --- | --- | --- | --- | --- | --- | --- | --- | --- | --- | --- | --- | --- | --- | --- | --- | --- | --- | --- | --- | --- | --- | --- | --- | --- | --- | --- | --- | --- | --- | --- | --- | --- | --- | --- | --- | --- | --- | --- | --- | --- | --- | --- | --- | --- | --- | --- | --- | --- | --- | --- | --- | --- | --- | --- | --- | --- | --- | --- | --- | --- | --- | --- | --- | --- | --- | --- | --- | --- | --- | --- | --- | --- | --- | --- | --- | --- | --- | --- | --- | --- | --- | --- | --- | --- | --- | --- | --- | --- | --- | --- | --- | --- | --- | --- | --- | --- | --- | --- | --- | --- | --- | --- | --- | --- | --- | --- | --- | --- | --- | --- | --- | --- | --- | --- | --- | --- | --- | --- | --- | --- | --- | --- | --- | --- | --- | --- | --- | --- | --- | --- | --- | --- | --- | --- | --- | --- | --- | --- | --- | --- | --- | --- | --- | --- | --- | --- | --- | --- | --- | --- | --- | --- | --- | --- | --- | --- | --- | --- | --- | --- | --- | --- | --- | --- | --- | --- | --- | --- | --- | --- | --- | --- | --- | --- | --- | --- | --- | --- | --- | --- | --- | --- | --- | --- | --- | --- | --- | --- | --- | --- | --- | --- | --- | --- | --- | --- | --- | --- | --- | --- | --- | --- | --- | --- | --- | --- | --- | --- | --- | --- | --- | --- | --- | --- | --- | --- | --- | --- | --- | --- | --- | --- | --- | --- | --- | --- | --- | --- | --- | --- | --- | --- | --- | --- | --- | --- | --- | --- | --- | --- | --- | --- | --- | --- | --- | --- | --- | --- | --- | --- | --- | --- | --- | --- | --- | --- | --- | --- | --- | --- | --- | --- | --- | --- | --- | --- | --- | --- | --- | --- | --- | --- | --- | --- | --- | --- | --- | --- | --- | --- | --- | --- | --- | --- | --- | --- | --- | --- | --- | --- | --- | --- | --- | --- | --- | --- | --- | --- | --- | --- | --- | --- | --- | --- | --- | --- | --- | --- | --- | --- | --- | --- | --- | --- | --- | --- | --- | --- | --- | --- | --- | --- | --- | --- | --- | --- | --- | --- | --- | --- | --- | --- | --- | --- | --- | --- | --- | --- | --- | --- | --- | --- | --- | --- | --- | --- | --- | --- | --- | --- | --- | --- | --- | --- | --- | --- | --- | --- | --- | --- | --- | --- | --- | --- | --- | --- | --- | --- | --- | --- | --- | --- | --- | --- | --- | --- | --- | --- | --- | --- | --- | --- | --- | --- | --- | --- | --- | --- | --- | --- | --- | --- | --- | --- | --- | --- | --- | --- | --- | --- | --- | --- | --- | --- | --- | --- | --- | --- | --- | --- | --- | --- | --- | --- | --- | --- | --- | --- | --- | --- | --- | --- | --- | --- | --- | --- | --- | --- | --- | --- | --- | --- | --- | --- | --- | --- | --- | --- | --- | --- | --- | --- | --- | --- | --- | --- | --- | --- | --- | --- | --- | --- | --- | --- | --- | --- | --- | --- | --- | --- | --- | --- | --- | --- | --- | --- | --- | --- | --- | --- | --- | --- | --- | --- | --- | --- | --- | --- | --- | --- | --- | --- | --- | --- | --- | --- | --- | --- | --- | --- | --- | --- | --- | --- | --- | --- | --- | --- | --- | --- | --- | --- | --- | --- | --- | --- | --- | --- | --- | --- | --- | --- | --- | --- | --- | --- | --- | --- | --- | --- | --- | --- | --- | --- | --- | --- | --- | --- | --- | --- | --- | --- | --- | --- | --- | --- | --- | --- | --- | --- | --- | --- | --- | --- | --- | --- | --- | --- | --- | --- | --- | --- | --- | --- | --- | --- | --- | --- | --- | --- | --- | --- | --- | --- | --- | --- | --- | --- | --- | --- | --- | --- | --- | --- | --- | --- | --- | --- | --- | --- | --- | --- | --- | --- | --- | --- | --- | --- | --- | --- | --- | --- | --- | --- | --- | --- | --- | --- | --- | --- | --- | --- | --- | --- | --- | --- | --- | --- | --- | --- | --- | --- | --- | --- | --- | --- | --- | --- | --- | --- | --- | --- | --- | --- | --- | --- | --- | --- | --- | --- | --- | --- | --- | --- | --- | --- | --- | --- | --- | --- | --- | --- | --- | --- | --- | --- | --- | --- | --- | --- | --- | --- | --- | --- | --- | --- | --- | --- | --- | --- | --- | --- | --- | --- | --- | --- | --- | --- | --- | --- | --- | --- | --- | --- | --- | --- | --- | --- | --- | --- | --- | --- | --- | --- | --- | --- | --- | --- | --- | --- | --- | --- | --- | --- | --- | --- | --- | --- | --- | --- | --- | --- | --- | --- | --- | --- | --- | --- | --- | --- | --- | --- | --- | --- | --- | --- | --- | --- | --- | --- | --- | --- | --- | --- | --- | --- | --- | --- | --- | --- | --- | --- | --- | --- | --- | --- | --- | --- | --- | --- | --- | --- | --- | --- | --- | --- | --- | --- | --- | --- | --- | --- |

**Appendix 5 - Meta-analysis methodology**

For each unique meta-analytic outcome, the meta-analysis was re-performed using the meta-analysis procedure in R (R Core Team, 2024), re-calculating the random effect pooled effect sizes using the DerSimonian Laird method (DerSimonian & Laird, 1986), weighting studies based on the inverse variance. Component studies were also sub-grouped depending on their study design, with cohort/longitudinal studies being pooled, and case control/cross sectional studies also being pooled. Heterogeneity was assessed with the I^2^ statistic, with <50% being considered low, 50-75% moderate, and >75% as high (Higgins & Thompson, 2002). Prediction intervals were also calculated. The presence of small-study effect bias (Ioannidis & Trikalinos, 2007) was also tested, which was deemed to be present in case of: a) the pooled estimate being larger than the effect size of the largest study (defined as having the smallest standard error), or b) the presence of publication bias (Egger’s regression asymmetry test p<0.10). Lastly, the excess significance bias test was conducted by evaluating whether the observed number of studies with statistically significant results was different from the expected number of studies with statistically significant results (Ioannidis & Trikalinos, 2007; Ioannidis, 2013). The expected number of studies with statistically significant results was ascertained by the sum of the power of each component study in each respective meta-analysis. Although it is impossible to ascertain true effect sizes for each meta-analytic outcome (and hence determine each component study’s power), it was assumed that the true effect size was the random effects pooled effect size. For sensitivity purposes, this was also calculated with the pooled fixed effects effect size, and the effect size of the largest component study (determine by having the lowest standard error). If the observed number of significant component studies was larger than the expected number of studies (across random, fixed, or largest study effect sizes), the differences were tested using a binominal distribution, with a conservative alpha value set at p=<0.10 to ascertain excess significance bias.

**Appendix 6 – PRISMA flow chart**


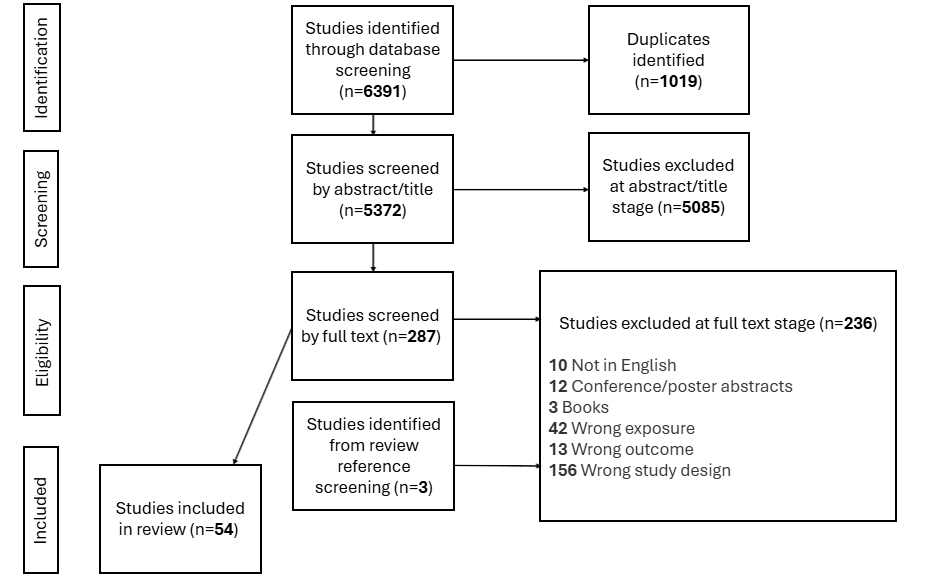


| **Appendix 7 - Full list of studies excluded at full text screening stage, with justifications** | |
| --- | --- |
| **Title** | **Reason for exclusion** |
| Association between air pollution and cognitive function in the elderly | Article in Chinese |
| Association of indoor air pollution on cognitive dysfunction among elderly | Indoor exposure |
| Education, dementia, and cerebral reserve | Article in Spanish |
| Factors Influencing Face" in People with Dementia: A Systematic Review of the Literature | Article in Chinese |
| Lifestyle-related risk factors for dementia | Article in Danish |
| Oxidative stress derived from airborne fine and ultrafine particles and the effects on brain-nervous system: part 1 | Article in Japanese |
| Risk factors for cognitive impairment | Article in Italian |
| Toxic Components of PM (2.5) and Their Toxicity Mechanisms-On the Toxicity of Sulphate and Carbon Components | Article in Japanese |
| A life-course approach to the aetiology of late-onset dementias. | Not a systematic review |
| A review of causal mechanisms underlying the link between age-related hearing loss and cognitive decline | Not a systematic review |
| A review of the possible associations between ambient PM2.5 exposures and the development of Alzheimer's disease | Not a systematic review |
| Activities outside of the care setting for people with dementia: a systematic review | No urban environment variables |
| Acute versus chronic exposures to inhaled particulate matter and neurocognitive dysfunction: Pathways to Alzheimer's disease or a related dementia. | Not a systematic review |
| Air pollution and brain health: Defining the research agenda | Not a systematic review |
| Air Pollution and Dementia: A Systematic Review and Meta-Analysis | Conference abstract |
| Air pollution and mental health: associations, mechanisms, and methods | Not a systematic review |
| Air Pollution and Noncommunicable Diseases: A Review by the Forum of International Respiratory Societies' Environmental Committee, Part 2: Air Pollution and Organ Systems | Not a systematic review |
| Air Pollution and Successful Aging: Recent Evidence and New Perspectives | Not a systematic review |
| Air Pollution and the Risk of Parkinson's Disease: A Review | Not a systematic review |
| Air pollution and your brain: what do you need to know right now. | Not a systematic review |
| Air pollution as risk factor for mental disorders: In search for a possible link with Alzheimer's disease and schizophrenia. | Not a systematic review |
| Air Pollution Neurotoxicity in the Adult Brain: Emerging Concepts from Experimental Findings | Not a systematic review |
| Air pollution, a rising environmental risk factor for cognition, neuroinflammation and neurodegeneration: The clinical impact on children and beyond. | Not a systematic review |
| Air Pollution, Combustion and Friction Derived Nanoparticles, and Alzheimer's Disease in Urban Children and Young Adults | Not a systematic review |
| Air pollution, oxidative stress, and Alzheimer's disease. | Not a systematic review |
| Air Pollution, Stress, and Allostatic Load: Linking Systemic and Central Nervous System Impacts. | Not a systematic review |
| Air Pollution, Ultrafine Particles, and Your Brain: Are Combustion Nanoparticle Emissions and Engineered Nanoparticles Causing Preventable Fatal Neurodegenerative Diseases and Common Neuropsychiatric Outcomes? | Not a systematic review |
| Air pollution: mechanisms of neuroinflammation and CNS disease | Not a systematic review |
| Air Pollution: Possible Interaction between the Immune and Nervous System? | Not a systematic review |
| Airborne Magnetite- and Iron-Rich Pollution Nanoparticles: Potential Neurotoxicants and Environmental Risk Factors for Neurodegenerative Disease, Including Alzheimer's Disease. | Not a systematic review |
| Airborne, Vehicle-Derived Fe-Bearing Nanoparticles in the Urban Environment: A Review | Not a systematic review |
| Alzheimer disease starts in childhood in polluted Metropolitan Mexico City. A major health crisis in progress. | Not a systematic review |
| Alzheimer's disease: aetiologies, pathophysiology, cognitive reserve, and treatment opportunities. | Not a systematic review |
| Alzheimer's disease: Potential preventive, non-invasive, intervention strategies in lowering the risk of cognitive decline - A review study | Not a systematic review |
| Ambient Air Pollution Increases the Risk of Cerebrovascular and Neuropsychiatric Disorders through Induction of Inflammation and Oxidative Stress. | Not a systematic review |
| Ambient Air Pollution, Noise, and Late-Life Cognitive Decline and Dementia Risk | Not a systematic review |
| Ambient particulate matter and its potential neurological consequences | Not a systematic review |
| Anthropogenic pollutants may increase the incidence of neurodegenerative disease in an aging population | Not a systematic review |
| APOE Genotype and Alzheimer's Disease: The Influence of Lifestyle and Environmental Factors | Not a systematic review |
| Apolipoprotein E4, Gender, Body Mass Index, Inflammation, Insulin Resistance, and Air Pollution Interactions: Recipe for Alzheimer's Disease Development in Mexico City Young Females | Not a systematic review |
| Apparent decreases in Swedish public health indicators after 1997-Are they due to improved diagnostics or to environmental factors? | Primary study |
| Association between ambient air pollution and Parkinson's disease: Systematic review and meta-analysis | Study is only Parkinson's disease, not specifically Parkinson’s related dementia |
| Associations and effect modification between transportation noise, self-reported response to noise and the wider determinants of health: A narrative synthesis of the literature | Outcomes do not span cognitive decline |
| Associations of Greenness, Parks, and Blue Space With Neurodegenerative Disease Hospitalizations Among Older US Adults | Primary study |
| Biological age and environmental risk factors for dementia and stroke: Molecular mechanisms | Not a systematic review |
| Biomarkers of metabolic disorders and neurobehavioral diseases in a PCB- exposed population: What we learned and the implications for future research | Not a systematic review |
| Brain Disorders and Chemical Pollutants: A Gap Junction Link? | Not a systematic review |
| Cardiovascular risk factors and incident Alzheimer disease: a systematic review of the literature | No urban environment variables |
| Cerebral consequences of environmental noise exposure | Not a systematic review |
| Chocolate, Air Pollution and Children's Neuroprotection: What Cognition Tools should be at Hand to Evaluate Interventions? | Not a systematic review |
| Climate change and neurodegenerative diseases. | Not a systematic review |
| Cognitive activity and risk of Alzheimer's disease. | Not a systematic review |
| Cognitive decline in normal aging and its prevention: A review on non-pharmacological lifestyle strategies | No urban environment variables |
| Combustion and friction-derived nanoparticles and industrial-sourced nanoparticles: The culprit of Alzheimer and Parkinson's diseases | Not a systematic review |
| Danger in the air: Air pollution and cognitive dysfunction. | Not a systematic review |
| Definitions and predictors of successful aging: a comprehensive review of larger quantitative studies. | Not a systematic review |
| Dietary Factors and Cognitive Function in Poor Urban Settings | Exposures are not urban environment |
| Driving and dementia: A review of the literature | Exposures are not urban environment |
| Early life exposure to air pollution impacts neuronal and glial cell function leading to impaired neurodevelopment. | Not a systematic review |
| Early life exposure to lead (Pb) and changes in DNA methylation: relevance to Alzheimer's disease | Not a systematic review |
| Education and Alzheimer's disease: A review of recent international epidemiological studies. | Not a systematic review |
| Education and other socioeconomic determinants of dementia and Alzheimer's disease. | Not a systematic review |
| Education, dementia, and cerebral reserve | Article in Spanish |
| Effects of air pollution on human health and practical measures for prevention in Iran | Not a systematic review |
| Effects of air pollution on the nervous system and its possible role in neurodevelopmental and neurodegenerative disorders. | Not a systematic review |
| Effects of Airborne Nanoparticles on the Nervous System: Amyloid Protein Aggregation, Neurodegeneration and Neurodegenerative Diseases. | Not a systematic review |
| Effects of low emission zones and congestion charging zones on physical health outcomes: a systematic review | Conference abstract |
| Efficacy of lifestyle and psychosocial interventions in reducing cognitive decline in older people: Systematic review | Exposures are not urban environment |
| Environment and Gene Association with Obesity and Their Impact on Neurodegenerative and Neurodevelopmental Diseases. | Not a systematic review |
| Environment, stroke, and dementia | Conference abstract |
| Environmental and dietary risk factors in Alzheimer's disease | Not a systematic review |
| Environmental and lifestyle risk factors for early-onset dementia: a systematic review | Exposures are not urban environment |
| Environmental aspects of Alzheimer's and Parkinson's diseases neuropathology’s: A focus on heavy metals and pesticides. | Book Chapter |
| Environmental Impact on the Epigenetic Mechanisms Underlying Parkinson's Disease Pathogenesis: A Narrative Review | Not a systematic review |
| Environmental Nanoparticles, SARS-CoV-2 Brain Involvement, and Potential Acceleration of Alzheimer's and Parkinson's Diseases in Young Urbanites Exposed to Air Pollution | Not a systematic review |
| Environmental neurotoxic pollutants: review | Outcomes are neurotoxicity, not cognitive decline. |
| Environmental noise-induced cardiovascular, metabolic, and mental health disorders: a brief review | Not a systematic review |
| Environmental Noise-Induced Effects on Stress Hormones, Oxidative Stress, and Vascular Dysfunction: Key Factors in the Relationship between Cerebrocardiovascular and Psychological Disorders | Not a systematic review |
| Environmental pollutants as risk factors for neurodegenerative disorders: Alzheimer and Parkinson diseases | Not a systematic review |
| Environmental pollution and mental health: A narrative review of literature | Not a systematic review |
| Environmental pollution, neurodevelopment, and cognitive impairment. | Book Chapter |
| Environmental-induced oxidative stress in neurodegenerative disorders and aging. | Not a systematic review |
| Epidemiologic Trends, Social Determinants, and Brain Health: The Role of Life Course Inequalities | Not a systematic review |
| Epidemiology and Risk Factors of Alzheimer's Disease in Iran: A Systematic Review | No urban environment variables |
| Epigenetic and neurological impairments associated with early life exposure to persistent organic pollutants | Not a systematic review |
| Examination of relationships between participation in cognitive stimulating activities and leisure activities and dementia incidence. | Dissertation |
| Exosome Production Is Key to Neuronal Endosomal Pathway Integrity in Neurodegenerative Diseases | Not a systematic review |
| Exposure of Environmental Contaminants and Development of Neurological Disorders. | Not a systematic review |
| Extracellular vesicles (exosomes and ectosomes) play key roles in the pathology of brain diseases. | Not a systematic review |
| Factors associated with cognitive decline in a population less than 65 years old. A systematic review | Article in Spanish |
| Factors Associated With Loneliness: An Umbrella Review Of Observational Studies | This is loneliness - not an urban environment exposure. |
| Fine particulate matter is a potential determinant of Alzheimer's disease: A systemic review and meta-analysis | Duplicate |
| Gene-environment interactions in Alzheimer's disease: A potential path to precision medicine | Not a systematic review |
| Genetically determined low income modifies Alzheimer's disease risk | Primary study |
| Global warming and neurological practice: Systematic review | The section on Alzheimer’s compares migrants to non-migrants - not urban environment. |
| Green spaces, dementia, and a meaningful life in the community: A mixed studies review | Not a systematic review |
| Heavy metal toxicity: Lessons from a case of simultaneous occupational exposure to manganese and mercury | Conference abstract |
| How air pollution alters brain development: The role of neuroinflammation | Not a systematic review |
| How can dementia and disability be prevented in older adults: Where are we today and where are we going? | Not a systematic review |
| How can we not 'lose it' if we still don't understand how to 'use it'? unanswered questions about the influence of activity participation on cognitive performance in older age - A mini-review | Not a systematic review |
| How does the environment affect human ageing? An interdisciplinary review | Not a systematic review |
| Human Health and Ocean Pollution. | Not a systematic review |
| Impact of air pollutants on oxidative stress in common autophagy-mediated aging diseases | Not a systematic review |
| Impact of Air Pollution and Seasonal Haze on Neurological Conditions. | Not a systematic review |
| Industrial applications of dinoflagellate phycotoxins based on their modes of action: A review | Not a systematic review |
| Inflammation, neurodegenerative diseases, and environmental exposures | Not a systematic review |
| Inflammatory effects of particulate matter air pollution | Not a systematic review |
| Influence of Woodsmoke Exposure on Molecular Mechanisms Underlying Alzheimer's Disease: Existing Literature and Gaps in Our Understanding | Not a systematic review |
| Kind streets: Urban re-design and ageing in place with cognitive impairment | Conference abstract |
| Lifelong brain health is a lifelong challenge: From evolutionary principles to empirical evidence | Not a systematic review |
| Lifestyle intervention to prevent Alzheimer's disease | Not a systematic review |
| Lifestyle interventions to prevent cognitive impairment, dementia, and Alzheimer disease | Not a systematic review |
| Lifestyle medicine - An evidence-based approach to nutrition, sleep, physical activity, and stress management on health and chronic illness | Not a systematic review |
| Mapping the complex systems that connects the urban environment to cognitive decline in adults aged 50 years and older: a group model building study | Conference abstract |
| Mild cognitive impairment: A systematic review | Not a systematic review |
| Mitigating the negative impacts of aging on cognitive function; modifiable factors associated with increasing cognitive reserve. | Outcomes do not span cognitive decline |
| Modifiable risk factors for incident dementia and cognitive impairment: An umbrella review of evidence | Duplicate |
| Modifiable risk factors for young onset dementia | Not a systematic review |
| Modifiable, Non-Modifiable, and Clinical Factors Associated with Progression of Alzheimer's Disease | Not a systematic review |
| Nature Versus Nurture: Does Proteostasis Imbalance Underlie the Genetic, Environmental, and Age-Related Risk Factors for Alzheimer's Disease? | Not a systematic review |
| Neighborhood built environment characteristics and cognition in non-demented older adults. | Dissertation |
| Neighbourhoods and dementia in the health and social care context: A realist review of the literature and implications for UK policy development | Not a systematic review |
| Neurobehavioral Consequences of Traffic-Related Air Pollution. | Not a systematic review |
| Neurotoxicity of traffic-related air pollution. | Not a systematic review |
| NLRP3 Inflammasome: A Potential Therapeutic Target in Fine Particulate Matter-Induced Neuroinflammation in Alzheimer's Disease | Not a systematic review |
| Noise as a cause of neurodegenerative disorders: molecular and cellular mechanisms. | Not a systematic review |
| Ozone and Particulate Matter Exposure and Alzheimer's Disease: A Review of Human and Animal Studies | Not a systematic review |
| Ozone Atmospheric Pollution and Alzheimer's Disease: From Epidemiological Facts to Molecular Mechanisms | Not a systematic review |
| Participation in social activities in later life: Does enjoyment have important implications for cognitive health? | Not a systematic review |
| Particulate air pollution and risk of neuropsychiatric outcomes. What we breathe, swallow, and put on our skin matters | Not a systematic review |
| Particulate matter air pollution and brain disorders: Pathophysiology | Conference abstract |
| Particulate matter and Alzheimer's disease: an intimate connection | Not a systematic review |
| Particulate matter and ultrafine particles in urban air pollution and their effect on the nervous system. | Not a systematic review |
| Perceived social isolation, evolutionary fitness, and health outcomes: a lifespan approach | Not a systematic review |
| Physical activity, air pollution and the brain | Not a systematic review |
| PM2.5 exposure in association with AD-related neuropathology and cognitive outcomes | Not a systematic review |
| Potential Impacts of Extreme Heat and Bushfires on Dementia | Not a systematic review |
| Predictors of cognitive impairment in Parkinson's disease: a systematic review and meta-analysis of prospective cohort studies | No exposures of interest |
| Predictors of conversion to dementia of probable Alzheimer type in patients with mild cognitive impairment. | Not a systematic review |
| Psychosocial protective factors in cognitive aging: A targeted review. | No exposures of interest |
| Quality of family relationships and outcomes of dementia: A systematic review | Exposures are not urban environment |
| Randomised controlled trials for the prevention of cognitive decline or dementia: A systematic review | No urban environment variables |
| Reactive oxygen species, heat stress and oxidative-induced mitochondrial damage. A review | Not a systematic review |
| RELATIONSHIP BETWEEN EDUCATION, APOLIPOPROTEIN EPSILON 4 (APOE*4) AND COGNITIVE IMPAIRMENT IN DIVERSE ETHNO-REGIONAL GROUPS: THE COSMIC COLLABORATION | Conference abstract |
| Relationship between loneliness, Psychiatric disorders, and physical health? A review on the psychological aspects of loneliness | Not a systematic review |
| Review article: Environmental heatstroke and long-term clinical neurological outcomes: A literature review of case reports and case series 2000-2016. | Not a systematic review |
| Review of urban mental health. | Book chapter |
| Risk factors for vascular disease and dementia | Not a systematic review |
| Risk of conversion from mild cognitive impairment to dementia in low- and middle-income countries: A systematic review and meta-analysis | No exposures of interest |
| Role of early life exposure and environment on neurodegeneration: Implications on brain disorders | Not a systematic review |
| Role of environmental pollutants in Alzheimer's disease: a review | Not a systematic review |
| Role of neurotoxicants in the pathogenesis of Alzheimer's disease: a mechanistic insight | Not a systematic review |
| Short review: Air pollution, noise, and lack of greenness as risk factors for Alzheimer's disease- epidemiologic and experimental evidence | Not a systematic review |
| Sleep, noise, and health: review. | Not a systematic review |
| Social capital: Implications for neurology | Not a systematic review |
| Social health, social reserve, and dementia | Not a systematic review |
| Social isolation, chronic diseases, and prevention in old age | Poster abstract |
| Social networks, social capital, and end-of-life care for people with dementia: A realist review | Not a systematic review |
| Social relationships and cognitive decline: A systematic review of longitudinal cohort studies | Duplicate |
| Socio-economic aspects of Alzheimer's disease. | Not a systematic review |
| Symposium social health: A pathway to inclusion and cognitive health Social health, social inclusion and its associations with cognitive functioning | Conference abstract |
| The Air We Breathe: Air Pollution as a Prevalent Proinflammatory Stimulus Contributing to Neurodegeneration | Not a systematic review |
| The cognitive cost of sleep lost. | Exposure (sleep) not an urban environment variable |
| The Effects of Air Pollution on the Brain: a Review of Studies Interfacing Environmental Epidemiology and Neuroimaging | Cognitive decline not assessed |
| The effects of interventions to enhance cognitive and physical functions in older people with cognitive frailty: a systematic review and meta-analysis | No urban environment variables |
| The emerging risk of exposure to air pollution on cognitive decline and Alzheimer's disease - Evidence from epidemiological and animal studies | Not a systematic review |
| The epidemiology of the dementias: An update | Not a systematic review |
| The Impact of Air Pollution on Neurodegenerative Diseases | Not a systematic review |
| The impact of air pollution to central nervous system in children and adults | Not a systematic review |
| The pathogenic effects of particulate matter on neurodegeneration: a review | Not a systematic review |
| The pollutome-connectome axis: a putative mechanism to explain pollution effects on neurodegeneration | Not a systematic review |
| The projected effect of risk factor reduction on Alzheimer's disease prevalence. | Not a systematic review |
| The proteostatic effects of traffic-derived air pollution on Alzheimer's disease risk | Not a systematic review |
| The psychological and physical effects of forests on human health: A systematic review of systematic reviews and meta-analyses | No urban environment variables |
| The role of air pollution in cognitive impairment and decline. | Not a systematic review |
| The role of traffic-related air pollution on neurodegenerative diseases in older people: An epidemiological perspective | Not a systematic review |
| Time for a Systems Biological Approach to Cognitive Aging? -A Critical Review | Not a systematic review |
| Toxic Environmental Factors and their Association with the Development of Dementia: a Mini Review on Heavy Metals and Ambient Particulate Matter. | Not a systematic review |
| Toxicant exposure and bioaccumulation: A common and potentially reversible cause of cognitive dysfunction and dementia | Not a systematic review |
| Transposable Elements and Human Diseases: Mechanisms and Implication in the Response to Environmental Pollutants | Not a systematic review |
| Use of local public transport among people with cognitive impairments-A literature review. | Not a systematic review |
| Using the natural environment to address the psychosocial impact of neurological disability: A systematic review | All variables are in people with already existing dementia |
| Water toxicants: a comprehension on their health concerns, detection, and remediation | Not a systematic review |
| What are the factors associated with physical activity (PA) participation in community dwelling adults with dementia? A systematic review of PA correlates | Individuals already have dementia |
| What is the role of modifiable environmental and lifestyle risk factors in young onset dementia? | Not a systematic review |
| **Exclusions from updated search** |  |
| A review of the literature on wellbeing and modifiable dementia risk factors | Not a systematic review |
| Choices of morbidity outcomes and concentration-response functions for health risk assessment of long-term exposure to air pollution | Not a systematic review |
| Climatic and meteorological exposure and mental and behavioral health: A systematic review and meta-analysis | Individuals already have dementia |
| Determinants of multimorbidity in low- and middle-income countries: A systematic review of longitudinal studies and discovery of evidence gaps | Outcomes do not span cognitive decline |
| Effect of nature prescriptions on cardiometabolic and mental health, and physical activity: a systematic review | Individuals already have dementia |
| Exercise effects on neuropsychiatric symptoms and quality of life in mild cognitive impairment: a systematic review and meta-analysis | No urban environmental exposure |
| Identifying exercise and cognitive intervention parameters to optimize executive function in older adults with mild cognitive impairment and dementia: a systematic review and meta-analyses of randomized controlled trials | No urban environmental exposure |
| Impact of Indoor Air Pollution on Health in Low-to-Middle Income Countries | Indoor environment |
| Influencing factors of cognitive impairment in patients with hypertension: a meta-analysis | Post-stroke cognition, article in Chinese |
| Lifetime air pollution exposure, cognitive deficits, and brain imaging outcomes: A systematic review | Outcomes do not span cognitive decline |
| Nature-based interventions for physical health conditions: A systematic review and meta-analysis | Outcomes do not span cognitive decline |
| Neighborhood infrastructure-related risk factors and non-communicable diseases: a systematic meta-review | Not a systematic review |
| Risk factors for cognitive impairment and dementia after stroke: a systematic review and meta-analysis | Post-stroke cognition |
| Structural and social determinants of health: The multi-ethnic study of atherosclerosis | Outcomes do not span cognitive decline |
| The Impact of Climate Change on Neurological Health: A Systematic Review and Meta-analysis of Observational Studies | Poster abstract |
| Urban planning, noise pollution and mental health outcomes | Poster abstract |

**Appendix 8 - JBI Appraisal Scores for Risk of Bias Assessment**

| **First author** | **Is the review question clearly and explicitly stated** | **Were the inclusion criteria appropriate for the review question** | **Was the search strategy appropriate** | **Were the sources and resources used to search for studies adequate** | **Were the criteria for appraising studies appropriate** | **Was critical appraisal conducted by two or more reviewers independently** | **Were there methods to minimize errors in data extraction** | **Were the methods used to combine studies appropriate** | **Was the likelihood of publication bias assessed** | **Were recommendations for policy and/or practice supported by the reported data** | **Were the specific directives for new research appropriate** | **Overall score** |
| --- | --- | --- | --- | --- | --- | --- | --- | --- | --- | --- | --- | --- |
| **Arora (2024)** | No | Yes | Yes | Yes | Yes | Yes | No | Yes | No | No | Yes | 7 M |
| **Béjot (2018)** | No | Yes | Yes | No | Not clear | Yes | Not clear | Yes | NA | Yes | Yes | 6 M |
| **Beydoun (2014)** | No | Yes | Yes | No | Not clear | Not clear | Not clear | Yes | Yes | No | No | 4 M |
| **Bodryzlova (2022)** | No | Yes | Yes | No | Yes | Yes | Not clear | Yes | Yes | No | Yes | 7 M |
| **Byun (2024)** | Yes | Yes | Yes | Yes | Yes | Yes | Yes | Yes | No | Yes | Yes | 10 H |
| **Caamaño-Isorna (2006)** | No | Yes | Not clear | No | Not clear | Not clear | Not clear | Yes | Yes | No | No | 3 L |
| **Carvalho (2017)** | No | Yes | No | No | Not clear | Not clear | Not clear | Yes | Not clear | Not clear | Yes | 3 L |
| **Chamberlain (2023)** | No | Yes | Yes | Yes | Yes | Yes | No | NA | Yes | Yes | Yes | 8 M |
| **Chandra (2022)** | Yes | Yes | Yes | No | Yes | Yes | Yes | Yes | NA | Yes | Yes | 9 H |
| **Chen (2022)** | No | Yes | Yes | Yes | Yes | Yes | Yes | Yes | NA | No | Yes | 8 M |
| **Cheng (2022)** | No | Yes | Yes | No | Yes | Yes | Yes | Yes | Yes | No | Yes | 8 M |
| **Clark (2020)** | No | Yes | Yes | Yes | Yes | Not clear | Not clear | Yes | NA | Yes | Yes | 7 M |
| **Clifford (2016)** | No | Yes | Yes | No | Not clear | Not clear | Not clear | Yes | NA | No | Yes | 4 M |
| **Cooper (2015)** | No | Yes | Yes | No | Yes | Yes | Not clear | Not clear | Not clear | No | Yes | 5 M |
| **Cristaldi (2022)** | No | Yes | No | No | Not clear | Not clear | Not clear | Yes | NA | No | Yes | 3 L |
| **Da (2024)** | No | Yes | Yes | Yes | Yes | Yes | Yes | Yes | Yes | Yes | Yes | 10 H |
| **de Keijzer (2016)** | No | Yes | Yes | No | Yes | Not clear | Not clear | Yes | NA | Yes | Yes | 6 M |
| **Debelu (2024)** | No | Yes | Yes | Yes | Yes | Not clear | No | Yes | NA | Yes | Yes | 7 M |
| **Dhiman (2022)** | No | Yes | No | Yes | Yes | Yes | Yes | Yes | Yes | No | Yes | 8 M |
| **Dimakakou (2018)** | No | Yes | Yes | No | Yes | Not clear | Not clear | Not clear | NA | No | Yes | 4 M |
| **El-Metwally (2019)** | No | Yes | No | No | Yes | Not clear | Not clear | Yes | NA | No | Yes | 4 M |
| **Fu (2019)** | No | Yes | No | No | Not clear | Not clear | Not clear | Not clear | NA | Yes | Yes | 3 L |
| **Fu (2020)** | No | Yes | Yes | No | Yes | No | Not clear | Yes | Yes | No | No | 5 M |
| **Gong (2023)** | No | Yes | Yes | Yes | Yes | Yes | Yes | Yes | Yes | NA | Yes | 9 H |
| **Guo (2019)** | No | Yes | Yes | No | Yes | Yes | Not clear | Not clear | Yes | No | Yes | 6 M |
| **Hamrah (2023)** | No | Yes | Yes | No | Yes | No | Not clear | Not clear | NA | No | Yes | 4 M |
| **Hegewald (2020)** | No | Yes | Yes | No | Yes | Yes | Not clear | Yes | NA | Yes | Yes | 7 M |
| **Hersi (2017)** | No | Yes | Yes | No | Yes | Yes | Yes | Yes | NA | No | Yes | 7 M |
| **Killin (2016)** | No | Not clear | No | No | Not clear | Not clear | Not clear | Not clear | NA | No | Yes | 1 L |
| **Kuiper (2015)** | No | Yes | Yes | No | Yes | Yes | Yes | Yes | Yes | No | Yes | 8 M |
| **Kuiper (2016)** | No | Yes | Yes | No | Yes | Yes | Yes | Yes | Yes | No | Yes | 8 M |
| **Lenehan (2015)** | No | Yes | No | No | No | Not clear | Not clear | Yes | NA | No | Yes | 3 L |
| **Li (2016)** | No | Yes | Yes | No | Yes | Not clear | Not clear | Yes | Yes | No | Yes | 6 M |
| **Lu (2020)** | No | Not clear | Not clear | Yes | Not clear | Not clear | Not clear | Yes | NA | No | Yes | 3 L |
| **McGrattan (2021)** | No | Yes | Yes | No | Yes | Not clear | Not clear | Yes | NA | no | Yes | 5 M |
| **Meng** (2012) | No | Yes | No | No | Yes | Not clear | Yes | Yes | Yes | No | Yes | 6 M |
| **Meo (2024)** | No | Yes | Yes | Yes | No | NA | No | Yes | Yes | Yes | No | 6 M |
| **Michael (2024)** | No | Yes | Yes | Yes | No | No | Yes | Yes | NA | Yes | Yes | 7 M |
| **Mohammadzadeh (2024)** | No | Yes | Yes | Yes | Yes | Yes | No | Yes | NA | NA | Yes | 7 M |
| **Nguyen (2023)** | No | Yes | Yes | Yes | Yes | Not clear | Yes | Yes | NA | No | Yes | 7 M |
| **Ojagbemi (2021)** | Yes | Yes | Yes | Yes | Yes | Yes | Not clear | Yes | Yes | Yes | Yes | 10 H |
| **Olayinka (2019)** | No | Yes | No | No | Yes | Not clear | Yes | Yes | NA | No | Yes | 5 M |
| **Oliveira (2024)** | No | Yes | Yes | Yes | Not clear | Not clear | Yes | Yes | NA | NA | Not clear | 5 M |
| **Peters (2019)** | No | Yes | Yes | No | Yes | Unclear | Yes | Yes | NA | Yes | Yes | 7 M |
| **Peters (2015)** | No | Yes | Yes | Yes | Not clear | Not clear | Not clear | Not clear | NA | No | Yes | 4 M |
| **Peters (2021)** | No | Yes | Yes | No | Not clear | Not clear | Not clear | Yes | NA | Yes | Yes | 5 M |
| **Plassman (2010)** | No | Yes | No | No | Yes | Yes | Not clear | Yes | NA | No | Yes | 5 M |
| **Power (2016)** | No | Yes | Yes | No | Yes | Yes | Yes | Yes | NA | No | Yes | 7 M |
| **Rodrigues (2025)** | No | Yes | Yes | Yes | Yes | Not clear | Yes | Yes | NA | Yes | Yes | 8 M |
| **Seblova (2020)** | Yes | Yes | Yes | Not clear | Yes | Not clear | Not clear | Yes | Yes | Not clear | Yes | 7 M |
| **Sharp (2011)** | No | Yes | No | No | No | Not clear | Not clear | Yes | NA | No | Yes | 3 L |
| **Song (2024)** | No | Yes | Yes | Yes | Yes | Not clear | Yes | No | NA | Yes | Yes | 7 M |
| **Tang** **(2022)** | No | Yes | No | Yes | Yes | Not clear | Not clear | Yes | NA | Yes | Yes | 6 M |
| **Thompson (2022)** | No | Yes | Yes | No | Yes | Yes | Not clear | Yes | Yes | No | Yes | 7 M |
| **Tsai (2019)** | No | Yes | Yes | No | Not clear | Not clear | Not clear | Yes | Yes | No | Yes | 5 M |
| **Tzivian (2015)** | No | Yes | Yes | No | Not clear | Not clear | Not clear | Yes | NA | Not clear | Yes | 4 M |
| **Wang (2019)** | No | Yes | Not clear | No | Yes | Yes | Not clear | Yes | Yes | Yes | Yes | 7 M |
| **Wang (2023)** | No | Yes | Yes | Yes | Not clear | Yes | Yes | Not clear | NA | Yes | Yes | 7 M |
| **Wang (2024)** | No | Yes | Yes | Yes | Yes | Yes | Not clear | Yes | Yes | No | Yes | 8 M |
| **Weuve (2021)** | No | Yes | Yes | No | No | Yes | No | No | NA | No | Yes | 4 M |
| **Wilker (2023)** | No | Yes | Yes | Yes | Yes | Yes | Yes | Yes | No | Yes | Yes | 9 H |
| **Wu (2015)** | No | Yes | No | No | Not clear | Not clear | Not clear | Not clear | NA | Yes | Yes | 3 L |
| **Xu (2015)** | No | Yes | Yes | No | Not clear | Not clear | Not clear | Yes | Yes | No | Yes | 5 M |
| **Xu (2016)** | No | Yes | Yes | No | Yes | Not clear | Not clear | Not clear | Yes | No | Yes | 5 M |
| **Xu (2024)** | No | Yes | Yes | Yes | Yes | Not clear | Yes | Yes | Yes | Not clear | Yes | 8 M |
| **Yang (202~~2~~2)** | Yes | Yes | Yes | No | Yes | Yes | Yes | Yes | NA | No | Yes | 8 M |
| **Yu (2020a)** | No | Yes | Yes | Not clear | Yes | Yes | Yes | Yes | Yes | Yes | Yes | 9 H |
| **Yu (2020b)** | No | Yes | Yes | No | Yes | Not clear | Not clear | Yes | Yes | No | No | 5 M |
| **Yuan (2024)** | No | Yes | Yes | Yes | Yes | Yes | No | Yes | NA | Not clear | Yes | 7 M |
| **Zagnoli (2022)** | No | Yes | Yes | No | Not clear | Yes | Not clear | Yes | No | No | Yes | 5 M |
| **Zhang (2024)** | No | Yes | Yes | Yes | Yes | Yes | No | Yes | Yes | Yes | Yes | 9 H |
| **Zhao (2018)** | No | Yes | Yes | No | Yes | Not clear | Not clear | Yes | Yes | Yes | Yes | 7 M |
| **Zhao (2021)** | No | Yes | Yes | No | Yes | Yes | Yes | Yes | Yes | No | Yes | 8 M |
| NA=Not applicable. L: low quality ≤3; M: moderate quality 4-8 and H: high quality ≤9. | | | | | | | | | | | | |

**Appendix 9 –** Forest plot of meta-analytical results (mixed urban design, by-products and social environment)


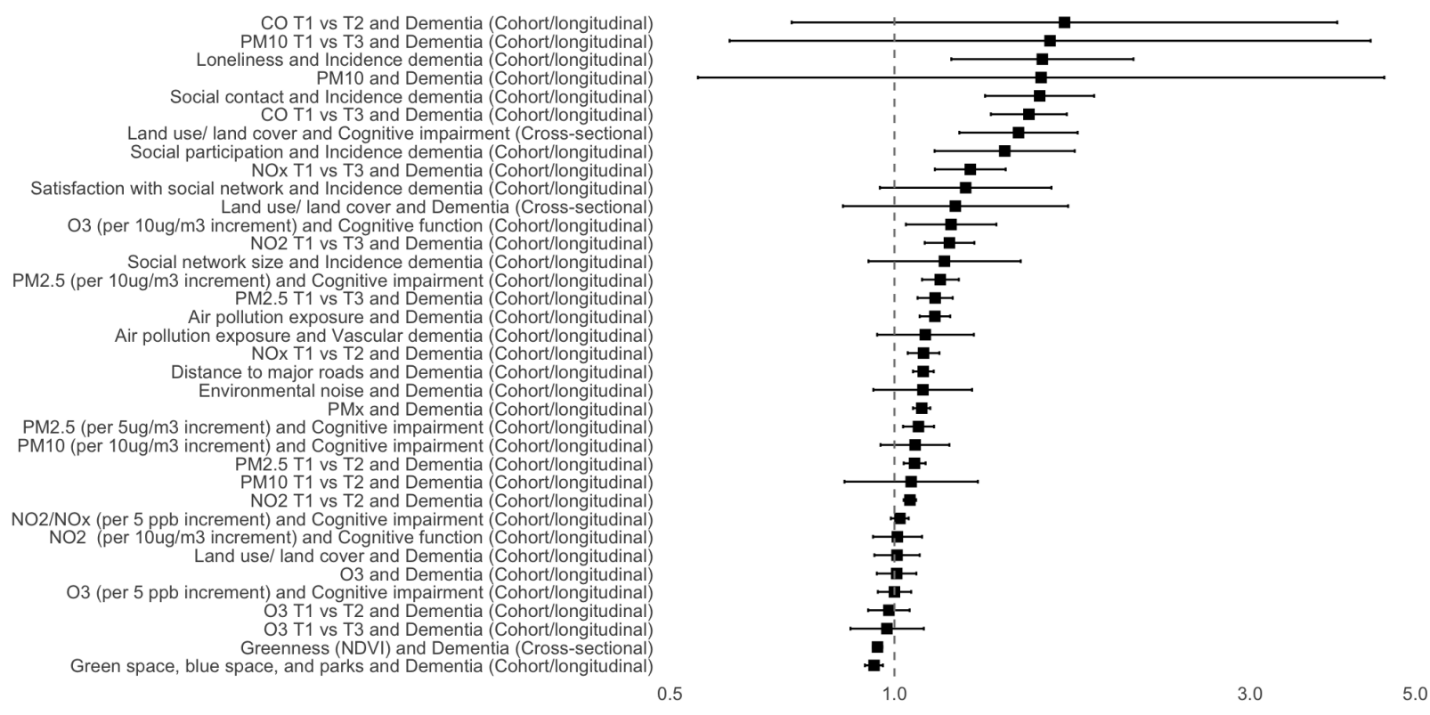


**Appendix 10 –** Forest plot of meta-analytical results (mixed urban design, by-products and social environment)


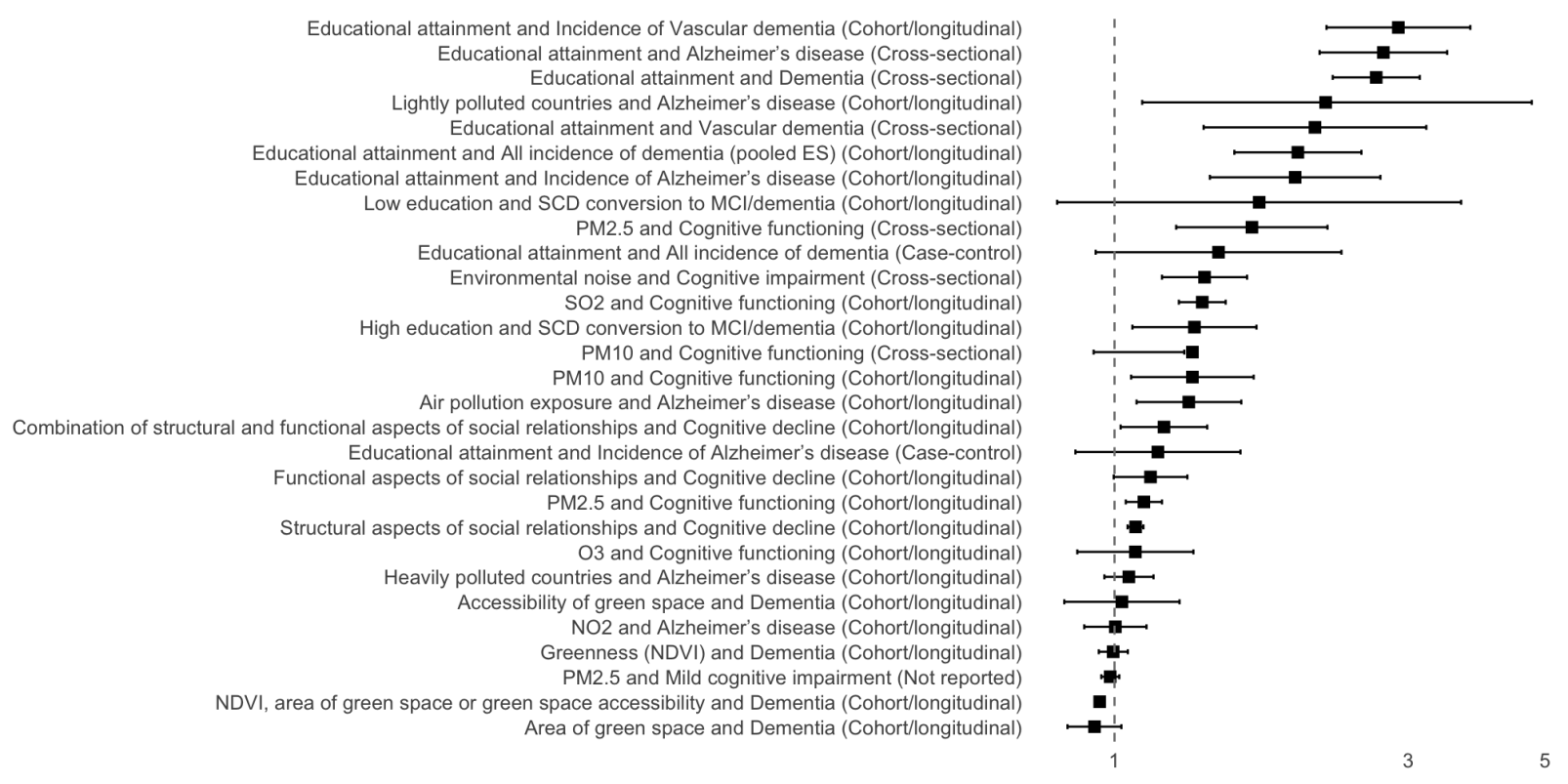


**Appendix 11 –** Forest plot of meta-analytical results (by-products)


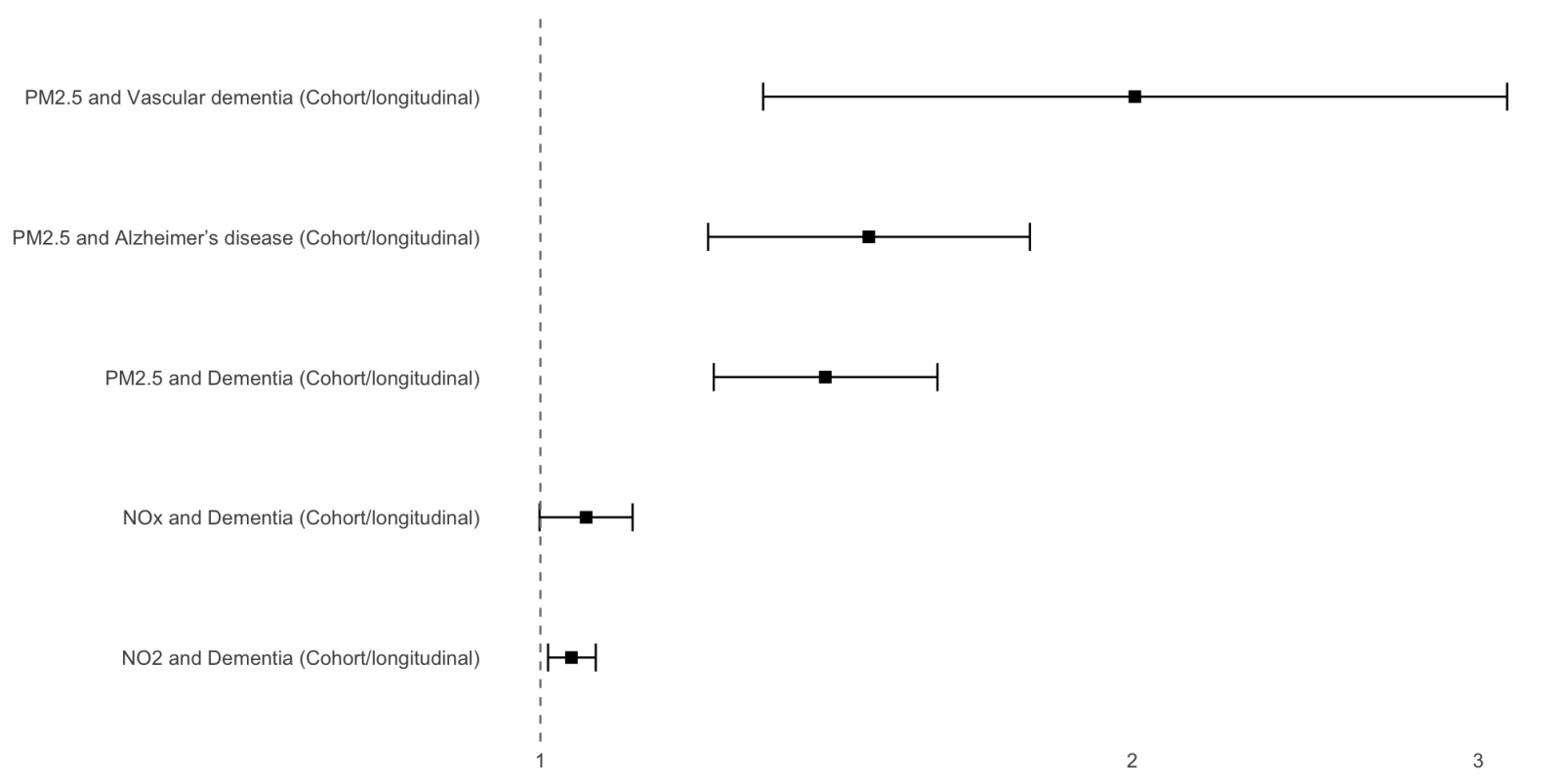


**Appendix 12 – Heat map of meta-analytical results**

| **Environmental exposure type** | **Dementia** | | **Cognitive impairment** | **Cognitive decline** | **Alzheimer's disease** | **Vascular dementia** | **Mild cognitive impairment** | **Reading and language abilities** | **Reading comprehension skills in children** |
| --- | --- | --- | --- | --- | --- | --- | --- | --- | --- |
| **Urban green and blue space** | | | | | | | | | |
| Greenness (NDVI) |  | |  |  |  |  |  |  |  |
| Cross-sectional | HP | |  |  |  |  |  |  |  |
| Cohort/longitudinal | NS | |  |  |  |  |  |  |  |
| **Land use** | | | | | | | | | |
| Land use/land cover | NS | | HP |  |  |  |  |  |  |
| **Air pollution** | | | | | | | | | |
| Air pollution exposure | HP | |  |  | SU | NS |  |  |  |
| Heavily polluted countries |  | |  |  | NS |  |  |  |  |
| Lightly polluted countries |  | |  |  | SU |  |  |  |  |
| CO T1 vs T2 | NS | |  |  |  |  |  |  |  |
| CO T1 vs T3 | HP | |  |  |  |  |  |  |  |
| NO_2_ | P | |  |  | NS |  |  |  |  |
| NO_2_ T1 vs T2 | C | |  |  |  |  |  |  |  |
| NO_2_ T1 vs T3 | HP | |  |  |  |  |  |  |  |
| NO_2_/NO_x_ (per 5 ppb increment) |  | | NS |  |  |  |  |  |  |
| NO_x_ | NS | |  |  |  |  |  |  |  |
| NO_x_ T1 vs T2 | HP | |  |  |  |  |  |  |  |
| NO_x_ T1 vs T3 | HP | |  |  |  |  |  |  |  |
| O_3_ | NS | |  |  |  |  |  |  |  |
| O_3_ (pre 5 ppb increment) |  | | NS |  |  |  |  |  |  |
| O_3_ T1 vs T2 | NS | |  |  |  |  |  |  |  |
| O_3_ T1 vs T3 | NS | |  |  |  |  |  |  |  |
| PM_10_ | NS | |  |  |  |  |  |  |  |
| PM_10_ T1 vs T2 | NS | |  |  |  |  |  |  |  |
| PM_10_ T1 vs T3 | NS | |  |  |  |  |  |  |  |
| PM_2.5_ | HP | |  |  | HP | P | NS |  |  |
| PM_2.5_(per 5ug/m3 increment) |  | | P |  |  |  |  |  |  |
| PM_2.5_T1 vs T2 | HP | |  |  |  |  |  |  |  |
| PM_2.5_T1 vs T3 | HP | |  |  |  |  |  |  |  |
| **Noise exposure** | | | | | | | | | |
| Environmental noise |  | | HP |  |  |  |  | NS |  |
| **Social relationships** | | | | | | | | | |
| Combination of structural and functional aspects of social relationships |  | |  | SU |  |  |  |  |  |
| Functional aspects of social relationships |  | |  | NS |  |  |  |  |  |
| Loneliness | P | |  |  |  |  |  |  |  |
| Satisfaction with social network | NS | |  |  |  |  |  |  |  |
| Social contact | C | |  |  |  |  |  |  |  |
| Social network size | NS | |  |  |  |  |  |  |  |
| Social participation | P | |  |  |  |  |  |  |  |
| Structural aspects of social relationships |  | |  | HP |  |  |  |  |  |
| C: convincing | |  | |  |  |  |  |  |  |
| HP: highly probable | |  | |  |  |  |  |  |  |
| P: probable | |  | |  |  |  |  |  |  |
| SU: suggestive | |  | |  |  |  |  |  |  |
| NS: not significant | |  | |  |  |  |  |  |  |
| Not assessed | |  | |  |  |  |  |  |  |

**Appendix 13 – Narrative results**

**Urban design**

**Roads, traffic density, or low emission zones**

Chamberlain et al., (2023) found no association between low emission zones and older adults diagnosis of dementia in hospitals (moderate quality) while Chen et al., (2022) suggested living in proximity to major roads is associated with poorer cognition in older adults (moderate quality). Clifford et al., (2016) included one study investigating in utero proximity to traffic density and cognitive function in children which found that near residence traffic density at birth was associated with higher scores in non-verbal intelligence but not verbal intelligence or visuomotor skills (moderate quality). Da et al., (2024) findings suggest living in proximity to roads is not statistically associated to dementia risk in older adults from meta-analysis results (RR: 1.09, 95%CI 0.94-1.27) (high quality). Dimakakou et al., (2018) included one study exploring the relationship between proximity to a busy road and cognitive function in older adults, a shorter distance to the road (10,000 cars per day) was associated with poorer cognition (moderate quality). In Peters et al., (2019) one of two included studies on distance to major roads found an association with increased dementia incidence in older adults with the exception of the 200-300m buffer (moderate quality). Song et al., (2024) also summarised that distance to major roads was associated with poorer cognition in older adults (moderate quality). Finally, Zhao et al., (2021) found that living in proximity of a road was associated with a higher risk of cognitive impairment and dementia in adults and older adults in meta-analysis (moderate quality).

**Green and blue space**

Studies included in Chen et al., (2022) suggested greenness when assessed by NDVI and park availability are associated with better cognition in older adults (moderate quality). Da et al., (2024) findings suggested green and blue spaces are associated with lower risk of dementia or better cognitive function in older adults (meta-analysis of five studies of green and blue space exposure suggests a 0.06 point reduced risk of dementia) (high quality). De Keijzer et al., (2016) findings suggested green space exposure was beneficial to children and adult cognition and some studies found exposure was beneficial or protective of older adults' cognition (moderate quality). There were conflicting/mixed findings for the relationship between green and blue spaces and cognitive function or risk of dementia in older adults in Michael et al., (2024) (moderate quality). Song et al., (2024) (moderate quality) assessed green and blue spaces and cognitive function or risk of dementia, green spaces were generally seen to have a beneficial impact on older adults' cognition. Wang et al., (2024) (moderate quality review) suggested blue space had a positive indirect effect on adults' cognitive function, while blue space was not significantly associated with older adults' risk of Alzheimer’s disease or Alzheimer’s disease related dementia. In Zagnoli et al., (2022), no significant association was found between greenness measured by NDVI and cognitive impairment or dementia in older adults in a meta-analysis (moderate quality). Zhang et al., (2024) (high quality) assessed how green space exposure was associated with dementia in older adults. Green space was protective of dementia in a random-effects meta-analysis including estimates from nine studies (OR: 0.95 95%CI 0.93-0.96) and in Zhao et al., (2021) (moderate quality) greenness was found to be significantly associated with a reduced risk of cognitive impairment and dementia for adults/older adults in cohort studies (OR: 0.96 95%CI 0.95-0.98).

**Public transport infrastructure**

Chen et al., (2022) (moderate quality) included two studies on public transport and older adults' cognitive performance which found that residing in neighbourhoods with accessible public transport was associated with slower cognitive decline over time. Micheal et al., (2024) also included two studies on public transport and the cognitive function or risk of dementia of older adults (moderate quality). Of the two studies included, one study had mixed results while the other study did not find a significant association. Song et al., (2024) (moderate quality) found that number of public transport stops was associated with better cognition in older adults.

**Walkability and cyclability related infrastructure**

Chen et al., (2022) findings suggested walkability, street connectivity, and aesthetic and pleasant neighbourhoods are associated with better cognition in older adults (moderate quality) while included studies on infrastructure for walking and cycling, road tidiness and disabled access and cognitive performance in older adults did not find an association. Michael et al., (2024) (moderate quality) found mixed evidence on the relationship between neighbourhood quality/aesthetics/disorder, walkability infrastructure and cognitive function or risk of dementia in older adults. Song et al., (2024) (moderate quality) included studies on destination accessibility, street connectivity, and walkability on older adults' cognitive function or risk of dementia. Generally, there was mixed results for destination accessibility while studies on street connectivity and walkability suggested that these features were associated with better cognition.

**Other urban design features**

Chen et al., (2022) (moderate quality) examined neighbourhood resources and neighbourhood physical disorder and cognitive performance in older adults identifying mixed findings. In Michael et al., (2024) the one included study on infrastructure and older adults' cognition had mixed findings while three of five studies on neighbourhood quality/aesthetics/disorder found significant associations, and the remaining two studies had mixed findings (moderate quality). Song et al., (2024) found that higher neighbourhood built environment quality had a beneficial impact on older adults' cognition with studies also suggesting that neighbourhood built environment diversity may have a beneficial impact on cognition (moderate quality). In Zagnoli et al., (2022) (moderate quality) a significant association was found for land use cover and increased risk of dementia in older adults but only for cohort studies (RR: 1.47 95%CI 1.22-1.76).

**Urban design by-products**

***Air pollution***

**Overall air pollution/Multiple air pollutants**

Bejot et al., (2018) synthesised the effects of air pollution on cognitive decline, Alzheimer’s disease, and vascular dementia in older adults (moderate quality). All included studies showed evidence that PM_2.5_, PM_10_, O_3_ and NO_2_ exposure was associated with cognitive decline and Alzheimer’s disease in older adults. All studies included in Bejot et al., investigating vascular dementia also showed evidence of an association with NO_2_, NO_x_ and O_3_. In Chandra et al., (2022) (high quality) prenatal exposure to air pollutants was associated with adverse effects on cognitive development in children. Of three studies on air pollution exposure in adults; two of these studies found that air pollution impaired cognitive performance. Clifford et al., (2016) (moderate quality) reported an association between postnatal air pollution exposure and poorer cognitive function in children. However, studies on air pollution exposure and adults cognitive decline yielded varying and conflicting results. Da et al., (2025) (high quality) found a strong association between long term PM_2.5_ exposure and dementia in older adults. Fu et al., (2020) (moderate quality) meta-analysis found that ,overall air pollution and heavily polluted countries were associated with Alzheimer’s disease in older adults (OR: 1.32, 95%CI 1.09-1.61 and OR: 2.2, 95%CI 1.04-4.79 respectively) while lightly polluted countries and NO_2_ exposure were not associated with Alzheimer’s disease. In Lu et al., (2020) air pollution was found to be harmful to cognitive function across all life stages (low quality). Oliveria et al., (2024) (moderate quality) included one study which found a significant association between various air pollutants and dementia in adults (statistics not reported). In Peters et al., (2015) three of six included studies on air pollution exposure and older adults cognitive impairment prevalence found a positive association while the remaining three studies had mixed results as air pollution was associated with one aspect of a cognitive test, but not other aspects of cognitive domains tested (moderate quality). Two included studies on cognitive impairment incidence also had mixed findings. In Tang et al., (2022) (moderate quality) only one of three studies found that overall air pollution exposure was associated with vascular dementia while nine of twelve studies found an association between air pollution and dementia risk. In Tzivian et al., (2015) (moderate quality) several included studies reported associations between cognitive performance in older adults and air pollution. Included studies also reported that higher exposure to air pollution was associated with Alzheimer’s like pathology.

**PM_2.5_**

Abolhasani et al., (2023) (high quality) synthesised the effects of PM_2.5_ on the risk of dementia, Alzheimer’s disease, and non-Alzheimer's disease dementia in older adults. The hazard ratio of dementia per 1ug/m3 increase in PM_2.5_ was significant (HR: 1.02, 95%CI 1.01-1.03) while the hazard ratios for Alzheimer’s disease and non-Alzheimer's disease dementia were not significant. Cheng et al., (2022) found significant hazard ratios between PM_2.5_ and vascular dementia, dementia, and Alzheimer’s disease in older adults in meta-analysis (moderate quality). In Clifford et al., (2016) (moderate quality) in utero PM_2.5_ exposure was associated with reduced cognitive function for children in one of three studies. Cristaldi et al., (2022) included studies which suggested a positive association between PM_2.5_ and Alzheimer’s disease, and between PM_2.5_ and vascular dementia in older adults (low quality). In Dhiman et al., (2022) (moderate quality) the pooled hazard ratios for PM_2.5_ and all-cause dementia was significant (HR: 1.03 95%CI 1.01-1.06) and the pooled hazard ratio for PM_2.5_ and Alzheimer’s disease was significant (HR: 1.08, 95%CI 1.01-1.15). However, the pooled hazard ratio for PM_2.5_ and vascular dementia was not significant. In addition, in Dhiman et al., PM_2.5_ was associated with cognitive decline in older adults in all twelve studies which investigated this outcome. In Fu et al., (2019), the only significant meta-analysis finding was between PM_2.5_ and dementia in older adults (OR: 1.16, 95%CI 1.07-1.26)., mild cognitive impairment and dementia outcomes were not significant (low quality). Gong et al., (2023) (high quality) meta-analysis results identified a significantly higher odds ratio of all-cause dementia and Alzheimer’s disease from 10ug/m3 growth in PM_2.5_ exposure while the odds ratio for vascular dementia was not significant. Meta-analysis also identified PM_2.5_ was associated with lower cognitive function measured by MMSE and neuropsychological tests but not TICS. In Meo et al., (2024) (moderate quality) PM_2.5_ exposure was associated with cognitive impairment in older adults in the meta-analysis of cohort studies but not cross-sectional studies (pooled OR for cohort studies: 1.35, 95%CI 1.02-1.79). In Mohammadzadeh et al., (2024) (moderate quality) the majority of included studies found that PM_2.5_ exposure increased the risk of Alzheimer’s dementia or non-Alzheimer's disease dementia in older adults. Similarly in Oliveira et al., (2024) most studies found that higher PM_2.5_ exposure was associated with an increased risk of dementia in adults (moderate quality). In Peters et al., (2019) (moderate quality) and Peters et al., (2021) (moderate quality) again the majority of studies on PM_2.5_ exposure found a significant association with dementia incidence in older adults. However, in Peters et al., (2021) most studies on PM_2.5_ and cognitive decline were not significant. Power et al., (2016) reported mixed findings from included studies on PM_2.5_ and cognitive decline in adults and older adults and no association was found for dementia incidence (moderate quality). Tang et al., (2022) (moderate quality) observed a significant dose response relationship for PM_2.5_ and dementia in adults and all included studies in Tsai et al., (2019) (moderate quality) found a significant association between PM_2.5_ exposure and dementia or Alzheimer's disease risk in older adults. Weuve et al., (2021) (moderate quality) included studies on PM_2.5_ and cognitive performance in adults and older adults with mixed and inconsistent results. Xu et al., (2024) meta-analysis for PM_2.5_ and decreased cognitive function was significant (RR: 1.17, 95%CI 1.09-1.26) (moderate quality) and meta-analysis for PM_2.5_ and cognitive test scores was also significant, PM_2.5_ was associated with a 0.3 point reduction in score. In Yang et al., (2022) (moderate quality) no association was found between PM_2.5_ and Alzheimer’s disease in older adults in one included study however, both included studies of cognitive impairment were significant. Yu et al., (2020) found no significant association for PM_2.5_ exposure and cognitive impairment in adults in pooled analysis (high quality). In Yuan et al., (2024) (moderate quality) there was mixed findings for PM_2.5_ and cognitive function in children while PM_2.5_ exposure was associated with lower cognitive function in two of three studies of adults and older adults. Zhao et al., (2021) (moderate quality) results showed significant associations for PM_2.5_ and vascular dementia however, no significant associations were found for PM_2.5_ exposure and Alzheimer’s disease for adults and older adults.

**PM_10_**

In Clifford et al., (2016) (moderate quality) there were no significant findings regarding in utero PM_10_ and cognitive function in children. Gong et al., (2023) (high quality) conducted a meta-analysis on PM_10_ exposure, identifying a significant odds ratio for PM_10_ and vascular dementia in adults/older adults but not for all-cause dementia or Alzheimer’s disease. Gong et al., also found that PM_10_ exposure was associated with lower cognitive function when assessed with neuropsychological testing but not the MMSE. Meo et al., (2024) (moderate quality) meta-analysis of PM_10_ exposure and risk of cognitive impairment in older adults was not significantly significant. In Mohammadzadeh et al., (2024) (moderate quality) the majority of included studies found that PM_10_ exposure increased the risk of Alzheimer’s dementia and non-Alzheimer's dementia in older adults and in Peters et al., (2019) (moderate quality) PM_10_ was associated with older adults' memory decline in one included study. In Power et al., (2016) results from included studies on PM_10_ were mixed and conflicting for cognitive impairment, dementia and cognitive levels of adults and older adults (moderate quality). Tang et al., (2022) (moderate quality) observed no significant dose response relationship for PM_10_ and dementia in adults. Xu et al., (2024) (moderate quality) meta-analysis for PM_10_ and decreased cognitive function was not significant while meta-analysis for and cognitive test scores was significant, PM_10_ being associated with a 0.15 point reduction in score. Finally, no significant associations were found for PM_10_ and cognitive impairment, dementia or vascular dementia in adults and older adults in Zhao et al., (2021) (moderate quality).

**PM/PM_x_**

In Chandra et al., (2022) prenatal exposure to PM was associated with adverse effects on brain volume and was also determined to have an adverse effect on cognitive impairment in adults and older adults (high quality). Da et al., (2025) meta-analysis identified that PM_x_ was associated with higher risk ratio of dementia (RR: 1.09, 95%CI 1.06-1.12) (high quality review). Killin et al., (2016) (low quality) found ‘strong’ evidence of a positive association between PM and dementia from two included studies.

**NO_2_ and NO_x_**

In Abolhasani et al., (2023) (high quality) NO_x_ was not associated with dementia in older adults (HR: 1.14; 95%CI 0.99-1.31) while NO_2_ was associated with increased hazard ratio of dementia (HR: 1.06, 95%CI 1.01-1.11) but not Alzheimer’s disease. Clifford et al., (2016) (moderate quality) found that one of three studies of in utero NO_2_ exposure was associated with reduced cognitive function in children. Da et al., (2025) (high quality) meta-analysis found that NO_x_ exposure was associated with a significantly higher risk ratio of dementia in older adults while Dhiman et al., (2022) (moderate quality) pooled hazard ratios for NO_2_ and NO_x_ and all-cause dementia were not significant. Fu et al., (2020) (moderate study) a meta-analysis on NO_2_ exposure and Alzheimer’s disease in older adults was not significant. Killin et al., (2016) (low quality) found ‘strong’ evidence of a positive association between NO_x_ and dementia in older adults from two studies. In Mohammadzadeh et al., (2024) (moderate quality) the majority of included studies found that NO_x_ and NO_2_ exposure increased the risk of Alzheimer’s dementia and non-Alzheimer's disease dementia in older adults. In Oliveira et al., (2024) (moderate quality) twelve studies were included which assessed NO_x_ exposure, six found NO_x_ was associated with higher risk of dementia in adults while two studies found NO_x_ was associated with lower risk of dementia. In Peters et al., (2019) (moderate quality) the majority of studies on NO_x_ or NO_2_ exposure found a significant association with dementia incidence in older adults and Tang et al., (2022) (moderate quality) observed significant dose response relationships for NO_2_ and NO_x_ and dementia in adults. Weuve et al., (2021) (moderate quality) investigated NO_2_ and NO_x_, cognitive performance, and cognitive impairment incidence. Results from included studies were mixed and inconsistent. In Wilker et al., (2023) the majority of included studies reported significant associations for NO_x_ and NO_2_ and dementia in older adults however, the pooled effect size was not significant (high quality). Xu et al., (2024) meta-analysis for NO_2_ exposure and decreased cognitive function or cognitive test scores was not significant (moderate quality) and despite some included studies in Yu et al., (2020) reporting significant associations, Yu et al., found no significant associations between NO_2_/NO_x_ exposure and cognitive impairment in adults in pooled analysis (high quality). In Yuan et al., (2024) (moderate quality) NO_2_ was associated with lower cognitive function in children in one study and in Zhao et al., (2021) (moderate quality) significant associations were identified between NO_2_ and dementia and vascular dementia in adults/older adults while no significant associations were found for NO_2_ or NO_x_ and Alzheimer's disease.

**SO_2_**

Clifford et al., (2016) found that in utero SO_2_ exposure was associated with reduced fine motor skills in children (moderate quality). In Dhiman et al., (2022) (moderate quality) SO_2_ was not significantly associated with vascular dementia in older adults. In Meo et al., (2024) SO_2_ was associated with higher risk of cognitive impairment in older adults (OR: 1.39, 95%CI 1.27-1.51) while results from Xu et al., (2014) meta-analysis for SO_2_ and cognitive test scores was not significant (moderate quality). Zhao et al., (2021) (moderate quality) included studies which found significant associations for SO_2_, cognitive impairment and dementia in older adults.

**Black carbon or elemental** **carbon**

Mohammadzadeh et al., (2024) (moderate quality) concluded that there was insufficient information to comment on the relationship between black carbon and (non-) Alzheimer’s disease dementia in older adults. No studies on black carbon in Oliveira et al., (2024) identified an association with dementia in adults (moderate quality) while one study in Yuan et al., (2024) (moderate quality) reported that elemental carbon was associated with lower cognitive function in children.

**CO**

In Clifford et al., (2016) (moderate quality) there were no significant findings identified for CO exposure and children's cognitive function from one included study. Studies included in Dhiman et al., (2022) (moderate quality) did not find a significant relationship between CO and vascular dementia or all-cause dementia in older adults. Killin et al., (2016) found ‘moderate’ evidence of a positive association between CO and dementia in older adults from one included study (low quality). In Mohammadzadeh et al., (2024), CO was associated with Alzheimer’s dementia and non-Alzheimer's dementia in older adults (moderate quality) and Tang et al., (2022) (moderate quality) observed a significant dose response relationship for CO and dementia in older adults. Finally, in Zhao et al., (2021) (moderate quality) CO was associated with cognitive impairment and dementia in adults and older adults.

**O_3_**

In Abolhasani et al., (2023) (high quality) O_3_ was associated with Alzheimer’s disease but not dementia in older adults. Clifford et al., (2016) (moderate quality) identified no significant findings regarding in utero O_3_ exposure and cognitive function in children however, O_3_ was associated with poorer cognitive function in adults. Dhiman et al., (2022) (moderate quality) meta-analysis for O_3_ and Alzheimer’s disease was not significant while Killin et al., (2016) (low quality) found ‘strong’ evidence of a positive association between O_3_ and dementia in older adults from two studies. In Meo et al., (2024) (moderate quality) O_3_ was not associated with cognitive impairment in older adults in meta-analysis. Mohammadzadeh et al., (2024) (moderate quality) found O_3_ exposure may increase the risk of Alzheimer’s dementia and non-Alzheimer's dementia in older adults and in Peters et al., (2019) (moderate quality) some included studies found significant associations between O_3_ and cognitive decline, dementia or Alzheimer’s disease in older adults however, the direction of these associations was inconsistent. In Power et al., (2016), O_3_ was associated with greater risk of dementia-related outcomes in adults/older adults in two of three included studies (moderate quality). Tang et al., (2022) (moderate quality) found no significant dose response relationship for O_3_ and dementia in adults and Weuve et al., (2021) (moderate quality) results for cognitive decline in adults and older adults were mixed and inconsistent. Xu et al., (2024) meta-analysis for O_3_ exposure and decreased cognitive function was significant (RR: 1.19, 95%CI 1.04-1.37) while meta-analysis for O_3_ exposure and cognitive test scores was not significant (moderate quality). Despite some included studies reporting significant associations, Yu et al., (2020) found no significant associations between O_3_ exposure and cognitive impairment in adults within pooled analysis (high quality). Zhao et al., (2021) (moderate quality) included studies which found significant associations for O_3_ and cognitive impairment and dementia in adults and older adults. Finally, in Zhao et al., (2018) both included studies on O_3_ and cognitive impairment in older adults found a significant association and four out of five studies on dementia found a significant association.

***Noise pollution***

**Environmental noise pollution**

Clark et al., (2020) review concluded there was ‘low quality evidence’ of no significant effect of noise exposure on vascular dementia, and ‘very low quality evidence’ of an adverse effect of noise exposure on dementia symptoms in adults/older adults (moderate quality). In Da et al., (2024) noise was not significantly associated with dementia risk in older adults in a random-effect model (RR: 1.09, 95%CI 0.94-1.27). Killin et al., (2016) found 'moderate' evidence of negative association between excessive noise and Alzheimer’s disease, but only in one study (low quality). In Thompson et al., (2022) (moderate quality) one of three included studies found higher odds of cognitive impairment in adults from higher compared to lower noise exposure while environmental noise did not have an impact on reading or language abilities in children. In Zhao et al., (2021) (moderate quality) noise exposure was associated with cognitive impairment in adults and older adults in cohort studies.

**Traffic-related noise pollution**

Dimakakou et al., (2018) (moderate quality) found that long term road traffic noise was associated with higher likelihood of mild cognitive impairment in adults and older adults in one study. Hegewald et al., (2020) (moderate quality) found no evidence of a relationship between road traffic noise and dementia in older adults. However, one included study in Hegewald et al., found a higher odds ratio of mild cognitive impairment correlated to noise levels of 60 dB Lden or more (OR: 1.4, 95%CI 1.03-1.91) and Lnight levels of 55 dB or more (OR: 1.8, 95%CI 1.07-3.04). One included study also found that road traffic noise had an impact on cognitive function for Lden but not for Lnight (statistics NR). Tzivian et al., (2015) included one study on noise exposure and cognitive performance in adults which found that police officers from high traffic areas had worse cognitive performance compared to office workers (moderate quality).

**Temperature**

Byun et al., (2024) (high quality) reported that higher temperature (1 degree Celsius higher ambient temperature) and heat waves were consistently associated with hospital admissions or mortality due to dementia in older adults. Oliveira et al., (2024) included studies on temperature and dementia in adults (moderate quality) with no significant associations being identified. Similarly, Zhao et al., (2021) (moderate quality) assessed associations between temperature and cognitive impairment and dementia in adults and older adults and no significant associations were found for exposure to higher temperature.

**Chemicals and solvents**

Chandra et al., (2022) (high quality) found that prenatal exposure to isophorene was associated with lower mathematical performance in children and prenatal exposure to persistent organic pollutants and polycyclic aromatic hydrocarbons were associated with adverse effects in terms of cognitive development. Additionally, postnatal exposure to persistent organic pollutants was associated with adverse effects on children's cognitive development and postnatal exposure to isophorene was associated with lower mathematical performance, similar to prenatal exposure. Chandra et al., also included one study which provided evidence that polyaromatic hydrocarbons were associated with a decline in cognitive performance in older adults. In Clifford et al., (2016) (moderate quality) in utero exposure to polycyclic aromatic hydrocarbons was associated with reduced intelligence in children at 3 and 5 years of age but not 1 and 2 years of age while there were no significant findings identified regarding in utero benzene exposure. Mohammadzadeh et al., (2024) (moderate quality) reported on one study which found that prior exposure to solvents (such as benzene) was associated with an increased risk of Alzheimer’s dementia in older adults.

**Pesticides and heavy metals**

Olayinka et al., (2019) (moderate quality) included studies on pesticide, lead and mercury exposure and Alzheimer’s disease in older adults. Three of six studies on pesticide exposure found significant associations while there were no significant associations found between occupational exposure to lead or mercury.

**Traffic-related exposures**

Chandra et al., (2022) (high quality) found that prenatal exposure to traffic-related air pollution was associated with adverse effects on intelligence and brain volume in children and postnatal exposure to traffic-related air pollution impaired cognitive performance. In Debelu et al., (2024) (moderate quality) higher traffic-related PM_2.5_ exposure was associated with poorer cognitive function in one study of adults while traffic-related NO_x_ exposure was not significantly associated with hazard ratio of dementia diagnosis. Debelu et al., also found that traffic-related elemental carbon and NO_2_ was associated with substantial smaller growth in cognitive measurements in children (statistics not reported). Dimakakou et al., (2018) (moderate quality) included studies assessing associations between traffic-related air pollution and dementia in adults and older adults. The findings supported an association between traffic-related air pollution and dementia. Killin et al., (2016) (low quality) found ‘moderate’ evidence of no association between diesel motor exhaust and non-vascular dementia based on one study of older adults. Included studies in Power et al., (2016) found that traffic related NO_2_ or NO_x_ was associated with an adverse effect on cognitive impairment in adults and older adults (moderate quality). Finally, Yuan et al., (2024) found that long term PM_10_ and NO_2_ exposure was significantly associated with increased risk of vascular dementia in one study of adults and older adults (moderate quality).

**Social environment**

**Socio-economic status and deprivation**

McGrattan et al., (2021) (moderate quality) included studies which assessed associations between socio-economic status and mild cognitive impairment in older adults reporting that low socio-economic status is a risk factor for mild cognitive impairment. In Michael et al., (2024) (moderate quality) neighbourhood socio-economic status had mixed findings with regards to cognition in older adults with three studies suggesting a significant relationship, three studies having mixed results and three studies finding no significant relationship. Rodrigues et al., (2025) found that higher income status was associated with a lower risk of cognitive decline in older adults (moderate quality) similar to Wang et al., (2023) who reported a significant relationship was between socio-economic status and cognitive impairment, all-cause dementia and Alzheimer’s disease in older adults (moderate quality). Wu et al., (2015) included studies on area deprivation and cognitive function in older adults (low quality review). Local deprivation was associated with poorer cognitive function, cognitive decline, or a higher risk of cognitive impairment in eight of eleven included studies. Zhao et al., (2021) (moderate quality) also found significant associations between socio-economic status and Alzheimer’s disease, cognitive impairment and dementia in adults and older adults.

**Social contact, social relationships, and social support**

Kuiper et al., (2016) (moderate quality) included studies on functional and structural aspects of social relationships and cognitive decline in older adults. Included studies were more likely to find a significant association if they investigated a combination of functional and structural aspects of social relationships as opposed to functional or structural aspects independently. Kuiper et al., (2015) (moderate quality) pooled analysis for less social contact and social participation and dementia incidence in older adults was significant, while the pooled analysis for social network satisfaction or social network size was not significant. McGrattan et al., (2021) reported that social contact is a protective factor for mild cognitive impairment in older adults (moderate quality) and in Plassman et al., (2010) included studies on social engagement and cognitive decline in adults and older adults and reported there was no consistent association (moderate quality). In Rodrigues et al., (2025) (moderate quality) all studies on civic engagement (volunteering) with >11 years of follow-up found this was associated with reduced cognitive decline in older adults as well as the majority of studies on social exclusion with >11 year follow up. Social exclusion was seen to increase the risk of cognitive impairment by 80% in one study (OR: 1.8, p<0.001) included in Rodrigues et al., (2025). Zhao et al., (2021) (moderate quality) included studies on community engagement, community groups, overall social contact, social contact with friends, social contact with family. Community engagement and community groups were reported to be protective factors of cognitive impairment and dementia, and all social contact variables were found to be protective of dementia and Alzheimer’s disease in adults and older adults.

**Social isolation and loneliness**

Hamrah et al., (2023) (moderate quality) included studies on social isolation and dementia in adults and older adults and reported mixed results. Kuiper et al., (2015) (moderate quality) pooled analysis for loneliness and dementia incidence in older adults was significant.

**Traffic safety**

Chen et al., (2022) included one study on traffic safety and cognitive performance in older adults (moderate quality). Traffic safety was not significantly associated with cognitive performance.

| **Appendix 14 - JBI Risk of Bias Assessment per specific environment exposure** | | | |
| --- | --- | --- | --- |
| **Environmental exposure** | **Number of individual results of low quality** | **Number of individual results of moderate quality** | **Number of individual results of high quality** |
| **Urban design** | | | |
| **Roads, traffic density or low emission zones** | **N=0/8** | **N=7/8** | **N=1/8** |
| **Green and blue space** | **N=0/16** | **N=14/16** | **N=2/16** |
| **Public transport infrastructure** | **N=0/3** | **N=3/3** | **N=0/3** |
| **Walkability and cyclability infrastructure** | **N=0/8** | **N=8/8** | **N=0/8** |
| **Other** | **N=0/9** | **N=9/9** | **N=0/9** |
| **Urban design by-products** | | | |
| **Overall air pollution** | **N=1/18** | **N=14/18** | **N=3/18** |
| **PM_2.5_** | **N=5/48** | **N=33/48** | **N=10/48** |
| **PM_10_** | **N=0/20** | **N=14/20** | **N=6/20** |
| **PM/PM_x_** | **N=0/4** | **N=1/4** | **N=3/4** |
| **NO_2_ and NO_x_** | **N=1/29** | **N=21/29** | **N=7/29** |
| **SO_2_** | **N=0/5** | **N=5/5** | **N=0/5** |
| **Black carbon and elemental carbon** | **N=0/4** | **N=4/4** | **N=0/4** |
| **CO** | **N=1/9** | **N=8/9** | **M=0/9** |
| **O_3_** | **N=1/20** | **N=16/20** | **N=3/20** |
| **Traffic-related air pollution** | **N=1/10** | **N=7/10** | **N=2/10** |
| **Environmental noise** | **N=1/8** | **N=6/8** | **N=1/8** |
| **Traffic-related noise** | **N=0/5** | **N=5/5** | **N=0/5** |
| **Temperature** | **N=0/5** | **N=2/5** | **M=3/5** |
| **Chemicals and solvents** | **N=0/9** | **N=3/9** | **N=6/9** |
| **Pesticide and heavy metals** | **N=0/2** | **N=2/2** | **N=0/2** |
| **Social environment** | | | |
| **Socio-economic status and deprivation** | **N=1/9** | **N=8/9** | **N=0/9** |
| **Social contact, social relationships and social support** | **N=0/18** | **N=18/18** | **N=0/18** |
| **Social isolation and loneliness** | **N=0/2** | **N=2/2** | **N=0/2** |
| **Traffic safety** | **N=0/1** | **N=1/1** | **N=0/1** |

[1] Chamberlain RC, Fecht D, Davies B, Laverty AA. Health effects of low emission and congestion charging zones: a systematic review*. The Lancet Public Health* 2023;8:e559–74.

[2] Chen X, Lee C, Huang H. Neighborhood built environment associated with cognition and dementia risk among older adults: A systematic literature review*. Soc Sci Med* 2022;292:114560.

[3] Clifford A, Lang L, Chen R, Anstey KJ, Seaton A. Exposure to air pollution and cognitive functioning across the life course – A systematic literature review*. Environ Res* 2016;147:383–98.

[4] Da L, Song X, Jia Z, Myers NGL, Sun J, Wei J *et al*. Objectively measured environmental features and their association with cognition and dementia: A systematic review and meta-analysis*. Ageing Research Reviews* 2025;104:102630.

[5] Dimakakou E, Johnston HJ, Streftaris G, Cherrie JW. Exposure to Environmental and Occupational Particulate Air Pollution as a Potential Contributor to Neurodegeneration and Diabetes: A Systematic Review of Epidemiological Research*. Int J Environ Res Public Health* 2018;15:1704. doi: 10.3390/ijerph15081704.

[6] Peters R, Ee N, Peters J, Booth A, Mudway I, Anstey KJ. Air Pollution and Dementia: A Systematic Review*. Journal of Alzheimer’s Disease* 2019;70:S145–63.

[7] Song Y, Liu Y, Bai X, Yu H. Effects of neighborhood built environment on cognitive function in older adults: a systematic review*. BMC Geriatrics* 2024;24:194.

[8] Zhao Y, Qu Y, Ou Y, Zhang Y, Tan L, Yu J. Environmental factors and risks of cognitive impairment and dementia: A systematic review and meta-analysis*. Ageing Research Reviews* 2021;72:101504.

[9] de Keijzer C, Gascon M, Nieuwenhuijsen MJ, Dadvand P. Long-Term Green Space Exposure and Cognition Across the Life Course: a Systematic Review*. Current Environmental Health Reports* 2016;3:468–77.

[10] Michael Y, Senerat A, Buxbaum C, Ezeanyagu U, Hughes T, Hayden K *et al*. Systematic Review of Longitudinal Evidence and Methodologies for Research on Neighborhood Characteristics and Brain Health*. Public Health Rev* 2024;45:1606677.

[11] Wang L, Md Sani N. The impact of outdoor blue spaces on the health of the elderly: A systematic review*. Health Place* 2024;85:103168.

[12] Zagnoli F, Filippini T, Jimenez MP, Wise LA, Hatch EE, Vinceti M. Is Greenness Associated with Dementia? A Systematic Review and Dose–Response Meta-analysis*. Current Environmental Health Reports* 2022;9:574–90.

[13] Zhang Y, Wu T, Yu H, Fu J, Xu J, Liu L *et al*. Green spaces exposure and the risk of common psychiatric disorders: A meta-analysis*. SSM - Population Health* 2024;25:101630.

[14] Béjot Y, Reis J, Giroud M, Feigin V. A review of epidemiological research on stroke and dementia and exposure to air pollution*. Int J Stroke* 2018;13:687–95.

[15] Chandra M, Rai C, Kumari N, Sandhu VK, Chandra K, Krishna M *et al*. Air Pollution and Cognitive Impairment across the Life Course in Humans: A Systematic Review with Specific Focus on Income Level of Study Area*. International Journal of Environmental Research and Public Health* 2022;19:1405.

[16] Fu P, Yung K. Air Pollution and Alzheimer’s Disease: A Systematic Review and Meta-Analysis*. Journal of Alzheimer’s Disease* 2020;77:701–14.

[17] Lu JG. Air pollution: A systematic review of its psychological, economic, and social effects*. Current Opinion in Psychology* 2020;32:52–65.

[18] Oliveira M, Padrão A, Teodoro AC, Freitas A, Gonçalves H. Geospatial analysis of environmental atmospheric risk factors in neurodegenerative diseases: a systematic review update*. Syst Rev* 2024;13:267–7.

[19] Peters R, Peters J, Booth A, Mudway I. Is air pollution associated with increased risk of cognitive decline? A systematic review*. Age Ageing* 2015;44:755–60.

[20] Tang J, Chen A, He F, Shipley M, Nevill A, Coe H *et al*. Association of air pollution with dementia: a systematic review with meta-analysis including new cohort data from China*. Environ Res* 2023;223:115048.

[21] Tzivian L, Winkler A, Dlugaj M, Schikowski T, Vossoughi M, Fuks K *et al*. Effect of long-term outdoor air pollution and noise on cognitive and psychological functions in adults*. Int J Hyg Environ Health* 2015;218:1–11.

[22] Abolhasani E, Hachinski V, Ghazaleh N, Azarpazhooh MR, Mokhber N, Martin J. Air Pollution and Incidence of Dementia: A Systematic Review and Meta-analysis*. Neurology* 2023;100:e242–54.

[23] Cheng S, Jin Y, Dou Y, Zhao Y, Duan Y, Pei H *et al*. Long-term particulate matter 2.5 exposure and dementia: a systematic review and meta-analysis*. Public Health* 2022;212:33–41.

[24] Cristaldi A, Fiore M, Oliveri Conti G, Pulvirenti E, Favara C, Grasso A *et al*. Possible association between PM_2.5_ and neurodegenerative diseases: A systematic review*. Environ Res* 2022;208:112581.

[25] Dhiman V, Trushna T, Raj D, Tiwari RR. Is Air Pollution Associated with Increased Risk of Dementia? A Meta-Analysis of Epidemiological Research*. Neurol India* 2022;70:1004–19.

[26] Fu P, Guo X, Cheung FMH, Yung KKL. The association between PM2.5 exposure and neurological disorders: A systematic review and meta-analysis*. Sci Total Environ* 2019;655:1240–8.

[27] Gong Y, Zhang X, Zhao X, Chang H, Zhang J, Gao Z *et al*. Global ambient particulate matter pollution and neurodegenerative disorders: a systematic review of literature and meta-analysis*. Environmental Science and Pollution Research* 2023;30:39418–30.

[28] Meo SA, Shaikh N, Alotaibi M, AlWabel AA, Alqumaidi H. Effect of air pollutants particulate matter (PM2.5, PM10), sulfur dioxide (SO2) and ozone (O3) on cognitive health*. Scientific Reports* 2024;14:19616.

[29] Mohammadzadeh M, Khoshakhlagh AH, Grafman J. Air pollution: a latent key driving force of dementia*. BMC Public Health* 2024;24:2370–4.

[30] Peters R, Mudway I, Booth A, Peters J, Anstey K. Putting Fine Particulate Matter and Dementia in the Wider Context of Noncommunicable Disease: Where are We Now and What Should We Do Next: A Systematic Review*. Neuroepidemiology* 2021;55:1–13.

[31] Power MC, Adar SD, Yanosky JD, Weuve J. Exposure to air pollution as a potential contributor to cognitive function, cognitive decline, brain imaging, and dementia: A systematic review of epidemiologic research*. Neurotoxicology* 2016;56:235–53.

[32] Tsai T, Lin Y, Hwang B, Nakayama SF, Tsai C, Sun X *et al*. Fine particulate matter is a potential determinant of Alzheimer's disease: A systemic review and meta-analysis*. Environ Res* 2019;177:108638.

[33] Weuve J, Bennett EE, Ranker L, Gianattasio KZ, Pedde M, Adar SD *et al*. Exposure to Air Pollution in Relation to Risk of Dementia and Related Outcomes: An Updated Systematic Review of the Epidemiological Literature*. Environ Health Perspect* 2021;129:96001.

[34] Xu H, Zhou X, Xu S, Fang Z, Li C, Lv Y *et al*. Exposure to air pollution and cognitive function based on the minimum mental state examination: a systematic review and meta-analysis*. Int J Environ Health Res* 2025;35:1958–71.

[35] Yang Z, Mahendran R, Yu P, Xu R, Yu W, Godellawattage S *et al*. Health Effects of Long-Term Exposure to Ambient PM2.5 in Asia-Pacific: a Systematic Review of Cohort Studies*. Current Environmental Health Reports* 2022;9:130–51.

[36] Yu X, Zheng L, Jiang W, Zhang D. Exposure to air pollution and cognitive impairment risk: a meta-analysis of longitudinal cohort studies with dose-response analysis*. Journal of Global Health* 2020;10.

[37] Yuan A, Halabicky O, Rao H, Liu J. Lifetime air pollution exposure, cognitive deficits, and brain imaging outcomes: A systematic review*. Neurotoxicology* 2023;96:69–80.

[38] Killin LOJ, Starr JM, Shiue IJ, Russ TC. Environmental risk factors for dementia: a systematic review*. BMC Geriatrics* 2016;16:175.

[39] Wilker E, Osman M, Weisskopf M. Ambient air pollution and clinical dementia: systematic review and meta-analysis*. BMJ* 2023;381:e071620.

[40] Zhao T, Markevych I, Romanos M, Nowak D, Heinrich J. Ambient ozone exposure and mental health: A systematic review of epidemiological studies*. Environ Res* 2018;165:459–72.

[41] Debelu D, Dechasa AM, Aschalew A, Mengistie B, Deriba W. Global Public Health Implications of Traffic Related Air Pollution: Systematic Review*. Environ�Health�Insights* 2024;18:11786302241272403.

[42] Clark C, Crumpler C, Notley H. Evidence for Environmental Noise Effects on Health for the United Kingdom Policy Context: A Systematic Review of the Effects of Environmental Noise on Mental Health, Wellbeing, Quality of Life, Cancer, Dementia, Birth, Reproductive Outcomes, and Cognition*. International Journal of Environmental Research and Public Health* 2020;17:393.

[43] Thompson R, Smith RB, Bou Karim Y, Shen C, Drummond K, Teng C *et al*. Noise pollution and human cognition: An updated systematic review and meta-analysis of recent evidence*. Environ Int* 2022;158:106905.

[44] Hegewald J, Schubert M, Freiberg A, Romero Starke K, Augustin F, Riedel-Heller S *et al*. Traffic Noise and Mental Health: A Systematic Review and Meta-Analysis*. International Journal of Environmental Research and Public Health* 2020;17:6175.

[45] Byun G, Choi Y, Foo D, Stewart R, Song Y, Son J *et al*. Effects of ambient temperature on mental and neurological conditions in older adults: A systematic review and meta-analysis*. Environ Int* 2024;194:109166.

[46] Olayinka O, Olayinka O, Alemu B, Akpinar-Elci M, Grossberg G. Toxic Environmental Risk Factors for Alzheimer’s Disease: A Systematic Review*. Aging Medicine and Healthcare* 2019;10:4–17.

[47] McGrattan AM, Zhu Y, Richardson CD, Mohan D, Yee CS, Sajjad A *et al*. Prevalence and Risk of Mild Cognitive Impairment in Low and Middle-Income Countries: A Systematic Review*. Journal of Alzheimer’s Disease* 2021;79:743–62.

[48] Rodrigues PMF, Delerue-Matos A. The effect of social exclusion on the cognitive health of middle-aged and older adults: A systematic review*. Arch Gerontol Geriatr* 2025;130:105730.

[49] Wang A-, Hu H-, Ou Y-, Wang Z-, Ma Y-, Tan L *et al*. Socioeconomic Status and Risks of Cognitive Impairment and Dementia: A Systematic Review and Meta-Analysis of 39 Prospective Studies*. The Journal of Prevention of Alzheimer's Disease* 2023;10:83–94.

[50] Wu Y, Prina AM, Brayne C. The association between community environment and cognitive function: a systematic review*. Soc Psychiatry Psychiatr Epidemiol* 2015;50:351–62.

[51] Kuiper JS, Zuidersma M, Zuidema SU, Burgerhof JGM, Stolk RP, Oude Voshaar R,C. *et al*. Social relationships and cognitive decline: a systematic review and meta-analysis of longitudinal cohort studies*. Int J Epidemiol* 2016;45:1169–206.

[52] Kuiper JS, Zuidersma M, Oude Voshaar RC, Zuidema SU, van den Heuvel ER, Stolk RP *et al*. Social relationships and risk of dementia: A systematic review and meta-analysis of longitudinal cohort studies*. Ageing Research Reviews* 2015;22:39–57.

[53] Plassman B, Williams J, Burke J, Holsinger T, Benjamin S. Systematic Review: Factors Associated With Risk for and Possible Prevention of Cognitive Decline in Later Life*. Ann Intern Med* 2010;153:182–93.

[54] Hamrah MS, Bartlett L, Jang S, Roccati E, Vickers JC. Modifiable Risk Factors for Dementia Among Migrants, Refugees and Asylum Seekers in Australia: A Systematic Review*. Journal of Immigrant and Minority Health* 2023;25:692–711.
